# Primary Literature as the Course Design Centerpiece in a General Chemistry Course That Prepares Undergraduates for Research

**DOI:** 10.1021/acsomega.5c07586

**Published:** 2025-12-08

**Authors:** Timm A. Knoerzer, Kimberly A. Gardner, Mark D. Reimann, Barry W. Hicks

**Affiliations:** 2611 United States Air Force Academy, Department of Chemistry, 2355 Fairchild Drive, Suite 2N-225, United States Air Force Academy, Colorado 80840, United States

## Abstract

Over the past several decades, research has become a key component of the undergraduate curriculum. Indeed, there have been considerable efforts to prepare undergraduates to engage in authentic research experiences with educators developing various curricular and pedagogical changes as the catalysts for effective preparation. However, most institutions elect to innovate in the major-level courses, while remaining committed to the traditional approach in general chemistry that was designed decades ago and took hold in the 1950s. It is our assertion that this outdated approach limits the ability of undergraduates to gain early access to research while also diminishing the learning gains experienced by students once they begin in their research endeavor. Therefore, in order to provide an alternative and potentially innovative approach to General Chemistry undergraduate education, we have explored the development of a combined lecture-laboratory course at the US Air Force Academy that emphasizes a research-forward, primary literature-based design to in lieu of the traditional textbook approach. This course emphasizes the extensive use of primary literature that is overtly connected to a series of experiments as the major emphasis in the course. As such, this course leans predominantly toward a laboratory-based method of instruction (∼70%). What follows is a description of a course, including methodology for changing the general chemistry curriculum from a course focused on distributing knowledge to one designed around introducing students to the academic chemical research process. This approach facilitates student learning relative to research, inquiry, and scientific reasoning, allowing such a curriculum format to be used for the achievement of relevant, *in situ* learning of chemistry.

## Graphical abstract

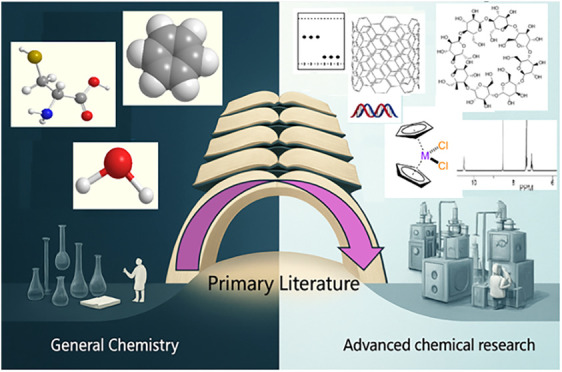

## Introduction

Considerable effort has been undertaken in recent years toward addressing how to innovate the undergraduate educational experience for students in chemistry courses. These efforts have been driven by several factors including the decline in numbers of students pursuing chemistry, outdated instructional methods, and the need to provide key 21st century knowledge and skills for the next generation of scientists.^1,2^ Much of the investigative attention has been directed toward advances in the delivery of instruction in general chemistry.^3–7^ It is true that at most US colleges and universities, the majority of students taking general chemistry do not major in chemistry. Often, this remains true in more advanced sophomore organic chemistry courses.^8^ Furthermore, many chemistry majors end up working in careers outside of chemistry.^9^ However, there are key learning outcomes that our institutions endeavor to achieve in all students. The US Air Force Academy (AFA) is no different, but our institution has placed particularly strong emphasis on the development of scientific reasoning skills. As such, the general chemistry course is obligated to provide enrichment of this outcome. At the same time, faculty are invested in providing the best possible foundation for those who will decide to major in chemistry. This two-pronged expectation to educate both the chemistry major and the nonmajor needing chemistry-specific training leads to a conundrum. How do we design a course that best educates both nonmajors needing foundational chemistry expertise and the future chemistry major who may ultimately pursue a graduate degree in chemistry? Even more importantly, we ask what we can do educationally to better prepare all students for the scientific reasoning skills needed for the 21st century learner? Finally, what type of introductory course will enable chemistry majors to develop the knowledge, skills, and experience needed to become excellent undergraduate researchers?

*It is our thesis that there is unparalleled value in reading the primary literature in chemistry that cannot be gained from textbooks*, and that well-prepared college freshman can learn general chemistry in a course that utilizes primary literature in lieu of a standard textbook. Furthermore, we assert that is the *best method to prepare undergraduate students for research* while still cultivating key ideas in chemistry. Perhaps most importantly, an introduction to a broad range of chemistry literature can help to *establish true scientific literacy as well as sound scientific reasoning skills*, *which are integral to the research process*.^10^ Finally, in a course designed around primary literature, students in general chemistry will acquire an appreciation of the role of chemistry as a central science while experiencing chemistry in authentic, inquiry-based contexts. This can be done without sacrificing most of the skill and knowledge expected from a traditional course and without relying predominantly upon a commercial textbook.

Regarding textbooks, it should be noted here that most colleges and universities still use one of the standard general chemistry textbooks in their standard as well as honors freshman general chemistry courses, and use a traditional curriculum based primarily upon the textbook selected. Given that virtually every general chemistry textbook on the market today is a dressed-up, modernized, and watered-down version of Linus Pauling’s *General Chemistry*,^11^ which, in Pauling’s own words “...is designed especially for the use by first-year college university students who plan to major in chemistry or closely related fields...,” and are thus intended to prepare students for upper division chemistry courses, where does that leave us? While many recognize the need to change what we’re doing, only a few concrete examples are available that can be universally applied to address the problem of genuine curriculum reform.^3–7^ The problem has been articulated previously as, “a few years later nothing more is remembered of the course, in all probability, except that it was unreasonably stiff, that the smells in the laboratory were terrible, and that the formula for water is H_2_SO_4_”.^12^ We have experienced the same at the AFA wherein many of our best and brightest incoming students performed disappointingly on general chemistry final examinations, retained relatively little knowledge or skills after two semesters of the course, and left the course with the same disdain for chemistry so commonly seen by nonmajors.^13^

Therefore, we envisioned a project for designing a general chemistry course for undergraduate students that better serves them to develop the chemical thinking and reasoning skills that they will need to engage more completely in scientific thought, to more enthusiastically study the discipline, and to more effectively prepare students to participate in undergraduate research investigations. This is not a completely novel idea and has been already employed in advanced chemistry courses to great effect.^14^ It is our assertion that a primary literature approach best facilitates the type of thinking we value and provides the key skills that best advance undergraduate research development. More importantly, we think that this approach can be effectively used to provide a solid foundation in general chemistry courses. Our assertion is further advanced by a previous report highlighting not only the importance of integrating literature into the chemistry curriculum, but also the relative ease of doing so in the age of open access to scientific publications.^15^ Although many have published examples of using primary literature in advanced undergraduate courses such as organic, analytical, physical, inorganic or biochemistry,^16–21^ very few have attempted to use journal articles in freshman general chemistry.^21–25^ In most of those examples, primary literature was used for only one, or a few, assignment(s),^21–24^ or merely for examinations.^25^ However, some more recent papers have described the integration of primary literature in chemistry and biology courses with the explicit objective of improving student scientific literacy skills.^26,27^ These precedent studies are in alignment with our course objectives and serve as further validation that a course designed around primary literature may serve to enhance scientific reasoning and literacy skills in addition to developing student understanding of chemistry content. Finally, two additional studies have been published since our initial exploratory study described here that demonstrate the potential effectiveness of a course designed around the use of primary literature.^28,29^ The first of these studies^28^ accentuates the ways in which student learning can be enhanced in relation to a traditional instructional approach including advancement of exposure for students to real-world chemistry problems that afford rich data sets that can be assessed and argued. This leads to a greater understanding of the nature of science which is often discounted in a traditional general chemistry curriculum. The second study demonstrates a more formalized CREATE approach to assisting students in the reading and analysis of research articles,^29^ which could serve as a facile method for adopters of using scientific literature as a key curricular design element.

However, even with the considerable advancement in course design and pedagogy, a gap still remains in affording the effective methods of instruction that allows students to learn key chemistry ideas while also supporting the development of research acumen early in the undergraduate educational experience. Furthermore, the traditional approach of education based upon extraction of information from traditional textbooks does not readily produce the opportunities to engage in research-style investigations in an authentic way. Therefore, herein, we propose a much more extensive use of a broad range of “chemical” literature early and often, and in both the lecture and the laboratory, as the centerpiece of the general chemistry curriculum. What follows is a description of the evolution of a course designed around a nonstandard book (not a textbook) and primary literature for a “cultural study” in chemistry that lays a solid foundation to prepare students for undergraduate research. However, we believe this would also be a more effective way to prepare the nonmajor as well in a way that fortifies sound chemical thinking versus a traditional chemical concept-oriented general chemistry course leading to better understanding of scientific inquiry and the nature of science. While this course was originally developed for an honors course, we are advocating a similar structure for use, with some modification, in more conventional general chemistry courses.

## Course Design Methods

### Course Participation Methods

The course was envisioned as a laboratory-forward design with heavy integration of primary literature and minimal curricular consideration from a traditional textbook. The initial determination was to offer two sections of approximately 15–20 students each for a total course enrollment of approximately 30 students maximum to ensure ample engagement in small sections with heavy emphasis on discussion and collaboration. The course was also designed to satisfy the requirements of the Academy Scholars program with a select group of students. Course enrollment selection was initially based upon a set of standard AFA academic profile information and not as a course targeting chemistry majors. Incoming freshmen at the AFA are ranked based upon high school GPA, standardized test performance (SAT or ACT), and other academic inputs (quality of the high school, extracurricular activities such as athletics and student leadership, etc.). The top 5% of the incoming class was then further ranked based upon performance on the chemistry placement test (ACS California Chemistry Diagnostic Test), and some additional consideration was given to those students who had taken the AP Chemistry test. A list of 30 prospective students was assigned to two sections of 15. On the first lesson, the students were told that participation in the course was voluntary and offered an opportunity to drop the course. Although several students expressed trepidation each year the course was offered, and about whether they belonged in the course, less than one student per year opted out of the program.

The first iteration of this course (V1) was taught in our Scholars General Chemistry Course (Honors) during the 2008–2009 academic years. A second version of the course (V2) was implemented during the 2010–2013 academic years, a decision driven by a change in course emphasis toward organic chemistry. As a Scholars course, this offering was configured to achieve explicit expectations such as (a) course administration should be student-centered, (b) content focused on enduring works rather than textbooks, (c) student engagement via active learning and discussion as a primary pedagogy, and (d) learning focused on “high” levels of the Taxonomy of Educational Objectives. Of the students that completed the course, less than 10% of the students failed to meet the course expectations (weaker performers). Course feedback from cadets in the course indicates that the weaker performers are those with the weakest incoming preparation. Therefore, during the latter years of offering this course, the instructional team determined that additional criteria may be considered in order to best align the student population with the rigor of the course: completion of (a) AP chemistry in high school with an AP score of 4 or 5, (b) a third semester of high school chemistry course ideally during the senior year, (c) college chemistry course (1 semester minimum), and (d) minimum 4 high school level science courses. In our opinion, any student with the requisite science background could be successful in this course regardless of chosen academic major. Furthermore, the *depth and scope* of the course could be readily adapted to relative audience preparation level in order to still achieve the core developmental objectives as they pertain to an investigative chemistry course focused on primary literature.

### Course Materials

Materials were comprised of (a) selected primary literature, (b) primary literature summaries, (c) supporting texts, (d) chapter supplements, (e) PowerPoint mini-lesson presentations, and (f) select laboratory experiments and projects. Careful consideration was made to synergize these components around *six central themes* in general chemistry (nomenclature, reactions, stoichiometry, structure, thermodynamics and kinetics) and to provide complementary scientific literature to the hands-on laboratory engagements.

This course does not utilize a traditional textbook, but rather depends heavily on carefully selected primary literature that is augmented with a more generalized chemistry-focused book that emphasizes real-world chemical applications. In this vein, two books have been used in this course, Caveman Chemistry^30^ and Napoleon’s Buttons.^31^ The selection of primary literature is the most challenging aspect of developing a course like this one. Indeed, a great deal of time and energy is devoted to selecting the most accessible primary literature for undergraduates. The extensive selection process is described in detail in the Results and Discussion section, but authors also developed literature summary assignments to facilitate the reading and analysis that each student was asked to complete for each assigned article. In addition, the authors have developed chapter supplements and PowerPoint presentations to highlight key chemistry content that is related to the primary literature and that supports the laboratory investigations that students undertake in the course. These resources provided additional support for students to learn the key chemistry content from the readings and experiments.

Laboratory investigations arose from the Caveman Chemistry text, from variations and extensions of traditional experiments, or from the primary literature itself. A major focus was placed on developing project-based experimentation that resembled research-style investigations. Some of the literature-based experiments overtly were discovery-based without any sort of predetermined/expected outcome.

## Results and Discussion

### Course Books and Chapter Supplements

While it is certainly possible to administer a general chemistry course without any book, we found that choosing a book provided a scaffolding upon which the primary literature could be selected and tell a more unified story, as well as provide direction for potential laboratory exercises. In the first version of the course (V1), we selected Kevin Dunn’s *Caveman Chemistry* ^30^ as the course book and included about 1–2 papers for every chapter (34 papers the first year, 16 the second year) in our Honors Chemistry sections. We chose Dunn’s book for two reasons, it put development of chemical technology into a historical perspective, and we enjoyed the liberal use of humor. Beginning in the fall of 2010, a second version of the course (V2) evolved in which the Le Couteur and Burreson’s book *Napoleon’s Buttons* ^31^ was used as the centerpiece text. Again, the reason for the change was a move toward content emphasis, which was more significantly focused on organic chemistry principles. While an *introductory* course using *Napoleon’s Buttons* has been described,^32^ they did not use it as a genuine course centerpiece, alongside integration of primary literature, and not in an [honors] general chemistry course or in a course geared toward preparation for research. It should be noted that any good book about chemistry could be used to augment a course with primary literature as the focal point as we have done. As an example, those listed in Table 1 came from a brief search of Amazon and Google Books by merely scanning tables of contents and indices.

**Table 1:** Books That Could Be Used to Focus Primary Literature in General Chemistry

title	author(s)	publication year
A Short History of Chemistry	J. R, Partington	1957
Guns, Germs and Steel	Jerod Diamond	1997
Mauve: A Color That Changed the World	Simon Garfield	2000
The 13th Element	John Emsley	2000
Transforming Matter: A History of Chemistry···	Trevor Levere	2001
**Caveman Chemistry** ^a^	**Kevin Dunn**	**2003**
**Napoleon’s Buttons** ^b^	**Penny Le Couteur & Jay Burreson**	**2003**
Uncle Tungsten: Memories of a Chemical Boyhood	Oliver W. Sacks	2003
Laughing Gas, Viagra, and Lipitor	Jie Jack Li	2006
Molecules That Changed the World	K. C. Nicolaou	2008
Molecules of Murder	John Emsley	2008
The Disappearing Spoon	Sam Kean	2011
Stuff Matters	Mark Miodownik	2015

a We used this book initially in our honors course for four years from 2008 to 2009.

b We used this book from 2010 to 2013. While most of the primary literature was replaced with case studies, including editorials, it is very easily amenable to adding more primary literature.

While we are not advocating any of them, we think the table shows that there is not now, nor is there likely to be a shortage of usable books in the future. Unlike most traditional general chemistry books that “drive the course,” the book merely provides the scaffolding around which the primary literature and laboratory exercises are chosen. We did not include any novels on the list, but a case could also be made for their use. The 2008 students were also asked to purchase a conventional text as a resource. Since a common student complaint was that it was a waste of money, this requirement was removed from subsequent course offerings.

Because the chemical principles in many of the books listed are normally insufficient for any general chemistry course or altogether ignored, each chapter was supplemented with a short chapter guide written by the instructors. Each chapter supplement was provided as a pdf file to be read immediately after the chapter, and each one focuses on how *six central themes* in general chemistry (nomenclature, reactions, stoichiometry, structure, thermodynamics and kinetics) are involved in materials found in that chapter (Supporting Information, pages S18–S25 for examples), along with the primary literature assigned for most chapters. Most also contain links to Web sites or databases related to chapter topics, and Wikipedia was used extensively. “Wiki” is very useful for demonstrating the difference between peer reviewed primary literature and an openly editable free-content encyclopedia. We think now that modern video-based options (e.g., YouTube) could also serve a functional role in supporting the primary literature approach of this course. Indeed, further evolutions of our experimental test sections of general chemistry during the 2021–2024 academic years support the value of web-based videos as a feasible support mechanism for student learning. It is our assertion that selecting a good book is helpful, ultimately the course’s success will not be due to the book used, but to the primary literature chosen to accompany that book.

### Lecture/Discussion Portion of the Course Design

At the Air Force Academy, the introductory chemistry courses (Chem 100 and Chem 200) use a combined lecture-laboratory design. Herein, these are 3 credit hour offerings which meet on a rotating M and T day schedule in a 2 h time block (see Supporting Information, page S17 for a representative US Air Force Academy semester schedule). Additionally, a sample course syllabus, unit learning objectives, and course schedule coordinated with the published Air Force Academy schedule is provided (Supporting Information, pages S2–S16). Indeed, this leads to an irregular and nontraditional type of course scheduling, but the course could be adapted to a more traditional MWF and TTh schedule if similar time blocks are available. Most likely, the common collegiate TTh configuration would allow for the extended time needed to execute a course like this one. In terms of execution, most days involve about 30–40 min of classroom time and 70–80 min of laboratory time. On some days, the entire period is devoted to laboratory work. The target balance across the semester is to devote about 30% of the total time to classroom activity and the remaining 70% to laboratory investigations. The classroom time is divided into three sections; 5–10 min for students to work in small groups to review of primary literature summary assignments (described later), about 10–15 min for student-led discussion of the primary literature article(s) (typically one, rarely two) associated with that chapter, 5 min to ask specific questions about the laboratory procedures to be performed that day, and a short, 10 min instructor-led mini-presentation (PowerPoint, see Supporting Information, pages S67–S68 for an example) on the background chemistry necessary to prepare them for the primary literature accompanying the upcoming chapter. In the first year, we found that if students did not understand the instrumental techniques used to generate data in the papers selected, they struggled with comprehending other sections of the paper, so we generated brief PowerPoint presentations (typically 7–10 slides covered at a rate of 1–2 min) to explain those techniques. It is important for any adopter of a course like this one to engage in continual refinement of these presentations. Careful and strategic modification will allow the adopter to pivot the course content to meet emerging curricular needs and to integrate new experimental content. Perhaps, even more important is for adopters to consider the relative background preparation the students bring to the course. When needed, chapter supplements can be used to fill knowledge gaps and to better orient students to the skills and content expected in the course. Indeed, there can be considerable investment on the part of instructors for developing course content, but to better assist adopters, additional resources like chapter summaries and PowerPoint presentations for the two course versions (V1 and V2) are **available upon request with the contact author**.

### Laboratory Portion of the Course

It is our perspective that any course that uses primary literature and focuses on developing research skills should also be laboratory-forward in design. The hands-on, active learning dimension of laboratory experimentation is vital to adequately preparing students for undergraduate research. The laboratory experiments were done either individually (for about half of the laboratories), with a single lab partner (for about half of the laboratories), and a few projects were done in groups of 3–4 students. In the first year, students were given credit when they could accomplish the project described in Dunn. We believe that this method of earning credit helps to fortify the results-oriented objective that we hope to infuse in our students. Even in that year, we made several substitution projects when we felt the Caveman project did not include enough chemistry. For example, instead of making glass arrowheads as described in Dunn’s chapter 2 on silicates and other minerals, we did a spectrophotometric determination of copper in malachite; instead of making string from raw wool as described in Dunn’s chapter 6 on textiles, we made biocidal cotton essentially as described in a research paper^33^ or biocidal Kevlar.^34^ This continued the second year when instead of making sulfuric acid, we tested fruits for anthocyanin content.^35^

While the Dunn book is interesting, unusual, and filled with humor, it provides too much detail for the laboratory portion of the course to be inquiry-guided as executed in our setting. In fact, during the second year we found that we had substituted projects for so many of the Dunn chapters that we no longer wanted to use a book for the laboratory portion of the course. In addition, once the Le Couteur book was adopted, additional experiments were identified and used for about half of the book chapters, which was necessary because Napoleon’s Buttons does not include a laboratory portion per se. Furthermore, additional experiments on materials and energy were also developed in-house to allow for a comprehensive laboratory experience to be realized for this course (Table 2). As with the Caveman book, the underlying chemistry in this text is far too simplistic for a college level course, so chapter supplements were again provided for students to obtain a more detailed exposure to the underlying chemistry. As such, all of the experimental examples that we have used throughout the course development are **available upon request with the contact author**. However, it is vital to know that considerable effort went into the development of the laboratory experience in this course. Inherent to this process was the continuous cycle of experimental adoption, implementation, refinement/modification, and reimplementation. This fine-tuning approach is needed when engaging students in noncookbook approaches in the laboratory. Also, a common misconception (pushback) that we encountered was the idea that students could not execute more complex research-oriented investigations in the first-year laboratory. However, we directly challenged that idea and have discovered that our students engaged more vigorously in these types of investigations, often finding higher levels of student satisfaction and growth (see SALG results below). We encourage adopters to consider push the envelope and to be as creative as possible when considering what types of experiments to integrate into a course of this design.

**Table 2:** Representative Set of Experiments Used in the Primary Literature Course

experiment number	topic	source	experiment description
1	Solubility	Buttons Ch1	Extraction of Eugenol from Cloves or Nutmeg with Liquid CO_2_
2	Vitamins	Buttons Ch2	Iodometric titration for VitC content of fruits and vegetables
3	Stereochemistry	Buttons Ch3	Optical Activity of Carbohydrates
4	Explosives	Buttons Ch5	Production of Cellulose Nitrate
5	Polymers	Buttons Ch6	Synthesis and Tensile Strength Testing of Nylon
6	Polymers	Buttons Ch6 In house	Production and Tensile Strength Testing of Transgenic GFP Silks
7	Dyes	Buttons Ch9	Synthesis and Characterization of Azo Dyes
			**Inorganic Materials Chemistry**
8	Composite Materials	In house	Kevlar-Polymer Composites as Bullet Proof Materials
9	Ceramics	Caveman	Making Ceramic Crucibles & Ceramic Armor
10	Metals/Alloys	Caveman	Metallurgy: Making Bronze from Copper and Tin Ores
			**Energetic Materials**
11	Fuel Chemistry	In house	Making and Characterizing Biodiesel and its Blends with JP8
12	Nuclear Chemistry	In house	Mapping Radon Distributions at the USAFA

Except for research projects, most use of primary literature in the undergraduate setting has concentrated on understanding the theory of the work, and virtually never trains students to read the “Materials and Methods” sections critically. Teaching (honors) general chemistry students how chemists truly communicate what they’ve done in the laboratory is a valuable exercise, which provides a perspective about chemistry that cannot be obtained from the sterilized presentation in textbooks, or by doing a “canned” laboratory exercise. Sadly, this is something we almost never teach to either majors or nonmajors. We thoroughly agree with James Conant that, “The stumbling way in which even the ablest of the early scientists had to fight through thickets of erroneous observations, misleading generalizations, inadequate formulations, and unconscious prejudice is the story which...needs telling”.^36^ Anyone who has ever tried to replicate a portion of a published paper at the bench knows that it is not always straightforward. For example, the biocidal fabrics and anthocyanin projects demonstrate how primary literature can be integrated into the laboratory portion of the curriculum. For those projects, students had to critically examine the “Materials and Methods” sections of papers to generate their own procedures *prior to class*. They had to pay attention and modify procedures for materials and equipment that we possess at our institution. We then came to class and went over the various proposals in a group setting to generate a more accurate draft procedure. This approach was an extremely valuable part of the course as it allowed students to more fully engage in method development, a skill that we think is under-developed in novice undergraduate science students. More importantly, this approach allowed a utilitarian tension to develop between this course and the more traditional course offerings in our department. This tension was documented by student comments in the traditional courses that would often be stated as “why can’t we be doing what the students in the honors course are doing?” Indeed, gaining buy-in from faculty members who are accustomed to the traditional methods of experimentation in general chemistry via “cookbook” approaches may be a challenge, At the Air Force Academy, this tension exists between civilian and military faculty, wherein the civilians are more apt to instruct a course like this one that requires improvisation and ill-defined outcomes.

Finally, the use of such projects in course undergraduate research experiences (CUREs) would also be possible.^37^ For production of biocidal fabrics, for example, it is done by modifying acrylamide or methyl acrylamide polymers with bleach to make N-halamines. There are dozens of relatively inexpensive acrylamide analogs that could be used and tested for greater efficiency with the same general procedure. In our practice of such research experiments, once students had produced a draft procedure, we contacted the authors of the papers and asked them to examine, critique and suggest shortcomings of the student’s draft procedures and forwarded those comments to the students. This approach cultivates an important perspective for our students in the sense that there is a community of scholars conducting research investigations and that they collaborate on the science.

### Choosing Primary Literature to Accompany Each Chapter

Because reading primary literature is emphasized, and because this is the AFA, the first day of class includes a pretest on a paper sent the night before on the development of coal-based jet fuels (Supporting Information, pages S26–S31). This not only introduces them to Air Force-relevant topic, but it also can be used as a benchmark of incoming student capability. It should be noted that one possible modification is to use this assessment artifact as a postassessment as well, something we implemented in the latter years of the course to further fortify our assessment process. It should be noted that we have opted to retain the original paper to provide better continuity between the pre- vs postassessment artifacts. Another modification is to change the paper choice for this assessment strategy on a yearly basis ostensibly generating a 3–4 year rotational schedule. This would allow an adopter to avoid the possibility of academic collusion on the part of the students. Additionally, the first lessons are dedicated to the use of resources such as PubMed, SciFinder and Google Scholar, and how to read a journal articles. To this end, we ascribe to the read-analyze-write approach pertaining to journal articles as described in the text “Write Like a Chemist,” which serves to assist students in the overall organization, tone, audience, writing conventions, and scientific content present within primary literature.^38^ Following on to this orientation to scientific articles, students then are guided through the read-analyze-write process for each chosen article by completing a primary literature summary (see Supporting Information, pages S69–S74 for examples linked to specific articles). These worksheets are completed as homework while the student reads and analyzes the chosen journal article. Students are tasked to identify keywords and key vocabulary as well as extracting critical directed information from the introduction, methods, results, and discussion sections. Students also are afforded the opportunity to reflect and critique the articles while also identifying specific chemistry-related content. These summary assignments serve as the starting point for the follow-on in-class discussions that are described later in this section and serve a vital role in preparing students for in-class engagement.

The papers and other course materials are made available on a shared network environment (e.g., Google Drive), sent as attachments to email, or from a course Teams page. Generally, directories containing materials for four book chapters at a time are released. This prevents students from trying to digest too much, too rapidly, and allows the instructors flexibility in altering the course direction as the semester progresses and to modify pacing as needed.

By far, the hardest, most time-consuming, highly interesting, highly rewarding, but occasionally extremely frustrating part of preparing a general chemistry course centered on primary literature is the selection of that literature. In Table 3, some of the criteria for selecting papers are listed in order of importance for our general chemistry courses. It is important to note that there is no perfect formula for choosing a good paper, and a great deal of subjectivity is involved. The subject of the paper should have obvious ties to the book chapter being used, and it usually is easy to find many papers that fit conceptually with the book chapter. The most difficult aspect of choosing an appropriate paper is finding one that is not too difficult for the students to understand. *Moreover*, *failure to choose appropriate papers will likely lead to failure of the course* to meet the objective of improving upon the content knowledge and skill in comparison with traditional general chemistry courses. Ideally, each paper will have multiple areas where the six general chemistry themes from the chapter supplement can be applied; if the instructor does not readily see these, you cannot expect the students to find them. Covering a wide range of topics with many different papers ensures that virtually every student will find at least a few of the papers more interesting and relevant to their individual academic aspirations.

**Table 3:** Criteria for Selecting Appropriate Primary Literature

title	author(s)
item to consider	ideal value
language	english
topic of paper	not overly complex obvious ties to book chapter
document type	nostly primary literature/some reviews/some educational journals
length	preferably less than 6 pages, 8–9 pages MAXIMUM
author(s)	available at contact information provided (preferably email address)
source	ACS journals preferred, but many exceptions can be made
number of instrumental techniques	1–2 ideally; never more than 3
citations	20 or more preferred except for very recent papers
publication year	generally >2000 (ideally within the past decade)
availability	pdf available from Google scholar or other online source
subject of the research	within the expertise of the course instructor(s)

Table 4 lists the 31 journals from which 45 papers were initially selected as possibilities for the first semester course; that list was eventually narrowed down to 32 papers (about two for each chapter), plus four other papers for the exams. We found that more than one paper per chapter was far too much of an expectation, even in the honor’s course, so we trimmed that down the second year to one paper per chapter, and covered fewer chapters (16 papers, plus papers for the exams and final).

**Table 4:** Full List of Journals Used in the First Course Iteration (2008)

title
Archaeometry
Chemical Biology^a^
Chemical Research Trends in Microbiology Biotechnology Advances
Chemistry of Materials^a^
Conference on Non Destructive Testing of Art
Construction and Building Materials
Energy & Fuels^a^
Environmental Health Perspectives
Environmental Science and Technology^a^
Forest Products Journal
Geochemical Transactions
Gold Bulletin
Journal of Agricultural and Food Chemistry^a^
Industrial & Engineering Chemistry Research^a^
International Sugar Journal
Journal of Chemical Education^a^
Journal of Cultural Heritage
Journal of Material Science
Journal of the American Chemical Society^a^
Journal of the Science of Food and Agriculture
Metallurgical and Materials Transactions
Mineralogical Record
Nature Reviews: Genetics
Organic Letters^a^
Petroleum chemistry Division Preprints
Proceedings of the National Academy of Science, USA
Proceedings of the Royal Society
Science
Technology Journal of Antimicrobial Chemotherapy
The Journal of Neuroscience
The Royal Society of Chemistry

a Denotes ACS Journal.

Table 5 lists the titles of ten papers used in the fall of 2009 that represent the distilled volume of primary literature used in the first course iteration. Even a quick scan of these two tables shows clearly that a diverse range of topics are covered, and this helps in the objective of providing broader scientific literacy. Further examples have been collected in the 2010–2013 offerings of the course and continue to grow by having upper-division courses in our department that use primary literature (e.g., the organic chemistry laboratory course) share papers.

**Table 5:** Titles of Ten Representative Journal Articles Used in the Second Course Iteration (2009)

title
Progress toward coal-hazed JP900 (jet fuel)
Smoke and liquid smoke; study of an aqueous smoke flavoring from the aromatic plant *Thymus vulgaris* L
The earliest use of corundum and diamond in prehistoric China
The chemical composition of honey
Durable and regenerable biocidal polymer: acyclic N-halamine cotton cellulose
Evaporative evolution of a Na–Cl–NO_3_–K–Ca–SO_4_–Mg–Si brine at 95 °C; experiments and modeling relevant to Yucca Mountain Nevada
Microstructure and mechanical properties of microalloyed, high-strength transformation-induced plasticity steels
Skeleton of euplectella sp.: Structural hierarchy from the nanoscale to the macroscale
The Lycurgus Cup—a Roman nanotechnology
The carbon footprint of bio fuels-can we shrink it down to size m lime?

Because a formula for choosing individual journal articles for general chemistry does not already exist, we will describe our process; a flow diagram is provided in Figure 1. First, it is important to generate potential search terms from topics in the chapter. These terms are used to search SciFinder. If SciFinder is not available, then a good alternative is to search via Google Scholar or NCBI (National Center for Biotechnology Information), although the NCBI database is smaller and more directed toward biomedical applications.

**Figure 1:**
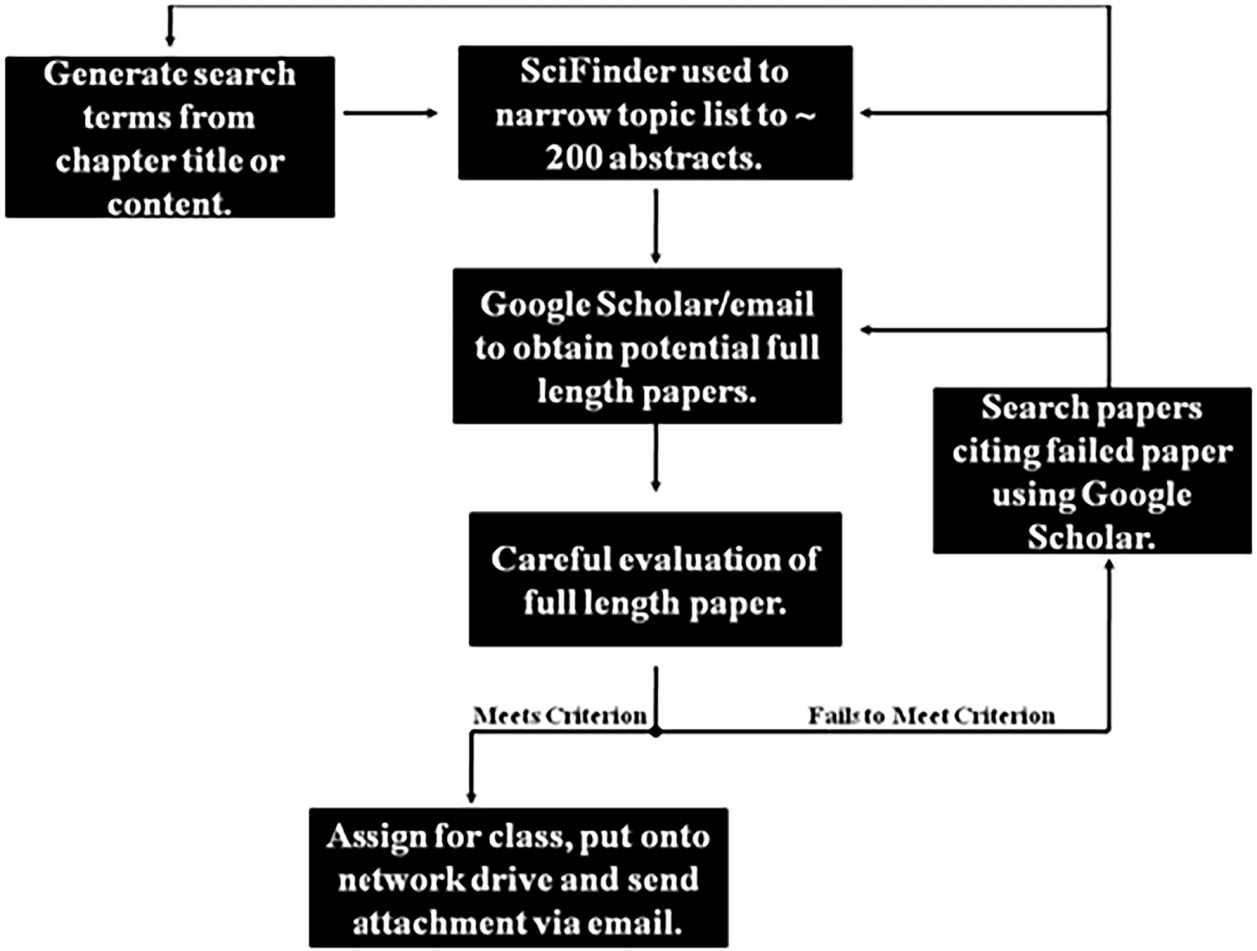
Process for selecting primary literature to accompany chapters in the book.

A good general approach is to restrict the search to only journal articles, only those published within the last 10–20 years (for current scientific studies), and only those published in English. The target is to have a “short list of about 200 articles from which further distillation can occur by searching for interesting titles and highlights within abstracts to identify articles that seem applicable. Once streamlined and identified, the selected articles are searched in Google Scholar (title/keyword) to access the pdf file, and to see the extent of the citation list. If the file is not available, we recommend contacting the corresponding author (email) or utilizing the campus library (e.g., interlibrary loan office) to request an electronic copy of the paper. Upon receipt of the article, it is imperative to *scan through the paper to assess fitness*. If that paper isn’t appropriate because it fails any of the Table 3 criteria, or seems too nebulous to describe (mostly trying to read from the students perspective to determine if the paper will be too difficult, and if there are sufficient applications of general chemistry that the students can recognize), a further search of reference citations from that original paper is conducted in Google Scholar. Typically, after about a 4–6-hour period, we can find a paper that is moderately acceptable. *It is obligatory to then read that “potential paper” in its entirety*. Failure to fully read a paper led on too many occasions to assigning papers that were thought to be appropriate from a scan but ended up being frustrating for students. One way around this is to narrow the scope and only require students to read the introduction or discussion, for example, but one must not make too liberal a use of this approach, or the value of seeing a paper as an integrated “research unit” is lost. We also like to point out that the entire paper, if it ever makes it into a textbook, will likely be, at most, a single paragraph. Tripling the amount of time described above almost ensures a good paper will be obtained. However, we must caution that we have occasionally spent over 12 h looking for an acceptable paper with nothing to show for the effort except a great deal of interesting material learned on the instructor’s part. It should also be noted that a similar process must be performed to obtain papers for the examinations. To assist the reader in recognizing good papers, some examples are provided (Supporting Information, pages S18–S66).

The first year we chose 42 papers—a single paper for about half the chapters and two papers for the other half of the chapters. This was far too demanding. In the second year we used only 14 of those from the first year because too many (even after severe scrutiny described above) were inappropriate, and we trimmed the number to about 22, covering only a single paper per chapter, by adding new (but, unfortunately, not always better) ones. By the third year, we limited the scope of papers to a single paper per chapter, resulting in a sum total of about 20 papers: one for each Chapter in Napoleon’s Buttons and one for each exam. As can be seen by the greatly varied journals sampled and topics discussed, a large variety of chemical literature is utilized. *One of the greatest successes of this course is that students leave with a true appreciation for how chemistry impacts so many other disciplines*, *even if they do not always fully understand all the literature*. *We do not think any general chemistry textbook imparts this lesson to the majors or nonmajors*, *certainly not as well as done by this method*. Building a good bank of potential papers is an ongoing process, and many of the papers used in the first two years were less than ideal. We find it acceptable that an occasional paper stretches students beyond their capabilities. Certainly, honors’ students can benefit from occasional challenges beyond their reach, however, especially in the first year, too many of the papers fit into that category. Our library maintains subscriptions to *Science* and *Nature*, and we have online access to all the ACS journals at our institution. So, we are always on the lookout for a better paper for every chapter. Even with this effort, we expect that it will take a newcomer about three years to get the body of primary literature to be what we would consider to be outstanding for the course. In addition, any adopter should avoid the misconception that once the primary literature library has been constructed, it is a finished project. Instead, we recommend continual modification of the library to promote interest in the subject matter and to maintain the most current scientific information. Because the literature is not static, the job of finding the best papers will continue to keep us abreast of the latest trends in chemistry. That is one side benefit from this course that cannot be underestimated. A primary literature-centered course keeps the instructor enthusiastically engaged in the course far more than one relying upon a traditional textbook, and this helps prevent instructor burn-out. Changing books does not mean the entire body of primary literature will necessarily change, most books in Table 1 have at least some common elements, and planning for a change 1–2 years in advance should make it possible to build a comparable course around any of several books.

Some of the papers we discuss in greater detail, and some in lesser detail, but all discussions are student-centered with only minimal prompting by the instructor. At this point, it is worth mentioning that we essentially are using a student-led, active learning style model to promote learning, a model that has seen widespread support in the chemistry education literature as an effective means of active learning.^39^ However, the use of this approach comes at a cost, meaning that we observe weaker/thinner earlier discussions, but that they improve significantly over the course of the semester as students take ownership of the method. Moreover, during the years spent developing this course, it was the decision of the course instructors to improve the impact of using primary literature with relative novices by connecting the selected journal articles to the experiments explored in the course. This allowed students to narrow the scope of chemistry investigated while also providing papers that were more directly relevant to the laboratory investigations being conducted. This change has positively influenced the student efficiency of reading and analyzing papers, while also improving the attitude of students because of the relevancy of the articles toward the course experimental work.

In terms of process, we ask that all the discussions begin with a “who, what, where” scenario as outlined in Table 6, then as mentioned earlier, the students are guided through the reading and analyzing process by use of primary literature summary worksheets. The worksheets afford consideration of chemistry content wherein students directly address where the central themes in the chapter supplements. However, one additional thing we ask is that they highlight and look up everything they do not understand (this is where Wiki can be valuable). This expectation is an incredible advantage for our course because the approach cultivates self-directed learning, which we believe is a typically absent skill for the undergraduate student, but vital for establishing the right attitude for research.^40^ However, this is also a time-intensive activity, so we expect that students do not spend more than 1–2 h on any one single paper. For some students on some papers, this means they will not even make it out of the introduction! However, students are afforded 5–10 min of class time to work within small groups to debrief the primary literature assignments in advance of the class discussion. This allows students to fill in some of the blanks before the floor is open for broader discussion. From an outcome perspective, we did observe that students made considerable gains as evidenced by their improved ability to read and discuss the papers over the course of the semester. Furthermore, this acquired skill by early undergraduates is expected to benefit them in upper division courses in the major, in their research experiences, and even in courses outside of chemistry.

**Table 6:** Who-What-Where Questions for Student-Led Discussions

question(s)
Who did the work and who funded the work?
Where was the work performed?
What is the main question the authors studied, and what were their main conclusions?
How were the experiments performed; what techniques were used?
Why would anyone be interested in this paper?
Which topics in this paper can you relate to principles of general chemistry?

Two of the best examples follow regarding how this course design most effectively impacted their understanding of how professional chemists read and benefit from primary literature. The first occurred when reading a 2008 *Science* article entitled “*Tough*, *Bio-inspired hybrid materials*,” written by Munch, Ritchie, and co-workers at the Materials Sciences Division, Lawrence Berkeley National Laboratory.^41^ Several students asked good questions which the instructors could not answer. We asked them how they should proceed, and, to their credit, they said, “contact the authors.” We immediately, in class, used the link from the pdf file to compose a brief email to the author. To our delight, he responded before class was over. His email was forwarded to both sections in the course so the students could see that chemists really are interested in discussing their research with other scientists who are interested in the topic and ask good questions. The second occasion occurred after assigning for the first examination a 2009 paper entitled “*Characterizing the binders in rock paintings by THM-GC-MS: La Casa de Las Golonrinas*, *Duatemal*, *a cautionary tale for radiocarbon dating*,” by Armitage et al. published in the *International Journal of Mass Spectrometry*.^42^ So many students emailed the author to ask questions in preparation for the examination, that she contacted us and offered to set up a Skype conference with the class to address all their questions more efficiently. It is highly unlikely that these scenarios would have ever occurred in a course using a traditional textbook. For any STEM students, we think these were invaluable learning experiences to better connect with the scientific community and to model the behavior that many research scientists exhibit in their daily scientific practice. As a matter of practice, however, we suggest allowing 1–2 days for authors to respond. Response rates are typically high (>90%), but on the occasion that an author does not respond within 1–2 days, students were directed to select a different paper and repeat the process. Students rarely had to go beyond a second or third selection to achieve a response (<10% of the time). So, even with a failure to respond, students could still gain access to author within a 1-week period with high success (∼95%).

Finally, we learned in the first year that if the experimental techniques used in the paper are not understood by the students, they are apt to not understand the results. Without understanding the results, they tend to fail to follow the paper’s discussion, taking very little from the primary literature for that chapter except that chemistry is “unreasonably stiff, that the smells in the laboratory were terrible, and that the formula for water is H_2_SO_4_”.^12^ For that reason, during the second year, we began doing 10 min mini-lectures covering the background necessary to understand the experimental techniques presented in the paper for the upcoming chapter. This is a key consideration for the course as the laboratory component is where students have the greatest potential to build their understanding of the nature of science, scientific reasoning, and laboratory investigation skills. A minimal investment in establishing the methods is deemed important to facilitate the establishment of sound fundamental research skills in the laboratory.

## Assessment

### Testing and Graded Events in Lecture & Laboratory

Two midterm examinations and a final examination were given each year. For all examinations, a paper (two for the final exam), from primary literature is provided several days in advance (always allowing at least 1 weekend between dissemination and testing so students will have time to examine it in detail if they choose, and even, perhaps, to ask questions of the authors). The test questions focus on the students’ abilities to read the paper and understand principles of general chemistry in the papers, and this approach has been previously described.^22^ An example exam with associated literature (Supporting Information, pages S32–S49) is provided. One of our course goals is to teach students that mastering a topic is worthy of credit in the course; when they master the understanding is less important. Therefore, after the first examination, we review most of the test in class, and students are given the option of scheduling an oral exam on any portion of the test they did not get correct for additional credit. The oral exam question is selected at random from any question not having full credit, and the student is “titrated” on that question. Based upon their ability to work through the oral questions, to apply material from other parts of the course, or to reframe prior experience, students are given some portion of the missing examination credit back. The point of this exercise is to teach students that full comprehension of any single paper requires prolonged and dedicated effort, and that the same principle applies to chemistry in general. We believe that this is a highly effective teaching practice, especially *postexam learning*, that helps to build the type of learning strategies that will lead to further success in the study of scientific disciplines.^43^

In addition to examinations, four written laboratory reports were required on the longer, more detailed projects (biocidal fabric, bioethanol, anthocyanins, synthesis and instrumental characterization of organic compounds). For each project, the *Journal of the American Chemical Society* format is used, and the comments we make are based upon the *Handbook for Authors of Papers in American Chemical Society Publications*.^44^ The goal of these reports was not only to enhance technical writing, but to teach the students to write a primary literature paper as a means toward enabling them to better understand the primary literature they are reading. This approach aligns with the read-analyze-write approach as outlined in the Write Like a Chemist text.^38^ In our opinion, a college-level course in science should challenge students to engage in the discipline in manners aligned with the practice of science and meet the demand of an undergraduate-level course expectation. Although a full paper was required for all four projects, the grading of each project focused on a different section of the paper as indicated in Table 7, while also allocating half of the total points on one specific section of grading emphasis.

**Table 7:** Portions of Each Laboratory Report and Journal Section Emphasized

section of paper	biocidal fabric	bioethanol	anthocyanins	synthesis and characterization of methyl orange and acetanilide
Title	√	√	√	**√**
Institutional Affiliation	√	√	√	**√**
Abstract				**√**
Introduction	**√** ^a^	√	√	**√**
Materials and Methods		**√** ^a^	√	**√**
Results			**√** ^a^	**√**
Discussion				**√** ^a^
Acknowledgments				**√**
References	**√** ^a^	√	√	**√**

a Indicates portion of the exercise given 50% of the grading weight.

After the first paper (biocidal fabric) is graded and returned, students must submit a second draft of that paper correcting any mistakes and addressing instructor comments in the Introduction and References sections. While they are expected to do a better job on the Introduction and References for the second paper (bioethanol), the grading emphasis for that paper is on writing the Materials and Methods section properly, and they must submit a second draft correcting any errors in that section of the paper. For the third paper (anthocyanins), the emphasis is placed upon the Results section; a second draft of that paper is also required with improvements in that section. On the final report, organic synthesis of dyes and drugs, the Discussion section is graded most heavily. This “building up” approach to *learning to**write**in the style of primary literature is invaluable in training students to be able to**read and analyze**primary literature*. In fact, we would argue that training to read and analyze primary literature without training to write it from one’s own data is almost not worthwhile and is supported by the *Write Like a Chemist* project.^38^ It is essential that the laboratory portion of the course reinforce the concept of learning to master primary literature. Compared to a traditional chemistry course, this is far greater preparation for doing effective undergraduate research than a traditional course. To validate the success of our approach, an example final report (Supporting Information, pages S50–S57) is provided. This report is from the Fall 2010 offering and is focused on the synthesis of modafinil keeping with the organic chemistry emphasis of this final report.

### Assessment and Student Evaluations

In addition to the course design described here, the Air Force Academy offers other standard core chemistry courses, notably Chem 110 and Chem 200. Both of these courses emphasize a traditional second semester chemistry curriculum with the Chem 110 course providing a deeper study of those topics with slightly more rigorous laboratory experiences. Neither course integrates primary literature in any way, nor do they engage students in discovery-based laboratory investigations. The Chem 110 course is comprised of chemistry and engineering students and is explicitly designed to educate students who intend to pursue STEM-heavy majors. Alternatively, Chem 200 is a core curriculum option that is populated by a much broader spectrum of students across the disciplines at the Air Force Academy.

In order to assess the relative fundamental chemistry understanding between the two standard courses (Chem 110 and Chem 200) and the one described here emphasizing the use of primary literature, the 2001 ACS general chemistry conceptual exam provided the ideal opportunity to do so. This analysis was conducted during the Fall 2012 offering of the primary literature-based course, at which the time the corresponding courses, Chem 110 and Chem 200, were also employing this same assessment artifact. As shown in Table 8, students in the primary literature course exceeded the scoring performance versus peers in both the Chem 110 and Chem 200 courses. Additionally, a closer look at the results shows that among the 28 students in the primary literature course, the lowest score was 35/60 correct responses, correlating to the 64th percentile in normed scoring. The highest score was 57/60 correct responses, correlating to the 100th percentile in normed scoring. Thus, the range of scores was 35–57 correct responses, with the average and median scores being 46 correct responses (91st percentile) and the standard deviation of 4.93. These results are not surprising given the selective makeup of the course population. Also, the results seem to negate the notion that a course of this type negatively impacts that ability of the students to demonstrate fundamental general chemistry knowledge, which is a common criticism of general chemistry curriculum redesign strategies.^3^ Given that little effort was made to prepare students specifically for the ACS general chemistry conceptual exam compared to the traditional course, and that students in the primary literature section learned a great deal that was not tested by that examination, we take the results to be sufficient evidence that, at the very least, *no harm was done by this course format*, *and indeed*, *they learned as much content knowledge in this course without making that a point of emphasis*. It is also worth noting that out of 30 students enrolled in this course during the first year, 10 later chose to major in chemistry, materials chemistry or biochemistry. This trend continued for the first several editions of the course. While recruiting students to major in chemistry was not a goal, the challenge that the best students found in this course drew them in more than any of the other core disciplines.

**Table 8:** Comparison of ACS Conceptual Exam Scores between the Primary Literature Course and More Traditional Chemistry Courses at the Air Force Academy

ave percentile score—primary literature course	ave percentile scots—Chem 110	ave percentile score—Chem 200
91st	84th	61st

Student Assessment of Their Learning Gains (SALG) results further validate the totality of learning experienced by students in the primary literature course. The results are summarized in Table 9 and they present the results for the SALG survey over a 3-year period (2010–2012) from the beginning of the semester (preterm) and at the end of course (post-term). These results are from a total of 118 students during the evaluation period. As shown in Table 9, some particularly relevant data stands out. First of all, the preterm data likely are overestimates of their level of skill as it pertains to the measured dimensions. This group is comprised of high achieving students who have taken considerable precollege science courses. The more interesting data are from the post-term. After completing this *rigorous* course (as supported by the comments from students), the self-assessment scores advanced in nearly all cases. Furthermore, these scores are likely more accurately reflective of the perceived gains made by the student cohort. Most importantly, these results consistently show excellent gains in learning that directly benefits the preparation of students for engaging in undergraduate research experiences.

**Table 9:** Evidence Collect from SALG Surveys Answering the Prompt: “Presently, I Can ________ As a Result of Taking This Course” (0-6 Point Scale)

dimension	pre-term mean	post-term mean
find articles relevant to a particular problem in professional journals or elsewhere	3.8	5.0
critically read and analyze articles about scientific issues	4.3	5.1
identify patterns in data	4.8	5.2
recognize a sound argument and appropriate use of evidence	5.0	5.2
develop a logical argument	4.9	5.2
use the language of chemistry	4.5	4.8
prepare and give oral presentation on scientific subjects	4.7	5.2
write professional quality laboratory reports	4.1	4.6
equate calculated results as reasonable and reliable	5.1	5.1
comprehend the scope of extremely large and small quantities	5.0	5.2
read tables: and graphs for information	5.5	5.4
make logical, appropriate, and convenient assumptions when solving complicated problems	4.8	4.9
construct new knowledge and skills from facts, data, and results	4.8	5.0
apply chemical models to explain experimental observations	4.8	4.8
apply chemical knowledge and skills to everyday life	4.4	4.6
critically analyze scientific findings reported in the media	4.2	4.7
connect what I know about science to what I learn in my other classes	4.7	5.0
apply my knowledge of science and scientific reasoning to civic and/or social issues	4.2	4.7
use systematic, scientific reasoning to solve problems	4.9	5.3
work with complex ideas	5.2	5.3
do chemistry laboratory work	4.8	5.0

The dimensions evaluated in Table 9 *comprise many of the key skills and attitudes that provide the foundation needed for research endeavors*. Also, traditional student surveys also reveal data indicating that students perceive the course to be well above the Air Force Academy average (6 point scale) in terms of satisfaction among technical courses (5.04 vs 4.35), number of hours dedicated to learning (3.26 vs 2.57), and instructor rating (5.56 vs 4.99). These measures suggest that students are heavily engaged in these courses, but are doing so with enthusiasm and commitment; an excellent combination for successful learning. These survey results may be further validated when compared to the gains made by the same 118 students in the preassessment versus postassessment for primary literature understanding. The assessment artifact is administered on the first and the last days of the course and a copy is available in the Supporting Information, pages S26–S31. The literature preassessment score averages 39/55 points (70.9%) while the postassessment score averages 47/55 points (85.5%). These gains demonstrate that even though students enter the course with some understanding of how to read and analyze a scientific paper, their skill level increases substantially by taking a course like this one. Perhaps more significantly, we are encourage by these gains made by first-year college students, which in our opinion lays a strong foundation for the scientific reading and writing development that occurs in upper division courses in our program.

In reflecting, we think Charles Dickens had presciently described the course more than a century earlier, “It was the best of times, it was the worst of times, it was the age of wisdom, it was the age of foolishness, it was the epoch of belief, it was the epoch of incredulity, it was the season of Light, it was the season of Darkness, it was the spring of hope, it was the winter of despair, we had everything before us, we had nothing before us, we were all going direct to heaven, we were all going direct the other way...”^45^ The difficulty inherent in reading primary literature meant that almost every student experienced, at times, the latter in each of Dickens’ contrasts. However, the sufficiently numerous successes also meant that every student likewise experienced the former. We think this is an acceptable, and even an expected, outcome. Perhaps most telling is that one year after taking the course every student responded to an informal question, that even given hindsight, they would still have chosen to take the considerably more difficult honors course using primary literature than our normal general chemistry course offering, or our advanced placement Chem 110 course for scientists and engineers. Also worth mentioning, is that when seniors majoring in chemistry were told about the course, they lamented: “Why didn’t we get to take a course like that?” A prior Chem 110S student captures a common sentiment among the population of students who have taken the course: “I liked the class a lot more than I’ve liked my other scholars’ [honors] courses, because it isn’t just discussion–it brings in a hands-on approach every single day.” Finally, a third representative response states: “The primary benefits that I got from taking 110S include exposure to real and in-depth scientific articles and abstracts. I was forced to study topics that required additional research on my part and my ability to approach and process scientific writing was improved far more than it would have been in a regular class. Although I often felt stretched beyond what I could accomplish, I think that being stretched allowed my analytic and laboratory skills to settle at a higher level than what they would have had the class only asked of us what we could fully complete.” On the negative side, one student said, “I can understand the fundamental chemistry, but I struggle in taking that basic knowledge into the lab and applying it.” This is certainly an expected challenge in a course like this one and to address that issue, we have continued to exploit the development of course materials to facilitate students making those connections. Other students have observed how tedious, time-consuming, and hard that the Scholars course is, but many have further indicated that their preparation (from high school chemistry, for example) failed to meet the starting point for our course. Here is representative comment along this vein: “I used to think I was really good at chemistry, but I now realize that was only because my other chemistry classes were barely scratching the surface—this was by far the hardest class I have ever taken.” However, even though the challenge exists, most students are satisfied that they took on the challenge. This sentiment is captured by this comment: “I have very much enjoyed this course, but glad it’s over.” As such, These are selected responses that have been continuously echoed among our students and help to validate the effectiveness of our approach. Indeed, it is our assertion that the primary literature focus of this course is a major catalyst to provide students with a learning environment that engages them in “freestyle” or “exploratory” learning much more effectively while moving away from the traditional “algorithmic” learning they are accustomed to experiencing. It is precisely this “freestyle” learning experience in our course that we believe best cultivates superior learning gains, while also achieving the type of thinking that best prepares students for further scientific study especially undergraduate research.

## Conclusion

If we want to prepare undergraduates for research in chemistry or related disciplines, the sooner they get a broad introduction to reading and writing primary literature, the more effective they will be at interpreting past research from an advisor, and the more effective they will be as beginning student researchers.^46^ This means we should not wait until upper division courses like biochemistry to access primary literature. Given that most students in our general chemistry are not going to be chemists, one might expect that such a nontraditional course would not be welcomed. However, SALG surveys indicated that nearly twice as many nonmajors found the course to be more enriching compared to the traditional course they had experienced in secondary school. And when students find value in what they are learning they tend to be more engaged and even retain more information.^47^ While this course format might be easier in institutions that do not have large sections sizes, large sections do not preclude this format since most laboratories are still done in smaller sections and the course delivery relies upon small group, active learning methods. Finally, as the use of primary literature in college courses becomes more prevalent, additional approaches are emerging to enhance the use of primary literature, even in the general chemistry curriculum.^48–55^ Finally, we also believe that this course emphasizes nicely the 10 facets of chemistry discussed in a recent paper by Talanquer, a goal not readily achieved in a traditional general chemistry course, but important to fortify the legitimacy of our approach.^56^ We believe that this observation further validates the commentary from Prof. Leonard Nash in 1976, that critically addresses the direction of the general chemistry curricular approach at that time, with many of the same observations holding true today.^57^ As a result of this lingering reality and that our department has resolved personnel availability to execute a course of this type, we will again be offering this course during the Spring 2026 semester following a long hiatus. Furthermore, a modernization of the course will be initiated leveraging the power of AI to allow the instructor to more efficiently devise questions related to the primary literature selected and for students themselves to read and analyze the papers with enhanced fidelity. This innovation should allow for more substantive discussions of papers and critical information extraction needed for laboratory investigations. This process could also be further enhanced by selecting articles from a single journal source (e.g., *J*. *Agric*. *Food Chem*. or *Org*. *Lett*.) in order to allow students to maintain a specific formatting and organizational familiarity. This limitation may allow for deeper overall growth by eliminating journal variability factors through a narrower scope of publications. Perhaps more importantly, the next iteration of the course will be populated with a broader audience of students. By extending this course to a more heterogeneous audience of students, we are directly challenging the possible misconception that this type of course can only be offered to honors students. Thus, the experiment continues, which we hope to describe in a future publication.

## Supplementary Material



## Data Availability

Additional resources are available upon request.

## Notes

Authors of this paper have provided key information that a prospective adopter might need to commence a course of this type: (a) course syllabus and schedule, (b) example exams with associated papers, (c) example student reports, (d) chapter supplement and PowerPoint examples, (e) an example set of primary literature used, and (f) a copy of the literature pretest used to assess the primary literature reading and analysis capacity of students (PDF)

## References

[1] Boerner L. K. (2024). Are Undergraduate
Chemistry Programs in Crisis. Chem. Eng. News.

[2] Price W. S., Hill J. O. (2004). Raising the Status
of Chemistry Education. Univ. Chem. Educ..

[3] Cooper M. (2010). The Case for
Reform of the Undergraduate General Chemistry Curriculum. J. Chem. Educ..

[4] Cooper M., Klymkowsky M. (2013). Chemistry,
Life, the Universe, and Everything: A New
Approach to General Chemistry, and a Model for Curriculum Reform. J. Chem. Educ..

[5] Sevian H., Talanquer V. (2014). Rethinking
Chemistry: a learning progression on chemical
thinking. Chem. Educ. Res. Pract..

[6] Talanquer V., Pollard J. (2017). Reforming a Large Foundational
Course: Successes and
Challenges. J. Chem. Educ..

[7] Collins J. S., Dickson-Karn N. M., Berhe M., Ogrin-Cotarlan S. (2025). Examining
Student Perceptions to Advance Systemic Reform in General Chemistry:
Prioritizing Course Structure and Curriculum. J. Chem. Educ..

[8] Gervay J. (1998). Perspectives
from a newly begun career. J. Chem. Educ..

[9] Ichniowski, T. C. Duke University Department of Chemistry. http://www.chem.duke.edu/~bonk/Careers/NonTrad.html, 2010.

[10] Reyes R. L. (2023). Exploring
science literature: Integrating chemistry research with chemical education. J. Chem. Educ..

[11] Pauling, L. General Chemistry; W.H. Freeman and Company: San Francisco, 1970.

[12] Kendall, J. Preface to General Chemistry; The Century Company: New York, 1927.

[13] Lippincott W. T. (1979). Why students
hate chemistry. J. Chem. Educ..

[14] Stewart J.
L., Bentley A. K., Johnson A. R., Nataro C., Reisner B. A., Watson L. A. (2018). Teaching
from the primary inorganic literature: lessons
from Richard Andersen. Dalton Trans..

[15] Pence H. E., Losoff B. (2011). Going beyond the textbook:
The need to integrate open
access primary literature into the Chemistry curriculum. Chem. Cent. J..

[16] Fikes L. E. (1989). Advanced
organic chemistry: learning from the primary literature. J. Chem. Educ..

[17] Mabrouk P. A. (1996). Planning a Day at PITTCON: An Introduction
to Current
Trends in Analytical Chemistry Research for Undergraduates. J. Chem. Educ..

[18] Francl, M. M. Walking the Tightrope: Teaching the Timeless Fundamentals in the Context of Modern Physical Chemistry. In Advances in Teaching Physical Chemistry, ACS Symposium Series; ACS Publications, 2007; Vol. 973, Chapter 15, pp 253–267.

[19] Baldwin M. J. (2003). A literature-based,
one-quarter inorganic chemistry laboratory course. J. Chem. Educ..

[20] MacDonald G. (2008). Teaching Protein
Purification and Characterization Techniques. A Student-Initiated,
Project-Oriented Biochemistry Laboratory Course. J. Chem. Educ..

[21] Moore W. E. (1972). Multidimensional
approach to teaching honors freshman chemistry. J. Chem. Educ..

[22] Sampey J. R. (1938). Acquainting
the undergraduate with the chemical library. J. Chem. Educ..

[23] Holme T. A. (1994). Providing
motivation for the general chemistry course through early introduction
of current research topics. J. Chem. Educ..

[24] Forest K., Rayne S. (2009). Incorporating primary
literature summary projects into a first-year
chemistry curriculum. J. Chem. Educ..

[25] Burness J.
H. (1996). A general
chemistry final exam based on the chemical literature. J. Chem. Educ..

[26] Ferrer-Vinent I. J., Bruehl M., Pan D., Jones G. L. (2015). Introducing Scientific
Literature to Honors General Chemistry Students: Teaching Information
Literacy and the Nature of Research to First-Year Chemistry Students. J. Chem. Educ..

[27] Krontiris-Litowitz J. (2013). Using Primary
Literature to Teach Science Literacy to Introductory Biology Students. J. Microbiol. Biol. Educ..

[28] Kovarik M. L. (2016). Use of
Primary Literature in the Undergraduate Analytical Class. Anal. Bioanal. Chem..

[29] Perla A. A., Hollar S., Muzikar K., Liu J. M. (2023). Using CREATE and
Scientific Literature to Teach Chemistry. J.
Chem. Educ..

[30] Dunn, K. Caveman Chemistry; Universal Publishers, 2003.

[31] Le Couteur, P. ; Burreson, J. Napoleon’s Buttons: How 17 Molecules Changed History; Tarcher/Putnam: New York, 2003.

[32] Samet C., Higgins P. J. (2005). Napoleon’s
buttons: Teaching the role of chemistry
in history. J. Chem. Educ..

[33] Liu S., Sun G. (2006). Durable and Regenerable
Biocidal Polymers: Acyclic *N*-Halamine Cotton Cellulose. Ind. Eng. Chem.
Res..

[34] Luo J., Sun Y. (2008). Acyclic *N*-Halamine Coated Kevlar Fabric Materials:
Preparation and Biocidal Functions. Ind. Eng.
Chem. Res..

[35] Wrolstad R.
E., Durst R. W., Lee J. (2005). Tracking color and pigment changes
in anthocyanin products. Trends Food Sci. Technol..

[36] Conant, J. On Understanding Science; Yale University Press: New Haven p 15.

[37] Bixby T. J., Miliauskas M. M. (2022). Assessment
of the Short-Term Outcomes of a Semester-Long
CURE in General Chemistry Lab. J. Chem. Educ..

[38] Robinson, M. S. ; Stoller, F. L. ; Costanza-Robinson, M. S. ; Jones, J. K. Write Like A Chemist; Oxford University Press, 2008.

[39] Yoder R. J., Bobbitt-Zeher D., Sawicki V. (2021). Understanding the use of student-centered
teaching methods in undergraduate chemistry courses. Res. Sci. Educ..

[40] Thompson T., Wulff S. (2004). Implementing guided self-directed
learning strategies (GSDL) in intermediate-and
advanced-level chemistry courses. Inter. J.
Self-Directed Learn..

[41] Munch E., Launey M. E., Alsem D. H., Saiz E., Tomsia A. P., Ritchie R. O. (2008). Tough, bio-inspired hybrid materials. Science.

[42] Livingston A., Robinson E., Armitage R. A. (2009). Characterizing the binders in rock
paintings by THM-GC–MS: La Casa de Las Golondrinas, Guatemala,
a cautionary tale for radiocarbon dating. Int.
J. Mass Spectrom..

[43] Schroeter C., Green V. S., Bess E. (2010). Second time is a charm:
The impact
of correcting missed exam questions on student learning. NACTA J..

[44] Handbook for Authors; Brogan, M. , Ed.; ACS: Washington, D.C., 1978.

[45] Dickens, C. Tale of Two Cities; Chapman & Hall: London, 1859; p 1.

[46] The Power and Promise of Early Research; Murray, D. H. ; Obare, S. O. ; Hageman, J. H. , Eds.; ACS Symposium Series; American Chemical Society, 2016; Vol. 1231 10.1021/bk-2016-1231.

[47] Hernik, J. ; Jaworska, E. INTED2018 Proceedings; INTED, 2018.

[48] Perla A. A., Hollar S., Muzikar K., Liu J. M. (2023). Using CREATE and
scientific literature to teach chemistry. J.
Chem. Educ..

[49] Reyes R. L. (2023). Exploring
science literature: Integrating chemistry research with Chemical education. J. Chem. Educ..

[50] Maxwell D. N., Spencer J. L., Teich E. A., Cooke M., Fromwiller B., Peterson N., Nicholas-Figueroa L., Shultz G. V., Pratt K. A. (2023). A Guided-Inquiry
Activity for Introducing Students to Figures from Primary Scientific
Literature. J. Chem. Educ..

[51] Gawalt E. S., Adams B. (2011). A chemical information
literacy program for first-year students. J.
Chem. Educ..

[52] Doble J., Karshbaum M., Wolf E., Singsaas E., Wainman J. W. (2024). Visible
Local Stakeholders in a Natural Resources Course-Based Undergraduate
Research Experience in General Chemistry II Laboratory. J. Chem. Educ..

[53] Blumling D. E., Hughey C. A., Boardman B. M., Judd O. H., Berndsen C. E., Boeckmann D. M., Paunovic D. M., Po T. M. (2022). Looking to Move
Away from Expository General Chemistry Laboratories? We May Have a
Cure for What “Ales” You. J. Chem.
Educ..

[54] Silsby C., McCormack R., Roll M. F., Moberly J. G., Waynant K. V. (2022). Implementing
the Elements of Course-Based Undergraduate Research Experiences (CUREs)
in a First-Year Undergraduate Chemistry Laboratory with Bioremediation
Relevance. J. Chem. Educ..

[55] Barr C. A., Brodeur R., Kumar U., Heilman D. W. (2022). Integrating authentic
research, peer learning, and high-impact project work into the general
chemistry laboratory. J. Chem. Educ..

[56] Talanquer V. (2013). Chemistry
education: Ten facets to shape us. J. Chem.
Educ..

[57] Nash L. K. (1976). Reflections
on freshman chemistry. J. Chem. Educ..

