# Supplementary material for: Primary Literature as the Course Design Centerpiece in a General Chemistry Course That Prepares Undergraduates for Research
Source: ACS Omega. 2025 Dec 8;10(50):61607–19. doi: 10.1021/acsomega.5c07586 (PMC12750229; doi:10.1021/acsomega.5c07586)
Supplement: Supplementary file 1 [file ao5c07586_si_001.pdf]

Primary Literature as the Course Design Centerpiece in a General Chemistry Course that Prepares Undergraduates for Research

Timm A. Knoerzer*, Kimberly A. Gardner, Mark D. Reimann, and Barry W. Hicks

United States Air Force Academy, Department of Chemistry, 2355 Fairchild Drive, Suite 2N-225, United States Air Force Academy, CO 80840, USA

*Corresponding author: timm.knoerzer@afacademy.af.edu

Supporting Information

Table of Contents of the Supporting Information:

Course syllabus	S2-S6
Unit learning objectives	S7-S8
Course schedule	S9-S16
US Air Force Academy example semester schedule	S17
Example chapter supplements	S18-S25
Literature pre-test	S26-S28
Article used for pre-test	S29-S31
Example final exam	S32-S48
First page of article used for final exam	S49
Example student lab report	S50-S57
Example of some good article choices (1st page only)	S58-S66
Example PowerPoint presentation	S67-S68
Literature summary examples	S69-S74

Chemistry 110S

Fall 2010

Course Syllabus

Course Goals

The following goals and methods of Chemistry 110S directly support the USAF Academy (USAFA) Mission and Outcomes.

> The USAF Academy's mission is *to educate, train, and inspire men and women to become officers of character, motivated to lead the United States Air Force in service to our nation*.

1) Knowledge: Develop a foundation of chemical knowledge and scientific common sense.

- Develop problem-solving tools common to all science and engineering
- Hands-on application of theory and processes through laboratories

2) Character and Discipline: Instill a sense of personal responsibility for learning and a desire to seek an understanding of processes in the world around us.

- Take responsibility for the success or failure of performance and gain self-confidence by performing new tasks in the lab and classroom.
- Perform under pressure while remaining honorable

3) Airmen-Scholars-Citizens: Inspire excellent officership and citizenship through instructor examples, an awareness of world issues, and relating chemistry to the Air Force.

- Lessons address Air Force applications, global problems, and ethical standards.
- Communicate and debate with a foundation of facts (*written lab reports, GR essays*)

4) USAFA Scholars Program: The Academy Scholars Program at USAFA helps academically talented cadets to reach their full potential by offering a unique and challenging path through the curriculum, thereby providing the Air Force and our Nation with a pool of intellectually talented and well-rounded leaders. The pedagogical principle of this enrichment program involves forming small learning communities (a cohort of cadets enrolled in the same sections) to provide close interaction among the same students over a four-year period, in courses pursuing a coherent theme – the development of the Western intellectual tradition. This close interaction, in pursuit of a liberal education, encourages a culture of academic excellence allowing each cadet to develop his or her full intellectual capacity.

- Lessons address Air Force applications, global problems, and ethical standards.

Expectations & Learning Plan of Attack

You are the primary person responsible for the quality of your education. Instructors, although helpful and motivational, play a secondary role in your education. The attitude and effort which you put into this course will be directly proportional to your learning and course performance.

Expectations of Students:

- Prepared for each lesson by doing the assigned homework (READING) and bringing *Napoleon's Buttons*, any relevant readings (electronic copies on your computer are acceptable), lab notebook, calculator, paper, and writing utensil to class.
- Actively pursue your learning by participating in class, seeking Extra Instruction when you are having problems, and mastering the homework.

Major Course Concepts:

1. Nomenclature – this term refers to the naming of chemicals or the jargon associated with scientific communication. We expect that you will develop the *language of chemistry* and be able to use vocabulary to execute precise communication.
2. Structure – most of the chemical materials in this course are compounds/molecules. As such, this term refers to the structure of the compound (being able to recognize, draw, and name). In addition, we expect that you will come to understand how structure relates to function of chemical compounds. Intermolecular forces, for example, will continually arise in our exploration of the 17 molecules that changed history.
3. Stoichiometry – this term refers to the measurement of amounts of chemical compounds. It is important to gain an appreciation of the role of "how much" in the course of chemical transformations.
4. Thermodynamics and Equilibrium – these concepts are important to understanding to what extent chemical compounds are transformed in the course of a reaction or redistributed during a physical change. For example, have you ever wondered why not all of the salt dissolves (immediately) when you add a tablespoon to a glass of water? Well, equilibrium plays a big part in such phenomena, and thermodynamic principles are intimately tied to it.
5. Kinetics – this refers to speed of reactions. Some are fast and some are slow, but the important thing to remember is that the speed of reaction (realm of kinetics) is generally independent of the extent of reaction (realm of thermodynamics).

NOTE: we will do our best to incorporate each of these themes into every unit. However, expect that some will take priority over others as we progress through the term.

Course Materials:

**Reading**:	*Napoleon's Buttons: 17 Molecules That Changed History*, Penny Le Couteur and Jay Burreson<br>Selected Excerpts from *Caveman Chemistry*, Kevin Dunn<br>Chapter Supplements to Relevant Content (Knoerzer and Hicks)<br>Selected journal articles from primary literature in chemistry
**Course Materials Address:**	**K:/DF/DFC/Chem 110S**
**Laboratory Notebook**:	Hardbound lab notebooks will be provided
**Lab manual (web based)**:	pdf file versions available on K:/ drive (password is view6box)
**Accessories**:	*Lab Safety Glasses* (optional for purchase but highly recommended – lab goggles are provided)

Course Policies

1) **Appointments:** Do not schedule appointments (dental, medical, tailor shop, etc.) during the first or second hour of a class or lab period. Attempt to reschedule appointments (made for you) that

conflict with class or lab periods.

2) **Bedrest & Hospitalization:** To miss class due to illness/injury, you must be put on bedrest by the cadet clinic or base hospital (notify your instructor by e-mail). If you are hospitalized and need extra instruction (EI), notify the hospital liaison officer to arrange EI with your instructor or call the Chem Dept (3-2960).
3) **Scheduled Absences:** As soon as you have a Scheduling Committee Action (SCA) code for missing an upcoming class, send this information to your instructor by e-mail (preferably at least two days in advance). Request instructor approval if required by the SCA (may be disapproved if you have a deficient grade in the course). For scheduled absences, complete online homework and Prelabs in advance; otherwise, you will receive zero points. Lab data sheets and written reports must be turned in no later than one lesson after an absence. For a missed GR, you will take the makeup exam during your first free period.
4) **Military Performance:** Instructors will provide input to your AOC based on your preparation for class, having the required materials, attendance, and any discipline problems. Unexcused lates or absences will be reported.
5) **Grading Guidelines:** The following are guidelines used to determine your grade:

> 80%	A- to A
70% - 80%	B- to B+
60% - 70%	C- to C+
50% - 60%	D
< 50%	F

If you do not earn at least 50% of Graded Review and Final Exam points, expect to fail the course. If you have a deficient (< 60%) overall grade, you are expected to remain in the classroom/ EI rm for the second hour of class (studying chem.)

6) **Graded Reviews (GRs):** Laboratory material will also be tested on GR's. GRs will be administered promptly at the start of the second hour of class for T1/T2 and the first hour of class for T3/T4. If you are unexcused late to a GR (<15 min), you will not be given extra time. If you are unexcused late (>15 min) or unexcused absent from a GR, you will take the GR during your first available free period with a 25% reduction in total assignment points.
7) **Homework:** **A)** Prepare and take notes for the assigned readings from *Caveman Chemistry* and *Napoleon's Buttons*. **B)** Read and review the instructor-written *Supplements* – prepare questions as needed. **C)** Read the primary literature (or other links provided in the supplementary material). **D)** Prepare your laboratory notebook. **E)** Perform research (electronic or hardcopy-written) as appropriate to understand what you are learning.
8) **Laboratory:** Your laboratory performance will be graded prelab preparation in lab notebook, final lab notebook, follow-up lab exercises, and lab reports. Lab notebooks must be properly prepared before the lab to include writing safety considerations and pertinent chemical data and reactions. Expect that many of the experiments that we are doing will overlap. It is imperative that you acclimate to multi-tasking and keeping your lab notebook (record of experimental work) organized. **We also expect that you will afford time at the end of day's work in the lab for clean-up!**
9) **Late Work:** Any assignment not turned in before the start of the class period (unless otherwise specified by your instructor) is considered late and worth zero points. In addition, an "incomplete" grade is recorded until you submit an acceptable assignment to your instructor.
10) **Collaboration Policy:** Collaboration with instructors and other cadets is allowed on labs, but it must be documented. Collaboration is an active cooperation between people to accomplish an

assignment. One-way discussion or sharing of completed work is not collaboration. Do not merely modify someone else's work, document this as "help", and hand the assignment in as your own work. This is considered copied work (not collaboration) and will be given zero credit.

11) **Documentation:** All submitted assignments require a **complete** documentation section: which problem?, references (who/what)?, how did the reference specifically help you?. If you do not include a documentation section, 10% of the assignment points will be deducted. Incomplete documentation will result in a loss of points at your instructor's discretion. Use an MLA Handbook for Writers to determine the proper format for documenting various references. If you did not use references or collaborate with others, put "none" in the documentation section. Handing in copied work with no documentation is unethical and dishonorable.

12) **Extra Instruction (EI):** Your primary resource for extra instruction is the EI Room. The EI room, 2N212, is open periods M1-M7 and T1-T6 (closed T6 on GR lessons). Group EI sessions cannot be scheduled during Academic Call to Quarters or within 12 hours of the M day GR. Group EI sessions will be posted under the course website announcements.

13) **Ethics Across The Curriculum (EATC):** Your instructor will periodically engage your class on ethical issues that are relevant to the Air Force and chemistry. Insights from your discussions will help you better understand the ethics of chemistry research and conclusions, which will aide your decision making process as an officer.

Chemistry 110S Graded Events

Overall Point Breakout

Class Participation (subjective)	150
Literature Pretest	100
3 Written Lab Reports (Practice-Level) @ 100 points	300
1 Lab report Revision @ 100 points)	100
1 Written Lab Report (Performance-Level)	300
4 Lab Exercises @ 50 points	200
2 Lab Notebook Checks @ 100 points	200
2 Graded Reviews @ 500 points each	1000
Cumulative Final Exam (Including Literature Final)	1000
Total Points	3350

Prog Point Breakout

1 Written Lab Reports (Practice-Level) @ 100 points each	100
Class Participation	50
Literature Pretest	100
4 Lab Exercises @ 50 points	200
1 Lab Notebook Check @ 100 points	100
1 Graded Reviews @ 500 points each	500
Total Points	1050

UNIT LEARING OBJECTIVES

UNIT TITLE	LEARNING OBJECTIVES (students will be able to……)
Metals and ceramics	• Make a crucible<br>• Use the crucible to produce a bronze alloy<br>• Perform appropriate stoichiometric calculations<br>• Describe the chemistry that occurs when the alloy forms<br>• Dissolve a copper mineral, determine its % of copper and use experimental data to identify the mineral<br>• Perform aqueous solution preparation and dilution strategies
Salt/peppers, nutmeg, and cloves	• Use standard chemical extraction method to separate a mixture of acid, base, and inorganic compounds<br>• Identify the structures of the components of the mixture<br>• Assess the purity of the isolated compounds<br>• Explain the process of chemical separation (including the important role of acid-base chemistry and ionization on aqueous vs organic solubility)<br>• Describe how chemical equilibrium plays a role in the extent of the reactions encountered in this unit
Morphine, nicotine, and caffeine/molecules of witchcraft	• Differentiate among extraction, crystallization and chromatography as separation strategies<br>• Describe the role of acid-bae chemistry in separation processes<br>• Describe the role of intermolecular forces in separation processes<br>• Describe the concept of chirality and differentiate among various stereoisomers and their properties
Ascorbic acid	• Identify and balance redox reactions<br>• Identify the elements oxidized/reduced as well as oxidizing/reducing agents<br>• Perform a redox titration and the associated stoichiometric calculations<br>• Describe the redox chemistry of ascorbic acid
Glucose and cellulose	• Draw and describe the features of carbohydrate structures<br>• Describe the differences between simple sugars and complex carbohydrates and their properties<br>• Describe the process of fermentation<br>• Perform analytical assessments of the products of fermentation (in this case mead)
Nitro compounds	• Describe the reaction involved in deploying a bottle rocket<br>• Perform stoichiometric calculations to prepare the bottle rocket fuel mixture<br>• Describe the kinetic and thermodynamic aspects of an exploding bottle rocket
Oleic acid	• Synthesize and determine the % yield of biodiesel<br>• Analyze the purity of biodiesel<br>• Describe the kinetics of biodiesel production (including the determination of kinetic order)<br>• Determine the heat of combustion for the burning of biodiesel

Soaps and dyes	• Synthesize a soap and be able to draw/describe its structure Synthesize a fragrance and be able to draw/describe its structure<br>• Synthesize a dye and be able to draw/describe its structure<br>• Explain the role of IMFs for these compounds<br>• Perform stoichiometric calculations<br>• Describe the equilibrium, kinetics and thermodynamics dimensions of these reactions
Phenol	• Describe the acid/base equilibrium aspects of phenolic compounds<br>• Describe the oxidation-reduction aspects of phenolic compounds<br>• Assess the antioxidant capacity of resveratrol
Wonder drugs/Chlorocarbon molecules	• Synthesize modafinil<br>• Perform requisite stoichiometric calculations to set up the reaction and determine yield<br>• Assess purity and use spectroscopic techniques to confirm structure<br>• Write a professional research report on experimental findings
Molecules vs malaria	• Extract quinine from cinchona bark<br>• Use spectrophotometric techniques to determine quinine concentration<br>• Demonstrate skill in making solutions and performing chemical analyses
Nylon vs silk	• Grow silkworms to produce silk (whole semester project)<br>• Isolate silk from the silk worms<br>• Synthesize nylon<br>• Describe the structure and properties of silk and nylon polymers<br>• Describe the reactions for the production of silk and nylon polymers

SCHEDULE

LESSON	TOPIC	HOMEWORK/ READING (before class)	LAB ASSIGNMENT
1<br>6 Aug<br>Fri	COURSE ADMINISTRATION Classroom Standards, Syllabus, Lab Safety, etc<br>1° LITERATURE PRE-TEST	Chapter 5 (Caveman Chemistry)<br><br>Supplement #1<br><br>Primary Lit. #1 267_CoalBased_JP900.pdf	Obtain Napoleon's Buttons and locate other course materials on the K:/ drive<br><br>Exp. #1 Make your crucible<br><br>Exp. #2 Dissolve copper mineral
2<br>10 Aug<br>Tue	METALS AND CERAMICS<br>INTRODUCTION TO READING PRIMARY LITERATURE	Chapter 5 (Caveman Chemistry)<br><br>Supplement #1<br><br>Primary Lit. #2 *Chem. Mater.* 2005, *17,* 1591-1596	Exp. #1 Constant mass of crucible<br><br>Exp. #2 Copper mineral spectrophotometric determination<br><br>Exp. #2 Sea shell demo (dissolve in HCl)
3<br>12 Aug<br>Thu	REVIEW LITERATURE PRETEST	Chapter 9 (Caveman Chemistry)<br><br>Supplement #1	Exp. #1 Constant mass of crucible -- Fire crucible<br><br>Exp. #2 Copper mineral spectrophotometric determination
4<br>16 Aug<br>Mon	METALS: SAMURAI VIDEO<br><br>SALT	Chapter 9 (Caveman Chemistry)<br><br>Supplement #1<br><br>Primary Lit. #3 *Inorg. Chem.*, 2004, 43, 5902-5907	Exp. #1 Make bronze<br><br>Exp. #2 Copper mineral spectrophotometric determination (LE #1)

5<br>18 Aug<br>Wed	SALT	Chapter 15 (Napoleon's Buttons)<br><br>Supplement #1<br><br>Primary Lit. #3 *Inorg. Chem.*, 2004, 43, 5902-5907	Exp. #3 Separation of organic base and carboxylic acid – TLC
6<br>20 Aug<br>Fri	SALT	Introduction (Napoleon's Buttons)<br><br>Supplement #2<br><br>Primary Lit. #3 *Inorg. Chem.*, 2004, 43, 5902-5907	Exp. #1 Weigh bronze/calculate % yield<br><br>Exp. #3 Separation of organic base and carboxylic acid – TLC
7<br>24 Aug<br>Tue	PEPPERS, NUTMEG, AND CLOVES	Chapters 1 (Napoleon's Buttons)<br><br>Supplement #3<br><br>Primary Lit. #4 *J Sci Food Agric* 79:1267±1274 (1999)	Exp. #3 Separation of organic base and carboxylic acid – TLC (LE #2)
8<br>26 Aug<br>Thu	PEPPERS, NUTMEG, AND CLOVES	Chapters 1 (Napoleon's Buttons)<br><br>Supplement #3<br><br>Primary Lit. #4 *J Sci Food Agric* 79:1267±1274 (1999)	Exp. #3 Separation of organic base and carboxylic acid – TLC (LE #2)
9<br>30 Aug<br>Mon	MORPHINE, NICOTINE, AND CAFFEINE	Chapters 13 (Napoleon's Buttons)<br><br>Supplement #4<br><br>Primary Lit. #5 *J. Agric. Food Chem.* 2007, *55,* 10022–10027	Exp. #4 Supercritical CO_2 extraction and/or isolation of eugenol from cloves

10<br>1 Sept<br>Wed	MORPHINE, NICOTINE, AND CAFFEINE	Chapters 13 (Napoleon's Buttons)<br><br>Supplement #4<br><br>Primary Lit. #5 *J. Agric. Food Chem.* 2007, *55,* 10022–10027	Exp. #4 Supercritical CO_2 extraction and/or isolation of eugenol from cloves<br><br>Exp. #6 Prepare for resolution (fractional crystallization)
11<br>3 Sept<br>Fri<br>*(Parents' weekend!)*	MOLECULES OF WITCHCRAFT	Chapter 12 (Napoleon's Buttons)<br><br>Supplement #4<br><br>Primary Lit. #6 *Chem. Res. Toxicol.* 2008, *21,* 2061–2064	Exp. #5 Caffeine from beverage (tea, coffee, espresso, analgesic) – TLC (LE #4)<br><br>Exp. #6 Check crystallization
12<br>8 Sept<br>Wed	MOLECULES OF WITCHCRAFT	Chapter 12 (Napoleon's Buttons)<br><br>Supplement #4<br><br>Primary Lit. #6 *Chem. Res. Toxicol.* 2008, *21,* 2061–2064	Exp. #4 Supercritical CO_2 quantify by GCMS(LE #3)<br><br>Exp. #5 Caffeine from beverage (tea, coffee, espresso, analgesic) – TLC (LE #4)
13<br>10 Sept<br>Fri	ASCORBIC ACID (DR. NORMAN HEIMER GUEST LECTURE)	Chapter 2 (Napoleon's Buttons)<br><br>Supplement #5<br><br>Primary Lit. #7 Structure of oxidized form of ascorbic acid	Exp. #5 Caffeine from beverage (tea, coffee, espresso, analgesic) – TLC (LE #4)<br><br>ChemSketch Download and Practice
14<br>14 Sept<br>Tue	ASCORBIC ACID	Chapter 2 (Napoleon's Buttons)<br><br>Supplement #5<br><br>**Primary Lit. Questions on any papers covered to this point**	Exp. #6 Work-up of resolution of enantiomers of α-methylbenzylamine<br><br>Exp. #6 Optical rotation (Report #1)

15<br>17 Sept<br>Fri	ASCORBIC ACID	Chapter 2 (Napoleon's Buttons)<br><br>Supplement #5<br><br>**Primary Lit. Questions on any papers covered to this point**	Exp. #6 Work-up of resolution of enantiomers of α-methylbenzylamine<br><br>Exp. #6 Optical rotation (Report #1)
16<br>21 Sept<br>Tue	GR #1	Review resolution exp.	Exp. #9 Make mead (need to prepare early for fermentation)
17<br>23 Sept<br>Thu	LAB DAY		Exp. #7 Quantitative analysis of Vit. C by iodometric titration (LE #5)
18<br>27 Sept<br>Mon	GR1 REVIEW		Exp. #7 Quantitative analysis of Vit. C by iodometric titration (LE #5)<br><br>Collect lab notebooks (for caffeine lab and prog. NB scores) and Report #1
19<br>29 Sept<br>Wed	ASCORBIC ACID	Chapter 2 (Napoleon's Buttons)<br><br>Supplement #5<br><br>**Primary Lit. Questions on any papers covered to this point**	Exp. #7 Quantitative analysis of Vit. C by iodometric titration (LE #5)
20<br>1 Oct<br>Fri	GLUCOSE AND CELLLULOSE	Chapters 3 and 4 (Napoleon's Buttons)<br><br>Supplement #6<br><br>Primary Lit. #8 *Ind. Eng. Chem. Res.* **2006,** *45,* 6477-6482	Exp. #8 Biocidal ABU (cotton)

21<br>5 Oct<br>Tue	GLUCOSE AND CELLLULOSE	Chapters 3 and 4 (Napoleon's Buttons)<br><br>Supplement #6<br><br>Primary Lit. #8 *Ind. Eng. Chem. Res.* **2006,** *45,* 6477-6482	Exp. #8 Biocidal ABU (cotton)
22<br>8 Oct<br>Fri	Workshop – revising Report #1	Chapters 3 and 4 (Napoleon's Buttons)<br><br>Supplement #6<br><br>Primary Lit. #8 *Ind. Eng. Chem. Res.* **2006,** *45,* 6477-6482	Exp. #8 Biocidal ABU (cotton)
23<br>13 Oct<br>Wed	GLUCOSE AND CELLLULOSE	Chapters 3 and 4 (Napoleon's Buttons)<br><br>Supplement #6<br><br>Primary Lit. #9 *J. Agric. Food Chem.* 2008, *56,* 12037–12045	Exp. #8 Biocidal ABU(cotton) – test fabrics for biocidal activity<br><br>Exp. #9 Mead analysis
24<br>15 Oct<br>Fri	GLUCOSE AND CELLLULOSE	Chapters 3 and 4 (Napoleon's Buttons)<br><br>Supplement #6<br><br>Primary Lit. #9 *J. Agric. Food Chem.* 2008, *56,* 12037–12045	Exp. #8 Biocidal ABU(cotton) – count colonies (LE #6)<br><br>Exp. #9 Mead analysis
25<br>19 Oct<br>Tue	NITRO COMPOUNDS	Chapter 5 (Napoleon's Buttons)<br><br>Supplement #7<br><br>Primary Lit. #10 *Anal. Chem.* 2010, *82,* 4015–4019	Exp. #9 Mead analysis (finish up) (Report 2)<br><br>Just for fun: Make bottle rockets

26<br>21 Oct<br>Thu	NITRO COMPOUNDS	Chapter 5 (Napoleon's Buttons)<br><br>Supplement #7<br><br>Primary Lit. #10 *Anal. Chem.* 2010, *82,* 4015–4019	Exp. #9 Mead analysis (finish up) (Report 2)<br><br>Just for fun: Make bottle rockets
27<br>25 Oct<br>Mon	OLEIC ACID	Chapter 14 (Napoleon's Buttons)<br><br>Supplement #8<br><br>Primary Lit. #11 *Green Chem.*, **2006**, 8, 861–867	Exp. #10 Make biodiesel<br><br>Just for fun: Fire bottle rockets
28<br>28 Oct<br>Thu	OLEIC ACID	Chapter 14 (Napoleon's Buttons)<br><br>Supplement #8<br><br>Primary Lit. #12 *Energy & Fuels* 2008, *22,* 1358–1364 (FOR GR 2)	Exp. #10 Refine and Test biodiesel (density, viscosity, pH and the amount of free glycerin, cloud point, etc.) (Report #3)
29<br>1 Nov<br>Mon	GR 2		Exp. #10 Refine and Test biodiesel (density, viscosity, pH and the amount of free glycerin, cloud point, etc.) (Report #3)
30<br>3 Nov<br>Wed	SOAP AND DYES	Chapter 9 (Napoleon's Buttons)<br><br>Go over GCMS data<br><br>LAB DAY	Just for fun: Make ORANGE 2; make ESTER
31<br>8 Nov<br>Mon	SOAP AND DYES	Chapter 9 (Napoleon's Buttons)<br><br>Supplement #9<br><br>Primary Lit. #13 *Langmuir* **2003,** *19,* 2034-2038	Just for fun: Make ORANGE 2; make ESTER<br><br>Just for fun: Make SOAP

32<br>10 Nov<br>Wed	SOAP AND DYES	Chapter 9 (Napoleon's Buttons)<br><br>Supplement #9<br><br>Primary Lit. #13 *Langmuir* **2003,** *19,* 2034-2038	Just for fun: Make ORANGE 2; make ESTER<br><br>Just for fun: Make SOAP
33<br>15 Nov<br>Mon	PHENOL	Chapters 7 (Napoleon's Buttons)<br><br>Supplement #10<br><br>Primary Lit. #14 *J. Agric. Food Chem.* 2008, *56,* 1415-1422	Exp. #11 Resveratrol project
34<br>17 Nov<br>Wed	PHENOL	Chapters 7<br><br>Supplement #10<br><br>Primary Lit. #14 *J. Agric. Food Chem.* 2008, *56,* 1415-1422	Exp. #11 Resveratrol project (Report #4)
35<br>19 Nov<br>Fri	WONDER DRUGS	Chapter 10 (Napoleon's Buttons)<br><br>Supplement #11<br><br>Primary Lit. #15 *J. Med. Chem.* 2005, *48,* 2262-2265	Exp. #12 Synthesis of anti-narcoleptic drug
36<br>23 Nov<br>Tue	WONDER DRUGS	Chapter 10 (Napoleon's Buttons)<br><br>Supplement #11<br><br>LAB DAY with CHEM 343 students	Exp. #12 Characterization of anti-narcoleptic drug (CHN, IR, NMR, polarimetry)
Com's Challenge & Thanksgivin g Break			

37<br>1 Dec<br>Wed	WONDER DRUGS<br><br>CHLOROCARBON MOLECULES (DDT) AND MOLECULES VS. MALARIA	Chapter 10 (Napoleon's Buttons)<br><br>Supplement #11<br><br>Primary Lit. #15 *J. Med. Chem.* 2005, *48,* 2262-2265	Exp. #12 Characterization of anti-narcoleptic drug (CHN, IR, NMR, polarimetry) – interpretation of data (LE# 5)<br><br>LAB PRACTICAL – quinine from cinchona bark
38<br>3 Dec<br>Fri	CHLOROCARBON MOLECULES (DDT) AND MOLECULES VS. MALARIA	Chapters 16 and 17 (Napoleon's Buttons)<br><br>Supplement #12	LAB PRACTICAL – quinine from cinchona bark
39<br>7 Dec<br>Tue	CHLOROCARBON MOLECULES (DDT) AND MOLECULES VS. MALARIA (DR. LOWELL KING GUEST LECTURE)	Chapters 16 and 17 (Napoleon's Buttons)<br><br>Supplement #12	LAB PRACTICAL – quinine from cinchona bark
40<br>9 Dec<br>Thu	REVIEW/ COURSE CRITIQUES (20 min)<br><br>1° LITERATURE POST-TEST (20 MIN)	CLEAN LAB (15 min)	Exp. #13 Make nylon -- Test nylon vs. silk from silkworms (30 min)

FALL 2025 SEMESTER CALENDAR (Current Sep 3, 2025)

SUNDAY	MONDAY	TUESDAY	WEDNESDAY	THURSDAY	FRIDAY	SATURDAY
**AUGUST**						
					A3-17 1<br>GRADUATION<br>ONBOARDING	2<br>CADET WING RETURNS 1900<br>ONBOARDING
3	4<br>TRANSITION<br>ONBOARDING	5<br>TRANSITION<br>ONBOARDING	6<br>TRANSITION<br>ONBOARDING	M1 7	T1 8	9<br>Silver Training Weekend
10	M2 11	T2 12	M3 13	T3 14	M4 15	16
17	T4 18	M5 19	T5 20	M6 21	T6 22	23
24<br>31	M7 25	T7 26	M8 27	T8 28	M9 29<br>PARENTS' WEEKEND	30<br>BUCKNELL
**SEPTEMBER**						
	HOLIDAY 1<br>LABOR DAY<br>NO CLASSES	T9 2	M10 3	T10 4	M11 5<br>Modified SoC - Afternoon Sections Start 1 Hour Early	6<br>Silver Training Weekend
7	T11 8	M12 9	T12 10	M13 11	T13 12	13<br>UTAH STATE (AWAY)
14	M14 15	T14 16	M15 17	T15 18	M16 19<br>Modified SoC - Afternoon Sections Start 1 Hour Early	20<br>BOISE STATE<br>Silver Training Weekend
21	T16 22	M17 23	T17 24	M18 25	T18 26	27<br>HAWAII<br>Silver Training Weekend
28	M19 29	T19 30				
**OCTOBER**						
			M20 1	T20 2	M21 3	4<br>NAVY (AWAY)
5	T21 6	M22 7	T22 8	M23 FALL CULEX 9<br>BEGINS AFTER CLASSES<br>Modified SoC - Afternoon Sections Start 1 Hour Early	10<br>NO CLASSES<br>FALL CULEX	11<br>UNLV (AWAY)<br>FALL CULEX<br>Silver Training Weekend
12	HOLIDAY 13<br>COLUMBUS DAY<br>NO CLASSES	T23 14	M24 15	T24 DSAT 16	M25 DSAT 17<br>Modified SoC - Afternoon Sections Start 1 Hour Early	18<br>WYOMING<br>Silver Training Weekend
19	T25 DSAT 20	M26 DSAT 21	T26 22	M27 23	T27 24	25
26	M28 27	T28 28	M29 29	T29 30	M30 31<br>Modified SoC - Afternoon Sections Start 1 Hour Early	
**NOVEMBER**						
						1<br>ARMY<br>Silver Training Weekend
2	T30 3	M31 4	T31 5	M32 6	T32 7	8<br>SAN JOSE STATE (AWAY)
9	M33 10	HOLIDAY 11<br>VETERANS DAY<br>NO CLASSES	T33 12	M34 13	T34 14	15<br>UCONN (AWAY)
16	M35 17	T35 18	M36 19	T36 20	M37 21	22<br>NEW MEXICO<br>Silver Training Weekend
23<br>CADET WING 30<br>RETURNS 1900	T37 24	M38 25<br>THANKSGIVING BREAK<br>LMD	26<br>----------	HOLIDAY 27<br>THANKSGIVING	28<br>----------	29<br>COLORADO STATE (AWAY)------
**DECEMBER**						
	T38 1	M39 2	T39 3	M40 4	T40 5	6<br>DEAN'S<br>WEEKEND
7<br>DEAN'S<br>WEEKEND	8<br>FINALS<br>1 & 2 & 3	9<br>FINALS<br>4 & 5 & 6	10<br>FINALS<br>7 & 8 & 9	11<br>FINALS<br>10 & 11 & 12	12<br>FINALS<br>13 & 14	13
14<br>----------	15<br>----------	16<br>----------	17<br>----------	18<br>----------	19<br>----------	20<br>----------
21<br>----------	22<br>----------	23<br>------- WINTER -------	24<br>----------	25<br>------- BREAK -------	26<br>----------	27<br>----------

NOTE: M vs T days in the schedule above – alternating class schedule days

Chapter Supplement Example 1

Napoleon's Buttons Ch. 12 Molecules of Witchcraft and Ch. 13 Morphine, Nicotine, and Caffeine

"It is true that my discovery of LSD was a chance discovery, but it was the outcome of planned experiments and these experiments took place in the framework of systematic pharmaceutical, chemical research. It could better be described as serendipity."-**Albert Hoffman**

"If they took all the drugs, nicotine, alcohol and **caffeine** off the market for six days, they'd have to bring out the tanks to control you" – **Dick Gregory**

Structure and Nomenclature

These two chapters are primarily about compounds called **alkaloids.** An alkaloid is generally a natural product molecule (typically extracted from a plant and occasionally from animals) that has a nitrogen-containing functional inherent to its structure. The important thing to remember is that this nitrogen atom is **basic** – meaning that it can accept a hydrogen atom from an acid. In organic chemistry, we refer to these types of basic nitrogen functional groups as **amines**.

The primary structural theme for all amines is that it is a central nitrogen atom bonded to three other groups. The simplest amine structure would be ammonia in which the central nitrogen is attached to three hydrogen atoms.

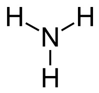

ammonia

Of course, there are a myriad of other possibilities. For example, imagine that all three hydrogen atoms are replaced with methyl (CH_3) groups. In this case, we now have trimethylamine. Trimethylamine is the common name for this compound and is likely the way a chemist would refer to it. You simply name each group attached to the nitrogen followed by "amine." If you wanted to name it according to the systematic nomenclature, it would be N,N-dimethylmethanamine.

common = trimethylamine

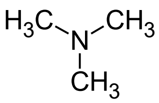

systematic = *N*,*N*-dimethylmethanamine

Here are a couple of more examples:

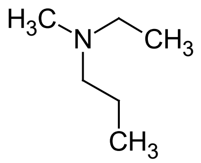

N-ethyl-*N*-methylpropan-1-amine

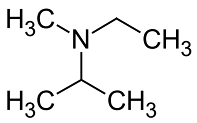

N-ethyl-*N*-methylpropan-2-amine

In this case, notice that all three groups are not the same. So, you name each group (aka substituent) in alphabetical order followed by "amine." This compound is ethyl methyl n-propylamine. Here the n-propyl is important to differentiate it from isopropyl – in essence where the three carbon chain attaches to the nitrogen atom. If you wanted to name this systematically, it would be N-ethyl-N-methylpropan-1-amine. Here the "1" is needed so you know that the amine nitrogen is attached to the number 1 carbon on the propyl group. The shorter groups are <u>substituents</u> and the longest is the <u>root name</u>, in this case, propanamine. Here are a few more examples. Can you name them both <u>commonly</u> and <u>systematically</u>?

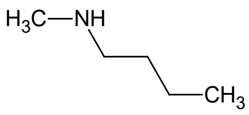

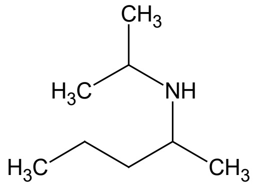

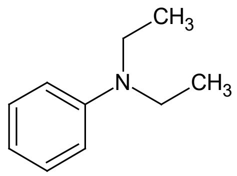

careful -- this one has an unusual name!

If you want more practice naming amines, try the following link:

http://www2.chemistry.msu.edu/faculty/reusch/VirtTxtJml/Questions/General/amine1.htm

The only other thing to realize about amines is that they come in five "flavors" – ammonia, primary, secondary, tertiary, and quaternary. Essentially, it depends upon <u>the number of attached hydrogens</u> (See the scheme below).

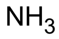

ammonia

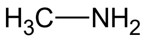

primary

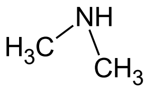

secondary

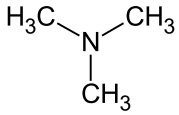

tertiary

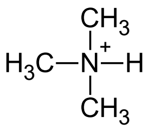

quaternary

Notice that in the first four cases, the central nitrogen has three bonds and one non-bonding electron pair (not actually shown above, but inferred). In the last structure, the central nitrogen has four bonding groups and zero non-bonding electron pairs – as a result the central nitrogen carries a +1 formal charge. This will be important as we introduce the acid-base principles below, so keep it in mind.

Now, this works well for relatively simple amine-containing compounds. However, for the alkaloids common names are preferred. Shown in the table below are some famous alkaloids with the basic amine functional group highlighted.

**Name**	**Alkaloid Structure**	**Primary, secondary, etc**
Methamphetamine	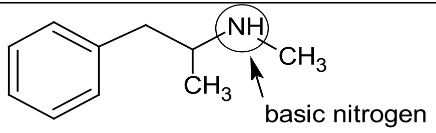	Secondary nitrogen (1 attached hydrogen atom)
Nicotine	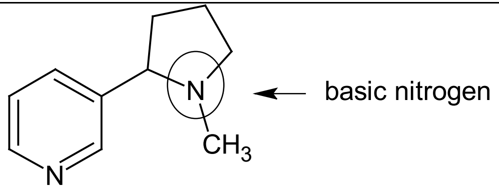	Tertiary nitrogen (no attached hydrogen atoms)
Caffeine	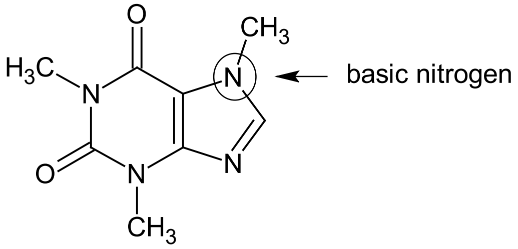	Tertiary nitrogen (no attached hydrogen atoms)
Morphine	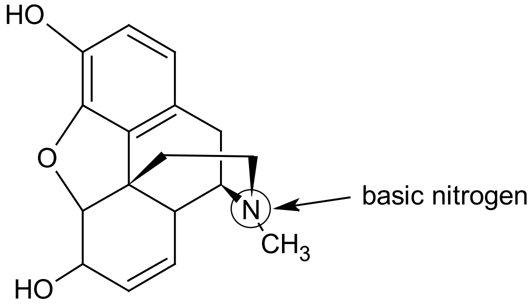	Tertiary nitrogen (no attached hydrogen atoms)
Cocaine	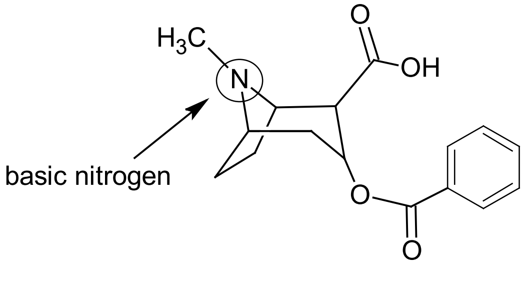	Tertiary nitrogen (no attached hydrogen atoms)

Coniine	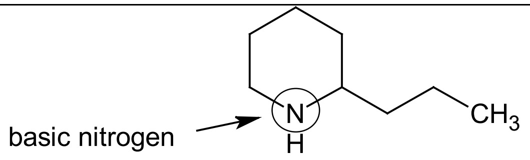	Secondary nitrogen (1 attached hydrogen atom)
LSD	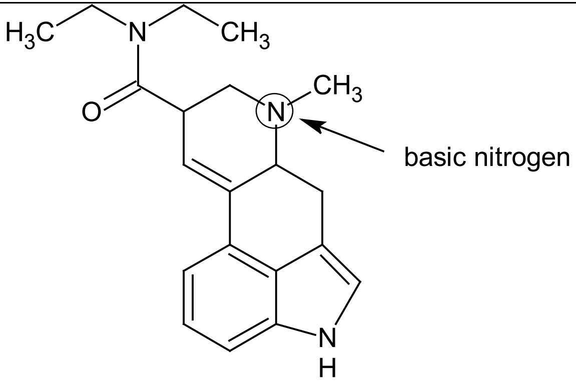	Tertiary nitrogen (no attached hydrogen atoms)

Please notice in the last structure (LSD) that this molecule also contains an **amide** group – you can recognize it because the nitrogen is directly attached to a carbonyl (C=O). Which other structure above has an amide group? What other functional groups do you see in the alkaloids listed in the table above? It is interesting that coniine contains ONLY the amine as the primary functional group whereas other molecules have functional groups in addition to the amine. That being said, it is the amine groups that is our focal point due to the fact that it is the functionality most responsible for the acid-base character of these types of compounds.

Reactions and Equilibrium

The term alkaloid implies that these compounds are BASES. As such, we expect them to accept hydrogen ions from acids. So, by definition we say that alkaloids are Bronsted-Lowry bases (H^+ acceptors). Let's look at a reaction.

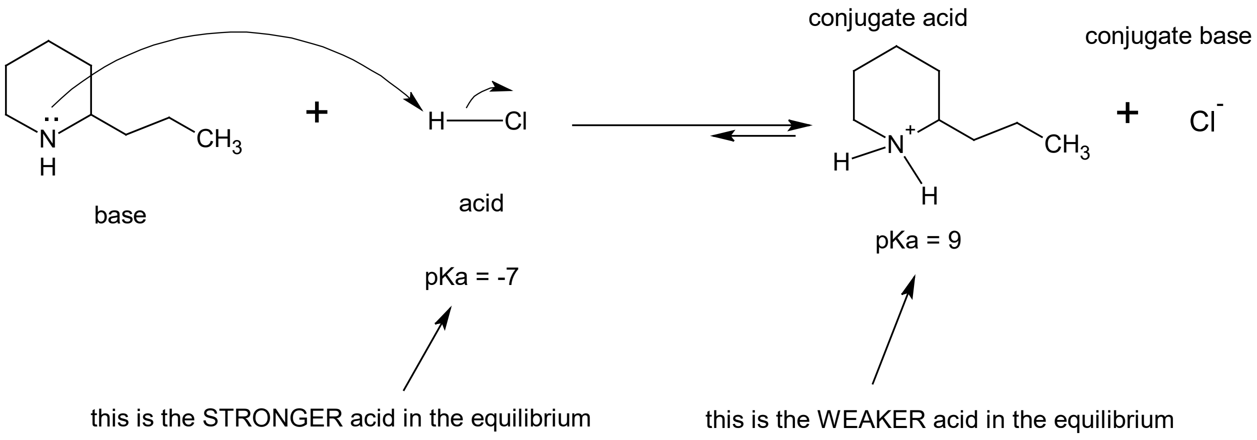

In the reaction above, the amine (coniine) is accepting a hydrogen from the strong acid HCl. So, coniine is the Bronsted-Lowry base and HCl is the Bronsted-Lowry acid in the forward direction. A fundamental question we can ask at this point is how far to the right or left does the reaction go? In other words, is the proton (H^+)

transfer from HCl to coniine complete or incomplete? This is an EQUILIBRIUM question. To determine the extent of this reaction, we refer to a quantity called the pKa value. The pKa value is a measure of the proton-donating capacity of an acid. For the forward reaction this is easy. The acid is HCl and you probably already know that it is a strong acid. But, what does that mean? It means that HCl will readily dissociate and give up/donate its hydrogen to a base. In terms of pKa what does this mean? The normal range of pKa values is roughly -10 to +60 pKa units. Something that has a pKa value of -10 would be a very STRONG acid; whereas something with a pKa value of +60 would be an incredibly WEAK acid. It ends up that HCl has a pKa value of -7. That would make HCl a strong acid. Let's look at it another way. The pKa value is the –log of the acid dissociation constant or Ka (pKa = -log Ka). So, if the pKa is -7, then the Ka (after you do the math) would be 1 x 10^7. Shown below is the dissociation of HCl:

$$H—Cl \rightleftharpoons H^+ + Cl^-$$

$$\text{where} \quad Ka = \frac{[H+]\,[Cl-]}{[HCl]}$$

So, in this case if Ka = 1x 10^7, then the concentration of products GREATLY EXCEED the concentration of reactants at equilibrium. Simply look at the reaction arrows – the forward reaction arrow is larger than the reverse reaction (re-association) indicating that at equilibrium HCl is essentially <u>completely dissociated</u> and that is exactly what we expect for a strong acid.

So, how does this apply to the reaction of coniine with HCl? Well, if HCl "completely" transfers its hydrogen to coniine, you should get the products shown above – the conjugate acid of coniine and the conjugate base of HCl. So, what happens at this point? Is it possible for the conjugate acid of coniine to transfer the hydrogen it just received back to the chloride anion to reproduce the reactants? In other words, can the reaction go backwards? The answer is yes, it CAN. But, will it? To answer that question you need to consider the pKa values for the two acids in your equilibrium. NOTE: acid-base reactions will have 2 acids and 2 bases (just look at the equation above). In the forward direction above, HCl is the Bronsted-Lowry acid with a pKa of -7 (clearly a strong acid). In the reverse direction, the conjugate acid of coniine is the Bronsted-Lowry acid with a pKa of 9 (a much weaker acid – by 16 pKa unit or a Ka differential of 1 x 10^{16}!!!!). That is a BIG difference. So, this reaction favors the formation of the products and AT EQUILIBRIUM, the major species in solution will be the conjugate acid of coniine and the conjugate base of HCl (the chloride ion).

Here's another reaction for you to consider. You need to determine if the reaction favors the products or the reactants at equilibrium and be able to support you decision based upon the relative pKa values of the 2 acids in the equilibrium.

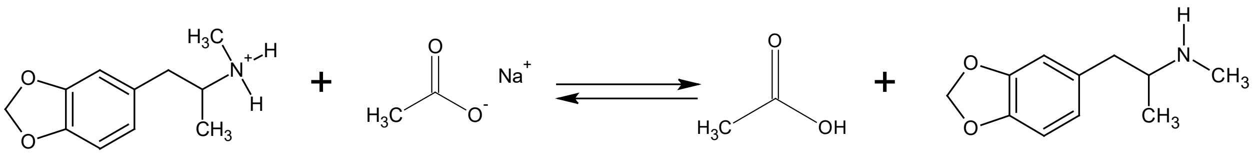

Below are a couple of links that may prove useful to you as you work this problem:

http://itech.pjc.edu/tgrow/2210/chm2210acidbase.pdf

http://research.cm.utexas.edu/nbauld/teach/acidsbases.html

Why is it important to know about these acid-base principles? There are a couple of reasons, but most importantly, we can take advantage of the fact that alkaloids (because they contain amine functionality) can be protonated under acidic conditions to give the nitrogen cation. In essence, free amines (neutrally charged; unionized) readily accept H^+ to form salts. In other words, the neutral free amine will have different properties than its ionized (salt) form. The free amine (aka free base) is soluble in organic solvents, usually polar organic solvents like ethyl acetate, diethyl ether, and dichloromethane. On the other hand, the ionized (cationic) form of the amine is a salt and is more water soluble. We can manipulate the amine to promote its solubility in organic or aqueous solvents depending upon whether or not it's the neutral amine or the ionized salt form. This process allows for effective separation of alkaloids (amines) via extraction into acidic aqueous solutions or into polar organic solvents under neutral or basic conditions. We will see this chemistry play out in the paper on the coniine enantiomers that we will read upcoming.

PRIMARY LITERATURE

f2010_paper6.pdf—Stereoselective Potencies and Relative Toxicities of Coniine Enantiomers – *Chem Res. Toxicol.* **2008,** *21,* 2061-2064.

PROJECT CHECKOFF REQUIREMENTS

1. Isolation of caffeine from different types of teas of your choice (LE #4) – this is will be a comparative study among cadets in your lab section and will be handed in separately at a date TBA.
2. Resolution of the enantiomers of α-methylbenzylamine and optical rotation (LE #5) – due date TBA

Chapter Supplement Example 2

Caveman Chemistry Chapter 10: Lime

This chapter isn't about fruit, it's about building materials. What do your teeth have in common with the floor? No, not contact, unless you're as clumsy as your training officer. Look down, you're standing (or sitting) on concrete (OK, it may be covered by tile or carpet or supporting your chair, but it's there!). Nearly every home in the US has a concrete foundation. The main foundation and structural components of every major office or business building are made from concrete. Most roads have concrete gutters and/or sidewalks, and many highways have a concrete foundation. Both teeth and concrete are made largely from calcium. Calcium is one of the more abundant elements in the earth's crust (see chapter 2) where it most often occurs as limestone (geologist-speak), or calcium carbonate (chemist-speak). The mining of calcium carbonate exceeds all other minerals combined with respect to total mass. If limestone is calcined (heated), lime or quicklime, is produced. Lime is the principal component of Portland cement, followed by silica (which we've already talked about). Cement is the main ingredient in concrete, and concrete is the most abundant building material in the world today. This is a bit surprising since modern day cement is often not as good as the binders made by the ancients.

You've already learned a bit about alkaline binders and the geopolymers we can make from them (chap 8); learn more by reading the review article (first read the paper below, then come back and read as much of this review as possible), "*Green chemistry for sustainable cement production and use*."

Nomenclature

Calcium carbonate, but copper(II) carbonate…why the difference? Calcium is in group II, and the only ion it forms (under normal conditions like aqueous solutions) is a +2 cation. Copper, and most other transition metals, form compounds with several different oxidation states. Thus, copper can exist in both +1 and +2 oxidation states, and it is necessary to indicate which one; copper(II) carbonate is $CuCO_3$, but one could also have copper(I) carbonate which would be Cu_2CO_3. To specify the difference, the oxidation state is put in Roman numerals in parenthesis just after the cation (and no space is left between the cation and the parenthesis). Iron exits in both +2 and +3 oxidation states, manganese with +4 and +7, chromium in +3 and +6, etc. Again, a quick glance at http://en.wikipedia.org/wiki/List_of_types_of_limestone shows why chemists have a systematic nomenclature, and why geologist need one.

Incidentally, the oxidation state is not necessarily the "charge on the ion," the oxidation number of Mn in the permanganate ion is +7, but there is no +7 cation present. It is a book keeping number for chemists that refers o the relative degree of oxidation. The rules for oxidation number assignments are mostly $O=2^-$, $H=1^+$, group I and II are +1 and +2, respectively, and halogens tend to be -1, and add them all up to get zero for neutral compounds or to the charge for ions, but I'll let you review that in your conventional text if you aren't familiar with this topic.

Reactions

You know that reacting carbonates with acid produces CO_2 and water. Heating carbonate salts likewise releases CO_2 and leaves a metal oxide behind as shown below. Likewise, heating hydroxide salts tends to dehydrate the salt leaving behind the metal oxide. Since many ores are carbonates or hydroxides, one of the first things that happens to them upon heating is conversion to the oxide (which you know can be reduced by smelting with carbon from last chapter):

$$MCO_{3\,(s)} \longrightarrow MO_{(s)} + CO_{2\,(g)}$$

$$M(OH)_{2\,(s)} \longrightarrow MO_{(s)} + H_2O_{(g)}$$

Stoichiometry

How much does human activity affect atmospheric CO_2, and how much is this tied to global warming? The scientific community has weighed in; human activities like burning petroleum for transportation and heat and making cement from limestone ***are*** contributing to the elevated CO_2 levels, and this ***has*** contributed to global warming. This does not mean we all have to grow out our hair, smoke reefers, join Greenpeace, follow the Grateful Dead, and attend government protest rallies demanding that the US join the Kyoto accord. The ***relative magnitude of human effects*** compared to the natural carbon cycle are not yet clear. But to deny that human activity is having an effect on world climate is to deny the scientific data; the only people still doing that are politicians…since politicians know much less about almost everything than almost anyone else, I'd listen to the scientists (also, be scared, be very scared when you find yourself agreeing with a politician on anything other than politics).

While it's true that natural sources of CO_2 emissions contribute far more CO_2 to the atmosphere than human activity (volcanoes produce large amounts of CO_2 by getting carbonates very hot -mostly calcium carbonate from subducted ocean floor), the natural sources tended to be in a "quasi equilibrium before our technological revolution. You can read more on the carbon cycle at: http://en.wikipedia.org/wiki/Carbon_cycle and on greenhouse gases at: http://en.wikipedia.org/wiki/Greenhouse_gas . A picture of the serious nature of this problem can be seen on the right. Incidentally, even if you do drive a Prius and abound with smug, you'll still be part of the CO_2 problem, because making cement produces huge amounts of CO_2 and we use a great deal of cement in making roads. Read more about cement chemistry at: http://en.wikipedia.org/wiki/Portland_cement before reading the review, "*Green chemistry for sustainable cement production and use*."

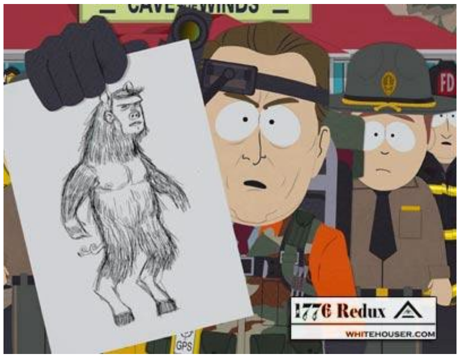

Structure

We like to think we are ingenious when it comes to architecture. Laughable. Although several plausible postulates have been made, we aren't even certain how the ancients made the pyramids. Some have even suggested they used a cement! Furthermore, our best ideas generally aren't as good as mother nature. Look at the structure of the siliceous sponge skeleton in the article, "*Skeleton of Euplectella sp.: Structural Hierarchy from the Nanoscale to the Macroscale*." What living nature can do that we cannot replicate in our materials, is create macroscopic materials that are ordered from the molecular level up-unlike uncontrollable precipitation reactions in most of our materials like metal alloys and concrete (see the micrograph of concrete at the right), which are aggregates without uniform structure from the nano scale up.

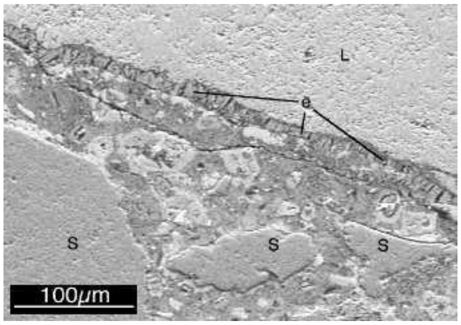

Thermodynamics

If you start with a carbonate ore like $CuCO_3$ and heat it, you get CuO and CO_2. The enthalpy change for this reaction is +47 kJ/mol (or twice that for two mols). If you take the CuO and heat it with graphite to make Cu and CO_2, the enthalpy change is -83 kJ/mol. Hess's law that says you can sum up reaction enthalpies when you sum the reactions and get the correct value as shown below:

$$2\,CuCO_{3\,(s)} \longrightarrow 2\,CuO_{(s)} + 2\,CO_{2\,(g)} \qquad \Delta H = +94\text{ kJ}$$
$$\underline{2\,CuO_{(s)} + C_{(s)} \longrightarrow 2\,Cu_{(s)} + CO_{2\,(g)} \qquad \Delta H = -83\text{ kJ}}$$
$$2\,CuCO_{3\,(s)} + C_{(s)} \longrightarrow 2\,Cu_{(s)} + 3\,CO_{2\,(g)} \qquad \Delta H = +11\text{ kJ}$$

In other words, it takes energy to make metals from ores, and metals will return to the more stable ores eventually (ever see rust? Its called iron(III) oxide by chemists and hematite by geologists), but hopefully you knew that.

Kinetics

Making lime or plaster-of-paris from limestone or gympsum requires heating. According to kinetic molecular theory, heat causes atoms, molecules or ions to move faster. The faster movement causes more violent collisions, some of which now have sufficient energy to break reactant bonds and initiate product formation. Thus, increasing the temperature of a reaction accelerates it by overcoming the activation energy barrier with faster molecules.

PRIMARY LITERATURE

1. Phair, J.W. **Green chemistry for sustainable cement production and use.** (2006) *The Royal Society of Chemistry*, 763-780.
2. Aizenberg, J., Weaver, J.C., Thanawala, M.S., Sundar, V.C., Morse, D.E., Fratzl, P. **Skeleton of Euplectella sp.: Structural Hierarchy from the Nanoscale to the Macroscale.** (2005) *Science*, 275-8.

PROJECT CHECKOFF REQUIREMENTS

1. Do all of the calculations to show the % of organic material in a Tums tablet.

Chemistry 110S **Name** ______________________________

Primary Literature Pretest

Section ___________

1. (5 pts) Describe how a novice chemistry student (you) should go about finding out the most recent information about a specific topic of which they know very little about, ***and*** include any specific information sources in your answer. For example, suppose I asked you to talk about the potential methods that could be used to clean a large-scale oil spill like the one currently in the Gulf of Mexico. How would you proceed to answer that question (NOTE: I am NOT asking you to answer that question, it is merely an example)?

2. (5 pts) What is the difference between primary literature and other chemistry literature? Is your chemistry textbook an example of primary literature?

3. (5 pts) Writings in the primary literature (scientific papers) are BROADLY organized into major sections. What are the major sections (headings) within almost all scientific papers?

4. (5 pts) What kind of information might you find in each of the sections that you suggested in question #2? (This is referring to the FINE organization of information that appears in scientific papers.)

5. (25 pts) These questions are from the article entitled, "Progress toward coal-based JP900." Use that article to answer the following questions:
   a) (2) Where was the work performed that is described in this paper, and who are the people that did it?

   b) (2) Why is this information from part 5a important to know?

c) (2) Who is the intended audience for this paper? How can you tell?

d) (2) What is the purpose of the research described in this article?

e) (2) What is the intended GAP in the research that these particular authors are attempting to address? Where did you find that information?

f) (2) What section is missing in this paper? How would this impede your ability to utilize information from this paper?

g) (2) In the first paragraph, the authors state that “Hydroaromatic compounds in the fuel provide *in situ* stabilizers that help retard decomposition.” What exactly is a hydroaromatic compound and how does it act as a stabilizer?”

h) (2) Describe the test for determining the thermal stability of a jet fuel.

i) (2) Write a balanced reaction for the conversion of naphthalene into a hydroaromatic compound.

j) (3) According to **Figure 2**, even burning n-heptane can lead to NO_x formation. How is this possible given that n-heptane is C_7H_{14}? Why do the other fuels lead to more NO_x emissions than heptane?

k) (4) Here's a possible scenario that you might face as an officer in the Air Force. Imagine that you had a long flight to make and your particular aircraft is fitted with a small fuel tank. In this case, you are running the risk of not having enough fuel to reach your destination. Which fuel would you prefer JP-8 or JP-900? ***Justify your response*** by explaining which factors you considered in making your answer.

6. (10) These questions are from the background and introduction of the NASA Technical Memorandum entitled, "Alternate Fuels for Use in Commercial Aircraft," by Daggett, et al.

a) (2) What is the purpose of this technical memorandum?

b) (2) Why is there a need for alternate fuels for commercial aircraft?

c) (3) Looking at **Figure 6** in the introduction, what is one disadvantage of coal -based JP900 not mentioned in the first article by Schobert et al.?

d) (3) How does this paper diverge in terms of appearance, organization, and audience versus the first paper?

PROGRESS TOWARD COAL-BASED JP900

Harold H. Schobert, Mark W. Badger and Robert J. Santoro
The Energy Institute, The Pennsylvania State University,
C211 Coal Utilization Lab, University Park, PA. 16802.

Introduction

For the past ten years, researchers at Penn State have been involved in the development of a jet fuel with superior resistance to decomposition in the pyrolytic regime, i.e., at temperatures above 400°C. The target has been a fuel that is stable at 480°C or 900°F, hence the origin of the designation JP-900. Early in the program we showed that fuels made from coal, or that contain coal-derived components, could have exceptional pyrolytic stability (1). Hydroaromatic compounds in the fuel provide *in situ* stabilizers that help retard decomposition. Fully hydrogenated cyclic compounds, cycloalkanes such as decalin, have very high intrinsic stabilities. Both hydroaromatics and cycloalkanes can be produced in high yields from coal (2). In addition to the scientific reasons for including coal-derived components in a JP-900 formulation, the use of coal also helps lessen dependence on imported petroleum.

The JP-900 program at Penn State has three components: production, stabilization, and combustion. In the past year we have successfully produced multiple-drum quantities of a prototype coal-based JP-900 at pilot-plant scale. This paper concentrates on an update of the pilot-scale production of the first prototype JP-900 fuels and preliminary data on their stability and combustion.

Discussion

The focus of fuel production is to incorporate coal or coal-derived materials into existing oil refinery operations. Two processes are under investigation at Penn State. The more advanced of the two involves the blending of coal tar distillates with petroleum refinery streams. Coal tar distillates are the only coal-derived materials that are commercially available and incorporate the "signature" cyclic structures of the parent coal. They represent the best available feedstock for early large-scale production of coal-based JP-900.

Refined chemical oil (RCO) is a distillate derived from the refining of coal tar (a by-product of the metallurgical coke industry). It represents around 10% of the coal tar yield, and contains about 70% naphthalene and substituted naphthalenes. Upon hydroprocessing, these compounds are converted into tetralin—a hydroaromatic compound—and decalin—a cycloalkane. Tetralin and decalin are among the compounds with the highest resistance to thermal degradation in the jet fuel range (180-330 °C). In this work, RCO was blended at various ratios with light-cycle oil (LCO). LCO is derived from fluidized catalytic cracking of petroleum residua. The blends were severely hydrotreated to remove sulfur and nitrogen, and to produce hydroaromatic compounds. Some of the hydrotreated products subsequently underwent aromatic saturation to completely hydrogenate the aromatic compounds to cycloalkanes. The pilot-scale production was performed by PARC Technical Services, Harmaville, Pa. PARC's adiabatic hydrotreatment pilot unit was used for both the hydrotreating step and for the aromatic saturation step and has been reported elsewhere (3).

Research at Penn State was focused on evaluating the quality of the fuels. In addition to the standard tests such as flash point, cloud point, and calorific value, detailed chemical composition analysis, thermal stability testing and combustion studies intended to simulate actual gas turbine combustor conditions have been carried out. Some of the data on one prototype JP-900 are provided in Table 1, along with data on JP-8.

Table 1. Comparison of the properties of JP8 and a JP900 jet fuel

Property	JP8	JP900
H/C ratio	1.91	-
Boiling Range, °F	330-510	356-626
Freeze point,°F	-60	-75
Flash point, °F	127.0	155.0
Net heating value, BTU/lb	18550	18260
Energy density , MJ/L	34.99	41.14
Specific gravity @ 60 °F	0.81	0.97
Avg Composition, vol%		
Aromatics	18.0	25.0
Hydroaromatics and cycloalkanes	20.0	75.0
Paraffins	60.0	0.0
Olefins	2.0	0.0
Sulfur, ppm	490	<3

Thermal stability tests are performed in static reactors and flow reactors, in the presence or absence of air. Figure 1 shows a comparison of the thermal stability of conventional JP-8 and a prototype JP-900 fuel. The graph shows the level of carbon deposition in an externally heated tube with respect to distance along the tube. As the fuel entered the tube at room temperature it was gradually heated as it traveled down the tube, reaching 480 °C at the exit. The carbon deposits were the result of the thermal break down of the fuel under the high-temperature conditions. The results show that the prototype JP-900 fuel comprehensively outperforms the JP8 under these operating conditions.

In addition to investigating the thermal stability of prototype JP-900, combustion studies have also been undertaken to examine the combustion instability and pollutant emissions characteristics of these fuels. Combustion instability refers to the occurrence of undesirable pressure oscillations due the coupling of unsteady heat release and the acoustics modes of the combustion chamber. The occurrence of combustion instability is a function of both the operating conditions and the combustor geometry. For the present studies, a model combustor is employed that allows the

comparison of the instability behavior of coal-based fuels with conventional petroleum based fuels. Combustion studies also allow measurements of the pollutant such as carbon monoxide (CO) and nitric oxides (NO and NO_2). The objective of these emissions studies is to provide a comparative based between current conventional jet fuels and coal-based fuel produced under the present research program.

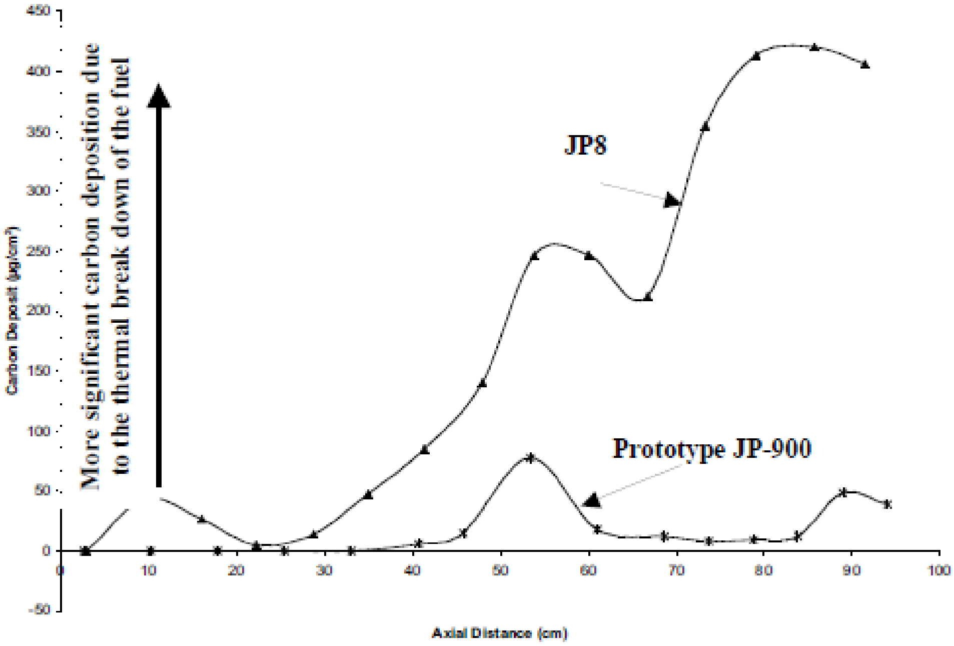

Run Conditions: Furnace temp. 700°C, bulk fuel outlet temp. 480°C, reactor pressure 700 psig, flow rate 10 ml/min, duration 7 hrs, tubing: 304 SS, 1/4" o.d., 1/8" i.d.

Figure 1. Comparison of the behavior of conventional JP-8 and Penn State's prototype JP-900 under stressing conditions at temperatures up to 900 °F .

The concept of lean-premixed combustion in gas turbine engine operation has become a standard in recent years as an effective means to meet stringent environmental standards on NO_x emissions. Similar trends in the aviation gas turbines to utilize lean direct injection techniques also result in pre-mixed conditions that lower emissions significantly. However, the understanding and control of pressure oscillations in the combustor when premixed fuel and air conditions occur represents one of the most challenging and least understood phenomena potentially limiting the development of future high performance gas turbine engines. In the present work, combustion experiments with the existing single injector gas turbine rig were performed to investigate combustion instability under high pressure and high temperature operating conditions. The fuels used for study were *n*-heptane, commercial kerosene (JP-8) and two blends of a coal-based liquid fuel. The coal-based fuels were hydrogenated blends of LCO and RCO, viz. hydrotreated (HDT) 1:1 blend of LCO/RCO and a 80/20 blend of saturated RCO and HDT RCO.

Several gas turbine combustion experiments were conducted on *n*-heptane, JP-8 and HDT-RCO/LCO coal-based fuels to study their effect on combustion instability and emissions. Parameters affecting combustion instability, such as combustion air inlet temperature, equivalence ratio and chamber pressure, were systematically changed to explore their effect on the unstable behavior of the combustion chamber.

Tests were conducted for 80/20 coal-based fuel with air inlet temperatures of 755 K (900°F) and 728 K (850°F). Comparison of the results shows that the air inlet temperature has a large effect on the pressure oscillations inside the combustion chamber. In particular, it was noted that for high air inlet temperatures, the chamber exhibited particularly

unstable behavior with pressure fluctuations of the order of 9% of the mean chamber pressure. At low air inlet temperatures, no instability was present and the pressure fluctuations never exceeded 2% of the mean chamber pressure. In addition, comparison of these results with those obtained for the HDT-RCO/LCO coal-based fuel shows that for the same air inlet temperature the 80/20 fuel is more stable than the 50/50 blend. In fact, while for the 50/50 blend pressure fluctuations of the order of 7% of the mean chamber pressure were recorded at equivalence ratios of the order of 0.5, pressure fluctuation for the 80/20 blend never exceeded 2% of the mean chamber pressure at 728 K (850°F) inlet conditions.

The stability map of 80/20 fuel was then compared to that of *n*-heptane and JP-8. For an air inlet temperature of 728 K (850°F), *n*-heptane exhibits the unstable behavior with pressure oscillations that exceed 10% of the mean chamber pressure, followed by JP-8, and the 80/20 fuel. The onset of combustion instability occurs for an equivalence ratio of the order of 0.65 for *n*-heptane and for a slightly lower equivalence ratio (0.60) for JP-8, whereas for the 80/20 fuel, the onset of combustion instability is not defined since the fuel has never shown unstable combustion behavior at 728 K (850°F) inlet conditions. When the air inlet temperature is increased from 728 K (850°F) to 755 K (900°F), the stability maps of *n*-heptane and the 80/20 fuel become comparable both in terms of maximum amplitude of pressure oscillations and in terms of the equivalence ratio for which the onset of instability occurs. Thus, the present studies indicate that coal-based jet fuels are potentially as good or superior to current petroleum-based jet fuels in terms of combustion instability behavior.

Along with stability maps, emissions for the four fuels were measured as a function of the equivalence ratio. For any equivalence ratio, HDT-RCO/LCO 50/50 combustion produced the highest NO concentration. Interestingly, the 80/20 fuel had the lowest NO concentration in the product gases. A different ordering of the fuels is observed for NO_x ($NO+NO_2$).

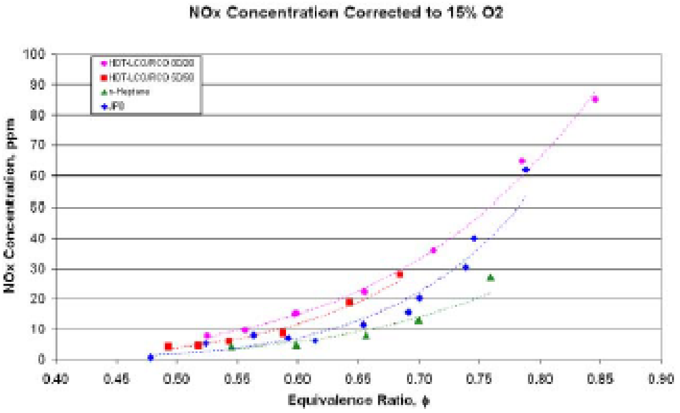

Figure 2. NO_x concentration in product gases versus equivalence ratio for various fuels.

In Figure 2, comparison of the NO_x emissions shows that the 80/20 fuel produced the highest concentration of this pollutant in the combustion gases, closely followed by HDT-RCO/LCO 50/50, JP-8 and *n*-heptane. In terms of CO emissions, HDT-RCO/LCO 50/50 produced the highest concentration for any given equivalence ratio, followed by JP-8, the 80/20 fuel and *n*-heptane. The observed differences in the NO and NO_x emission characteristics are interesting and will be further investigated in future studies. The fact that observed emission levels for NO_x were not significantly higher than for current jet fuels (e.g., JP-8), the achievement of comparable pollutant emissions using advanced gas turbine concepts appears feasible.

In current studies the evaluation of samples of JP-900 produced in a parametric study of the effects of LCO/RCO blend ratio, temperature, pressure, catalyst, and reactor space velocity. This evaluation will allow selecting the optimum conditions for a second pilot-plant production run. That pilot study will in turn lead to a planned refinery run that will make sufficient quantities of a second-generation prototype JP-900 for larger-scale combustion tests.

Acknowledgements

The authors would like to express their gratitude to the Department of Defense and Air Force Wright-Patterson Laboratory under contract F49620-99-1-0290 for the financial support of this project.

REFERENCES

1. Song, C., Eser, S., Schobert, H.H. and Hatcher, P.G., *Energy and Fuels*, **1993**, 7, 234-243.
2. Butnark, S, Badger, M.W., Schobert, H.H., *ACS Div. Of Fuel Chem.* **2001**, 46, (2), 492-494.
3. Butnark, S, Badger, M.W., Schobert, H.H., *ACS Div. Of Pet. Chem.* **2000**, 45, (3), 493-495.
4. Lefebvre, A. H., *ASME Journal of Engineering Gas Turbines Power*, **1995**, 11, 617-654.
5. Tacina, R. R., Wey, C. and Choi, K. J., *"Flame Tube NOX Emissions Using a Lean-Direct-Wall-Injection Combustor Concept"*, **2001**, AIAA-2001-3271.

"ACADEMIC TESTING MATERIAL"

ACADEMIC SECURITY**:** This examination is not released from academic security until 1650 on 16 Dec 2011 to coincide with the end of finals. Until this time, you may not discuss the examination contents or the course material with anyone other than your instructor. This exam time limit is 3.5 h

INTEGRITY: Your honor is extremely important. This academic policy is designed to help you succeed in meeting academic requirements while practicing the honorable behavior our country rightfully demands of its military. Do not compromise your integrity by violating academic security or by taking unfair advantage of your classmates.

AUTHORIZED RESOURCES: A calculator and a laptop computer to complete the SALG survey

WORK: To receive full credit for a problem, all calculations required to solve the problem must be shown. The inclusion of chemical structures, sketches, reactions, equations is HIGHLY encouraged to enhance the answer or provide clarification.

CHEMISTRY 110S NAME_________________________

Final

12 Dec 2011 SECTION______________________

1000 points

1. (190 points) ________________

2. (115 points) ________________

3. (385 points) ________________

4. (60 points) ________________

ACS Test (150 points) ________________

SALG Survey (100 points) ________________

* Go to http://www.salgsite.org/student * Fill in your email address * Enter the instrument number: 24975

TOTAL (1000 points) ________________

Chem 110S Final Exam
Fall 2011

1. (190 points) Shown below is a dose-response curve for 4 different drugs and their influence on production of acetylcholine.

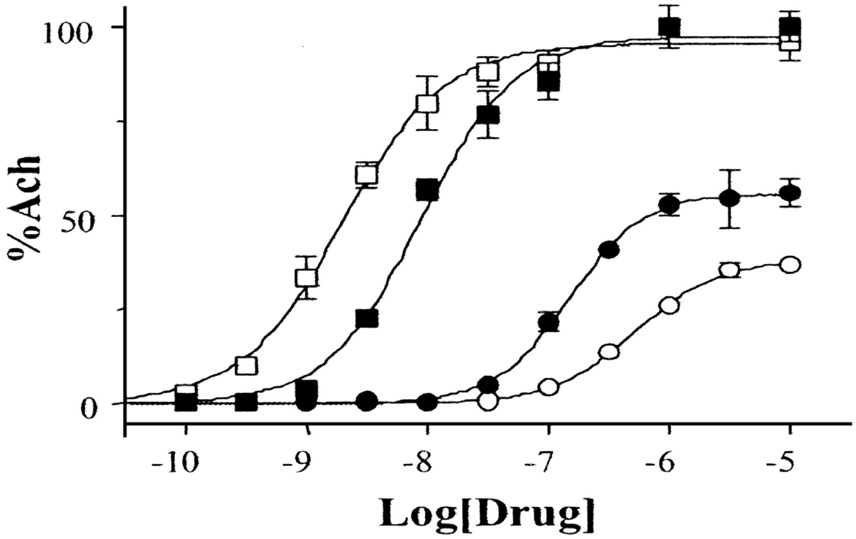

Drug 1 ■ Drug 2 □
Drug 3 ○ Drug 4 ●

(a) (10 pts) Which drug is the most potent? How do you know?

(b) (10 pts) Which drug(s) is/are the most efficacious (effective at eliciting the response)? How do you know?

(c) (10 pts) Assuming that these drugs act by binding to a biological receptor (protein), what does this information suggest about the **relative degree of intermolecular forces** among this particular set of drugs?

(d) Shown below are the structures for Drug 1 and the key amino acids in the binding site of the receptor.

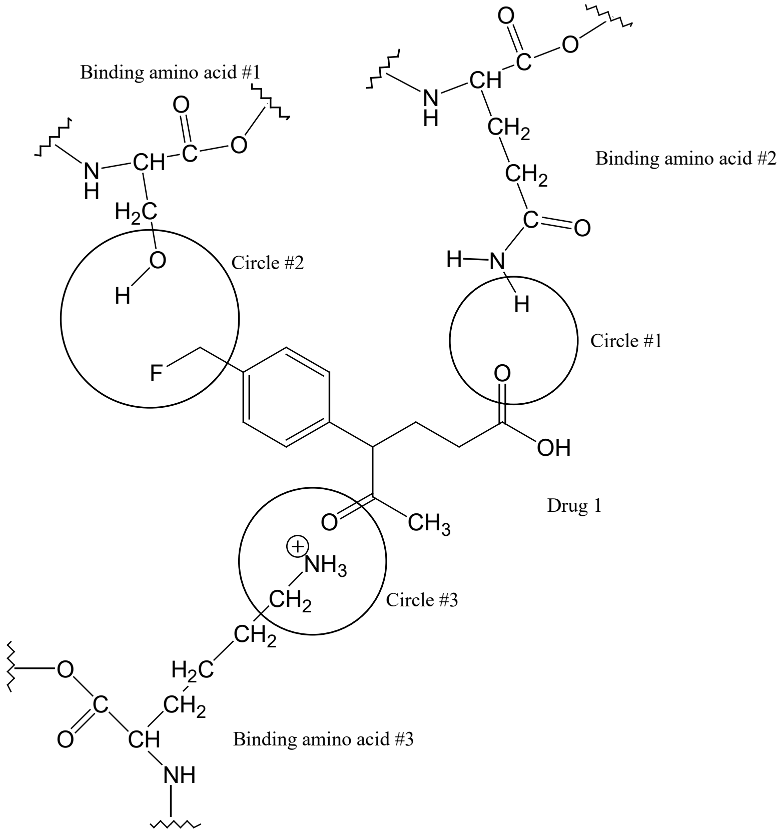

1. (5 pts) There is exactly one chiral atom in the drug molecule. Circle it.
2. (10 pts) Do you think there may be a difference in biological activity of Drug 1 depending upon whether you had the R or S enantiomer? Explain.

3. (10 pts) How could you use polarimetry to determine which enantiomer of the drug you might have?

4. (10 pts) Can this compound participate in London dispersion force interactions with the receptor? Why or why not?

5. (5 pts) Which circle (1, 2, or 3) is representative of a dipole-dipole interaction? ____

6. (5 pts) Which circle (1, 2, or 3) is representative of an ion-dipole interaction? ____

7. (5 pts) Which circle (1, 2, or 3) is representative of a hydrogen-bonding interaction? ____

(e) (10 pts) Binding of the drug to the receptor is an EQUILIBRIUM event. Explain.

(f) (10 pts) Write the equilibrium binding expression for the association of the drug with the receptor. HINT: it would be a good idea to think about a reaction expression here first.

(g) (10 pts) Which drug above would have the largest equilibrium binding constant? Why?

(h) (10 pts) How would you drive this association toward "completion" (i.e. what change would you suggest to provide more drug bound to the receptor?)

(i) (10 pts) If you were the person responsible for determining which drug to carry forward into further development for this particular pharmaceutical company, which one would you select? Why?

(j) (10 pts) How would your choice in part (i) change if you knew the following kinetic information relative to the metabolism and clearance of the drug?
Drug 1 $t_{1/2}$ = 8 hours
Drug 2 $t_{1/2}$ = 2 hours
Drug 3 $t_{1/2}$ = 10 hours
Drug 4 $t_{1/2}$ = 6 hours

(k) (10 pts) Drug 1 is partially soluble in water. Which of the following K_{sp} values describes its solubility? Circle your choice.

$K_{sp} = 3.45 \times 10^{-10}$

$K_{sp} = 2.77 \times 10^{-1}$

$K_{sp} = 8.32 \times 10^{8}$

(l) (10 pts) Drug 1 is weakly acidic with a Ka = 6.03×10^{-6} (pKa = 5.22). Assuming we can add enough water to make a solution of Drug 1 that is equal to 0.015 M, what is the pH of this solution? HINT: you'll need to first complete the acid dissociation reaction in water (below).

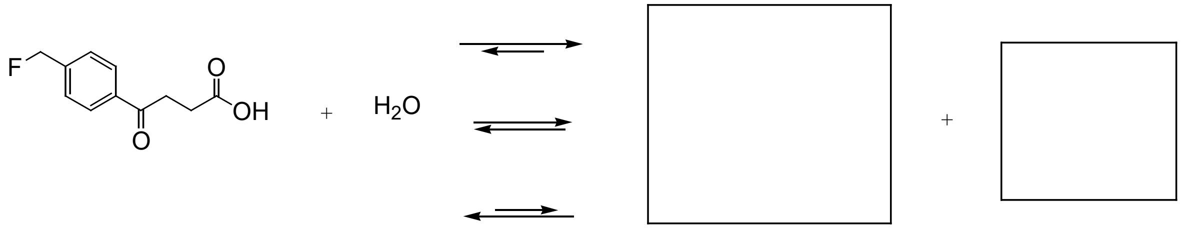

(m) (10 pts) Circle the CORRECT SET of equilibrium arrows in the reaction above.

(n) (20 pts) Imagine that we took our weakly acidic solution of Drug 1 and titrated it using 0.5 M NaOH. Sketch the titration curve and label the following key points on the curve:

1. Equivalence point
2. Midpoint of the buffer region

2. (115 points) The following are some general questions about quinine and its extraction from cinchona bark.

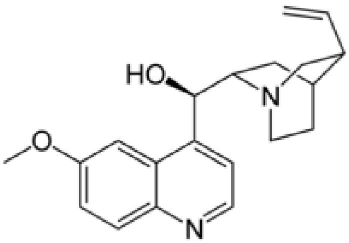

Quinine

a. (15 pts) Shown above is the structure for quinine (common anti-malarial medication). **Circle and identify** 3 organic functional groups present in the structure above.

b. (15 pts) To accomplish the extraction of quinine from cinchona, the bark is exposed to an acidic solution using pH 2-2.5 sodium citrate/citric acid buffer. Why would we expose cinchona bark to an acid? Write a complete and balanced chemical reaction that shows what happens to quinine upon exposure of the bark to acid. You can use H_3O^+ to represent the acid.

c. (20 pts) Suppose that we knew there was 350 mg of quinine (*free amine, not a salt*) in a massed sample of cinchona bark and we exposed the bark to 5 mL of a 0.01 M solution of the acid (as per the previous question)? Given these extraction conditions, we would fail to extract out all of the quinine. Show a calculation to support this idea. Now, provide a suggestion as to how we could adjust our experimental conditions to promote COMPLETE extraction of quinine from the bark.

d. (10 pts) Later on in the procedure, we can neutralize our aqueous (acidic) extract with NaOH (aq). Why do we do this? Write the complete and balanced chemical reaction that shows what happens at this stage of the experiment.

e. (10 pts) In the next stage of the process, we can expose our aqueous extract (basic) to 3 x 15 mL of CH_2Cl_2. Why do we do this?

f. (15 pts) How effective would this last step have been if we used ethyl acetate to extract instead of CH_2Cl_2? What if we used hexane instead? Would that be any better or worse than CH_2Cl_2? Integrate the concept of intermolecular forces into your answer.

g. (20 pts) This question deals with the quantification of quinine isolated from cinchona bark. Posted below are the experimental results for the standards and samples of quinine.

**Standard (uM)**	**Absorbance**		
75	285.24	Sample absorbance	147.077
50	235.69		
25	167.676		
10	106.736		
5	91.411		
1	71.884		

Assume that you obtain an extract with a mass of 327 mg (from the process in parts *b-f* above) dissolved in 25.00 mL of 0.05 M H_2SO_4. This sample solution was then diluted 1:100 to generate the final solution that was evaluated by fluorescence spectroscopy. Using the data above, generate a standard curve (<u>you can use your computer for this part ONLY – sketch you curve in the space provided</u>) and calculate the concentration (in M) of quinine in the ORIGINAL sample solution.

h. (10 pts) Given the value you determined in part (g), calculate the mass of quinine that you would expect to extract from 15.0 g of cinchona bark assuming that this quantity of bark yields 1.882 g of solid extract.

3. (385 points) This question pertains to the paper about synthetic jet fuel, *Highly efficient zirconium-catalyzed batch conversion of 1-butene: a new route to jet fuels* by Wright et al. **Energy & Fuels**, 22, 3299-3302 (2008).

a) (10 pts) Where was this work performed, and who funded this research? Why is this important to know?

b) (25 pts) <u>Credit for advanced preparation:</u>

If you contacted/emailed a principal investigator (author/researcher), give yourself 5 pts __________

If you ***obtained*** (you possess electronic copy or link) an additional FULL paper, give yourself 5 pts __________

If you ***examined*** an additional FULL paper, give yourself 5 pts __________

If you discussed this paper with (caveman) peer for >10 min, give yourself 5 pts __________

If you read an abstract for another paper, give yourself 5 pts __________

If you looked for information on the WWW, give yourself 5 pts __________

__________/25

c) (10 pts) Identify where the major sections can be found in this paper.

d) (10 pts) Summarize this article in laymen's terms in the space provided (~5 sentences).

e) (30 pts) Answer the following questions about chemical nomenclature used in this paper (put the name, formula or line drawing in as indicated):

1-butene (formula) ___________________________

H_2SO_4 (name) _____________________________

2-ethyl-1-hexene (formula) ________________________________

toluene (draw structure) ______________________________________

CaH_2 (name) _____________________________________

PtO_2 (name) ___________________________________

hexadecane (formula) _______________________________

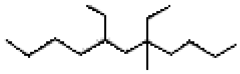

(name) ____

1,3-cyclopentadiene (line drawing) ______

f) (10 pts) What do the asterisks (*) refer to in the structures shown in Scheme 1 of the paper? Why is it important to know about this structural attribute for this particular study?

g) (10 pts) According to the abstract, "Quantitative conversion of 1-butene to a Schultz-Flory distribution of oligomers has been accomplished by use of Group 4 transition-metal catalysts in the presence of methylaluminoxane (MAO)." Which of the following would be examples of Group 4 transition metal catalysts? Circle your choice(s).

Iron
Molybdenum
Zirconium
Tungsten
Titanium

h) (10 pts) In the space provided to the right, sketch the chemical structure of the bis(cyclopentadienyl)zirconium dichloride catalyst used in this paper.

i) (10 pts) The paper states, "The new approach affords a product that is composed of 100% iso-paraffins, retains good fuel density, possesses attractive cold-flow properties, and, critical to Naval applications, can be easily tailored to have a high flash point." What is meant by **iso-paraffins**?

j) (10 pts) What is meant by "flash point," and why is it critical to naval operations to have a high value?

k) (10 pts) Write a balanced reaction for the production of 2-ethyl-1-hexene from 1-butene.

l) (30 pts) Given **Scheme 1** in the paper, determine which (net) reactant bonds are broken and which (net) product bonds are formed in converting:

	Reactant bonds broken	Product bonds formed
the monomer into the dimer		
the dimer into the trimer		
the trimer into the tetramer		

m) (45 pts) Given your answer to the above question, and the table of bond energies below, calculate the ΔH for each reaction, state whether the reaction is endo or exothermic, and predict the value of the corresponding reaction Keq.

Bond Lengths and Bond Energies

	Bond Length (nm)	Bond Energy (kJ/mol)
H–H	0.074	435
H–Cl	0.127	431
Cl–Cl	0.198	243
H–C	0.109	414
C–Cl	0.177	328
C–C	0.154	331
C=C	0.134	590
C≡C	0.120	812
C–O	0.143	326
C=O	0.120	803
C≡O	0.113	1075
N–N	0.145	159
N=N	0.125	473
N≡N	0.110	941

**REACTION**	**ΔH**	**ENDO OR EXO**	**Keq**<br>**=1**<br>**>1**<br>**<1**
the monomer into the dimer			
the dimer into the trimer			
the trimer into the tetramer			

n) (15 pts) Given your answer above, sketch reaction coordinate diagrams for each conversion: monomer to dimer solid line, dimer to trimer dotted line (- - -), dimer to trimer dashed line (….).

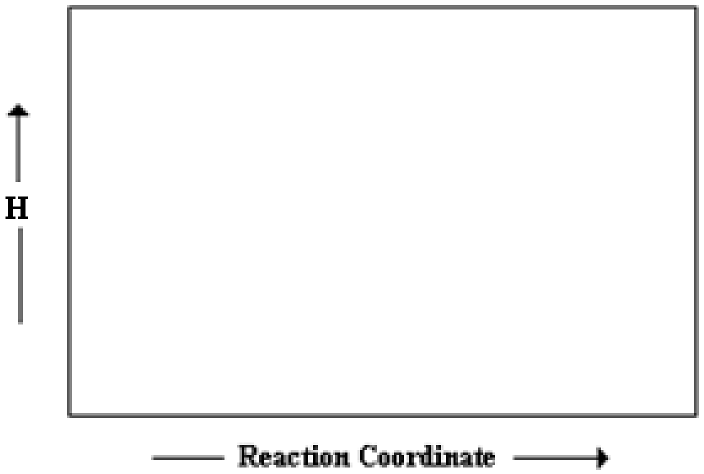

o) (10 pts) Assume the order of the reaction for each conversion above is first order with respect to 1-butene and first order with respect to the other alkene in each case. Write the rate law for ANY ONE (1) of the 3 conversions.

p) (10 pts) What would happen to the rate or reaction for any of the 3 conversions if you doubled the concentration of 1-butene?

q) (10 pts) Which of the following graphs would be linear? Circle your choice.

a. [1-butene] vs. time
b. 1/[1-butene] vs. time
c. Ln[1-butene] vs. time

r) (10 pts) Given your answers to the above questions (m-r), explain the "Schultz-Flory distribution" of products detected in Figure 1 (right). Do you think that **kinetic or thermodynamic factors** play the more important role in determining this distribution? Explain.

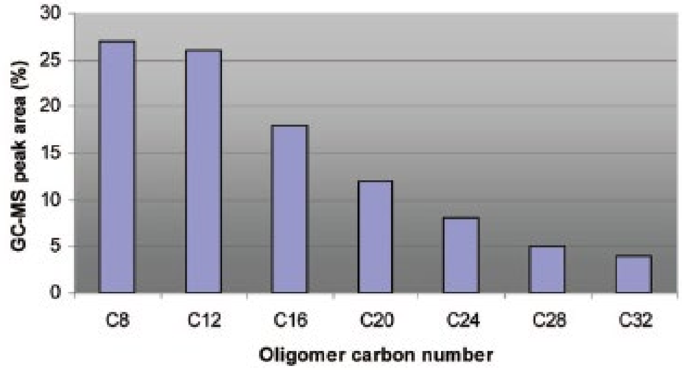

s) (10 pts) The authors employed GCMS as the key tool in quantifying and identifying the oligomers in this experiment. Briefly describe how this was done including a description of the technique itself.

t) (10 pts) Given Scheme 1 (right), draw the structure of the ***hexameric alk<u>e</u>ne*** product formed ($C_{24}H_{48}$), ***<u>and</u>***, how many stereoisomers of the **hydrogenated *alk<u>a</u>ne*** product from the hexamer will be present?

Scheme 1

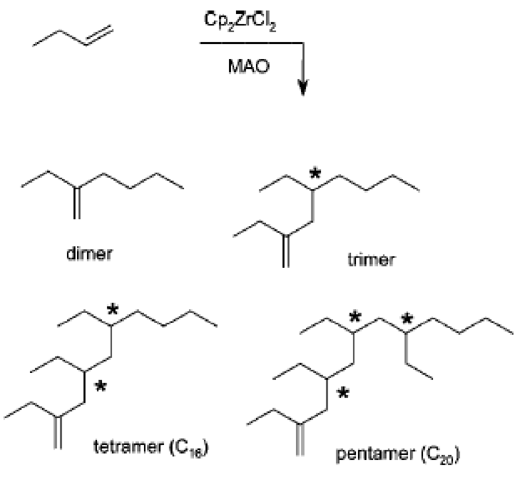

u) (10 pts) Presumably, 1-butene can form oligomeric alkenes in the *absence* of Cp_2ZrCl_2, albeit more slowly. In the space provided **sketch reaction energy diagrams** for the conversion of 1-butene into the dimer in the absence (solid) and presence (dashed) of Cp_2ZrCl_2.

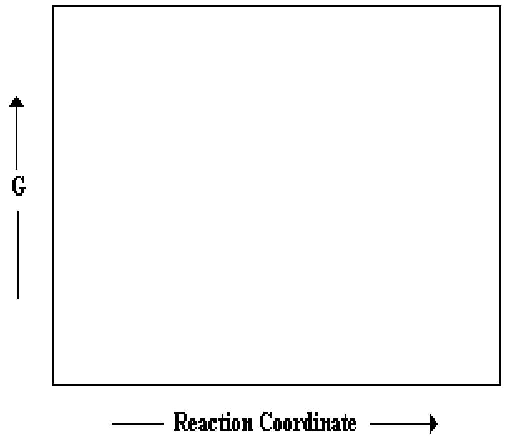

v) (10 pts) The authors state that the calculated heats of combustion are greater than 44+ MJ/kg. Write a balanced equation for the combustion of $C_{16}H_{34}$ (as per reference 22). How could you utilize this equation to verify that the heat of combustion is "greater than 44+ MJ/kg" (describe the method ONLY, don't perform the calculation)?

w) (10 pts) List 5 properties that must be considered in production of an acceptable jet fuel?

1.

2.

3.

4.

5.

x) (10 pts) The authors state that "For entry 1 in Table 1, elemental analysis indicates that we have 85% carbon and 15% hydrogen, which are consistent with a fully saturated hydrocarbon." Calculate the empirical formula from this information.

y) (10 pts) Calculate the mass % of each element in the Cp_2ZrCl_2 catalyst.

z) (10 pts) How could NMR be used to differentiate among the following compounds?

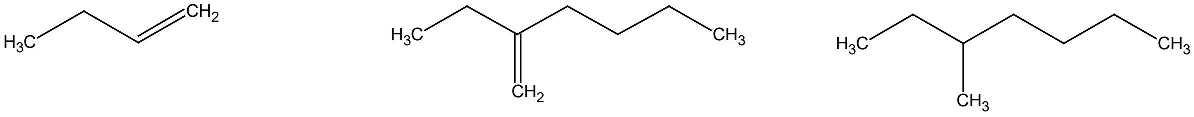

aa) (30 pts) Where is the US Air Force likely to get jet fuel in the next 10 years? Fifty years from now? Two hundred years from now? ***Justify your responses with 2 or 3 sentences for each response***.

10 years

50 years

200 years

4. (60 points) We have discussed many applications of chemistry topics and performed numerous lab experiments this semester. Understanding these activities has leveraged on 6 fundamental topics that were introduced and periodically revisited. **Using examples from the assigned primary literature and lab experiments**, describe in a short paragraph (3-4 sentences) or with diagrams/reactions how your understanding of each fundamental topic has been enhanced.

a. (10 pts) Chemical Nomenclature

b. (10 pts) Molecular Structure

c. (10 pts) Stoichiometry

d. (10 pts) Chemical Equilibrium (including reaction types: redox, solubility and acids/bases)

e. (10 pts) Thermodynamics

f. (10 pts) Kinetics

Highly Efficient Zirconium-Catalyzed Batch Conversion of 1-Butene: A New Route to Jet Fuels

Michael E. Wright,* Benjamin G. Harvey,* and Roxanne L. Quintana

United States Navy—Naval Air Systems Command (NAVAIR), Naval Air Warfare Center Weapons Division (NAWCWD), Research Department, Chemistry Division, China Lake, California 93555

Received May 21, 2008. Revised Manuscript Received June 20, 2008

Quantitative conversion of 1-butene to a Schultz–Flory distribution of oligomers has been accomplished by use of Group 4 transition-metal catalysts in the presence of methylaluminoxane (MAO). The oligomerization reaction was carried out at ambient temperature in a sealed reaction vessel with complete conversion of 1-butene at catalyst turnover numbers of >17 000. The combination of high catalyst activity without concomitant production of high polymer led to a highly efficient production of new hydrocarbon jet fuel candidates. The reaction proceeds with high regioselectivity; however, because achiral catalysts were used, several diastereoisomeric structures were produced and observed in the gas chromatography–mass spectrometry (GC–MS) chromatograms. The single and specific dimer formed in the reaction, 2-ethyl-1-hexene, was easily removed by distillation and then dimerized using acid catalysis to afford a mixture of mono-unsaturated C_{16} compounds. Changes in the oligomerization catalyst led to production of fuels with excellent cold-flow viscosity without the need for a high-temperature distillation. Thus, removal of the dimer followed by catalytic hydrogenation (PtO_2) led to a 100% saturated hydrocarbon fuel with a density of 0.78 g/mL, a viscosity of 12.5 cSt at −20 °C (ASTM 445), and a calculated heat of combustion of 44+ MJ/kg. By back-addition of hydrogenated dimer in varying amounts (6.6, 11.5, and 17 wt %), it was possible to tailor the viscosity of the fuel (8.5, 7, and 6.5 cSt, respectively).

There exist several commercial and research programs around the world aimed at creating full-performance jet fuels based on alternative feedstocks. Traditionally, jet propulsion (JP) fuels contain a complicated array of saturated and aromatic hydrocarbons that are highly refined to meet fuel specifications for a particular application. For instance, the Navy's JP-5 has a significantly higher flash point (60 °C) in comparison to the Air Force JP-8 and commercial jet fuel (~38 °C).[1] Syntroleum[2] and Sasol[3] have independently produced JP-5 and JP-8 equivalents based on gas-to-liquid (GTL) Fischer–Tropsch processes. One of the most challenging aspects to making a jet fuel using Fischer–Tropsch[4] chemistry has been to meet the required cold-flow properties. To date, this has required significant postprocessing or "reforming" of the fuel to increase the iso/normal paraffin product ratio.[5]

Conversion of propene and butylenes to dimers/oligomers was one of the first commercial processes in the petroleum industry.[6] Some more recent approaches have looked at using mesoporous catalysts[7] and newly designed large-pore acidic zeolite catalysts.[8] Transition-metal catalysts (homo- and heterogeneous), generally grouped into the category of Ziegler–Natta (ZN), have enjoyed a successful history for converting olefins, in particular, ethylene and propene, into oligomeric and polymeric materials.[9] Studies using 1-butene can involve a co-polymerization reaction with more reactive olefins, such as ethylene or propene.[10] A study by Kaminsky explored the oligomerization of 1-butene using selected chiral Group 4 transition-metal catalysts and methylaluminoxane (MAO).[11] In general, the catalysts studied required elevated reaction temperatures and typically led to incomplete conversion of the 1-butene. A study by Christoffers and Bergman reported that using an aluminum/zirconium ratio of 1/1 and with a nearly stoichiometric amount of zirconium "catalyst" that 1-butene could be converted selectively to dimer (2-ethyl-1-hexene).[12]

* To whom correspondence should be addressed. Fax: 760-939-1617. E-mail: michael.wright@navy.mil.

(1) Corporan, E.; DeWitt, M. J.; Belovich, V.; Pawlik, R.; Lynch, A. C.; Gord, J. R.; Meyer, T. R. *Energy Fuels* **2007**, *21*, 2615–2626, and references cited therein. Chang, P. H.; Colbert, J. E.; Hardy, D. R.; Leonard, J. T. *Prepr. Pap.—Am Chem. Soc., Div. Pet. Chem.* **2004**, *49*, 414.

(2) Feerks, R. L.; Muzzell, P. A. *Prepr. Pap.—Am Chem. Soc., Div. Pet. Chem* **2004**, *49*, 407–410. Muzzell, P. A.; Feerks, R. L.; Baltrus, J. P.; Link, D. D. *Prepr. Pap.—Am Chem. Soc., Div. Pet. Chem.* **2004**, *49*, 411–413.

(3) Lamprecht, D. *Energy Fuels* **2007**, *21*, 1448–1453.

(4) Fischer, F.; Tropsch, H. *Brennst. Chem.* **1923**, *4*, 276.

(5) Typically, the *Chevron isocracking technology* produces a predominance of methyl branching at the 2 position of a hydrocarbon chain; however, the chemical product distribution is quite complicated.

(6) Schmerling, L.; Ipatieff, V. N. *Adv. Catal.* **1950**, *21*, 2.

(7) Catani, R.; Mandreoli, M.; Rossini, S.; Vaccari, A. *Catal. Today* **2002**, *75*, 125–131.

(8) Schmidt, R.; Welch, M. B.; Randolph, B. B. *Energy Fuels* **2008**, *22* (2), 1148–1155, and references cited therein.

(9) Natta, G. *J. Polym. Sci.* **1955**, *16*, 143. Natta, G.; Pino, P.; Corradti, P.; Danusso, F.; Mantica, E.; Mazzanti, G.; Moraglio, G. *J. Am. Chem. Soc.* **1955**, *77*, 1708. Natta, G. *Angew. Chem.* **1956**, *12*, 393. Ziegler, K. *Angew. Chem.* **1952**, *64*, 323. Ziegler, K.; Holzkamp, E.; Breil, H.; Martin, H. *Angew. Chem.* **1955**, *67*, 541. For a recent review on metallocene catalysts for olefin, alkyne, and silane dimerization and oligomerization, see Janiak, C. *Coord. Chem. Rev.* **2006**, *250*, 66–94, and references cited therein. For a review on zirconium-based catalysts, see Belov, G. P. *Petrol. Chem.* **1994**, *34*, 105.

(10) Janiak, C.; Blank, F. *Macromol. Symp.* **2006**, *236*, 14–22, and references cited therein.

(11) Kaminsky, W. *Macromol. Symp.* **1995**, *89*, 203–219.

(12) Christoffers, J.; Bergman, R. G. *Inorg. Chim. Acta* **1998**, *270*, 20. For additional work on 1-butene dimerizations using late transition metal catalysts, see Small, B. L.; Schmidt, R. *Chem.—Eur. J.* **2004**, *10* (4), 1014–1020.

10.1021/ef800380b This article not subject to U.S. Copyright. Published 2008 by the American Chemical Society
Published on Web 07/29/2008

Confirmation of Racemic Modafinil Synthesis Method from Benzhydrol through NMR, GCMS, IR, and Elemental Analysis.

Chemistry 110S Laboratory, United States Air Force Academy, United States Air Force, Colorado Springs, CO 80841

The synthesis of racemic modafinil from benzhydrol is entailed. Modafinil, a compound known to help counter the effects of narcolepsy, was formulated using a short, four-step chemical process. The modafinil collected for this experiment was tested for purity using nuclear magnetic resonance spectroscopy (NMR), X-ray diffraction analysis, mass spectrometry (MS), and infrared spectroscopy (IR) in order to verify the effectiveness of the synthesis procedure.

Introduction

Narcolepsy is a sleep disorder that affects approximately 1 in every 2,000 people and its prevalence in adolescents is becoming rapidly apparent. (ref 1) The disorder is characterized by extreme fatigue at inappropriate times during the day, affecting performance at school and at work. This disorder results in abnormal sleeping patterns, a speedier advancement into REM sleep, and cataplexy (ref 2)

Fortunately, a variety of options have become available to help treat narcolepsy. The major classifications in which potential medications are produced include stimulants, selective serotonin or norepinephrine reuptake inhibitors, tricyclic antidepressants, and sodium oxybate. (ref 3) As narcolepsy is an issue of keeping the central nervous system active during normal daily activities, stimulants provide the most logical and common treatment for the disorder.

Of the many stimulants tested and available, the medications prescribed most frequently include Adderall, Ritalin, and Provigil. (ref 4) While each has its benefits depending on the severity and symptoms of each case, Provigil is typically prescribed initially as it is not as addictive as the other two stimulants. Provigil, scientifically referred to as modafinil also has both less common and less severe side effects compared to the other two stimulants.

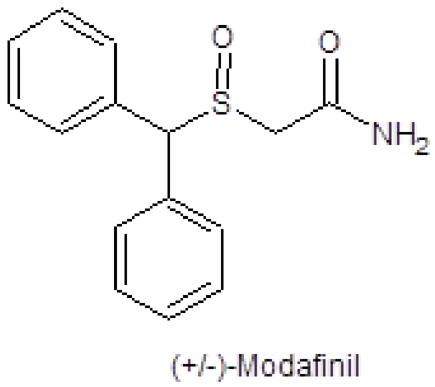

Figure 1. Modafinil Structure

Though the causes of narcolepsy have not been confirmed, narcolepsy appears to result from a reduced number of proteins called orexins which control appetite and sleeping patterns from the brain. (ref 3) These proteins project from the hypothalamus to various parts of the brain to perform these regulations, but they must be activated by monoamines such as dopamine and norepinephrine. (ref 3) Stimulants increase levels of these monoamines to activate the orexins to do their job in the brain. (ref 2) Specifically, modafinil concentrates on the dopamine transporter, and acts as a weak dopamine reuptake inhibitor with almost zero abuse potential. (ref 2)

The purpose of this study was to confirm the results of a previous study in which modafinil was resolved from benzhydrol through a short four step synthesis using chemical transformations. NMR and IR spectroscopy as well as TLC analysis were employed to verify the effectiveness of the synthesis and confirm the structural identity of the intermediates and the final structure. Measurements were also taken to determine the effectiveness of the synthesis through percent yield.

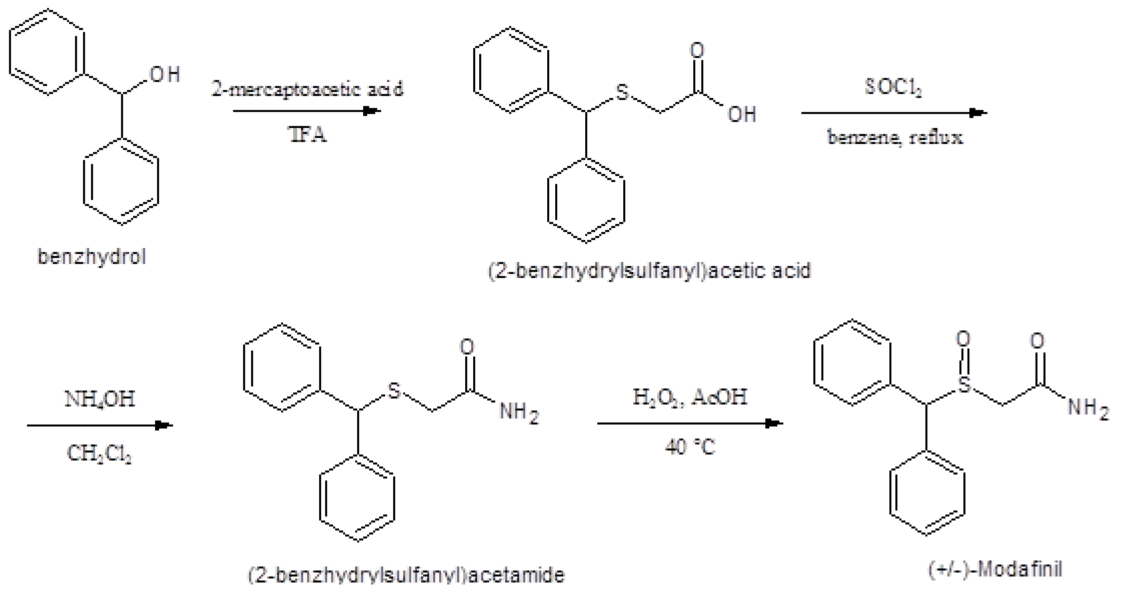

Methods

Convert benzhydrol to (2-benzhydrylsulfanyl)acetic acid – see method found in (ref 1) "Synthesis and determination of the absolute configuration of the enantiomers of modafinil."

Convert (2-benzhydrylsulfanyl)acetic acid into (2-benzhydrylsulfanyl)acetimide – see method found in (ref 1) "Synthesis and determination of the absolute configuration of the enantiomers of modafinil."

To a 10mL Erlenmeyer flask, .776 mmol of 2-(diphenylmethylthio) acetamide, .916 mmol of 30 percent hydrogen peroxide, and 1mL of acetic acid were added. The mixture was stirred while heating in a water bath of 40 degrees Centigrade. The progress of the reaction was observed by TLC at 20-minute intervals. When the reaction reached completion, the mixture was cooled to room temperature before water was added to form a white precipitate. The mixture was then cooled further in an ice bath before the precipitate was collected by vacuum filtration. Aqueous methanol and drying of the mixture were used to recrystallize the product.

The final product was then analyzed through H and C NMR, IR spectroscopy, GCMS, and CHNS.

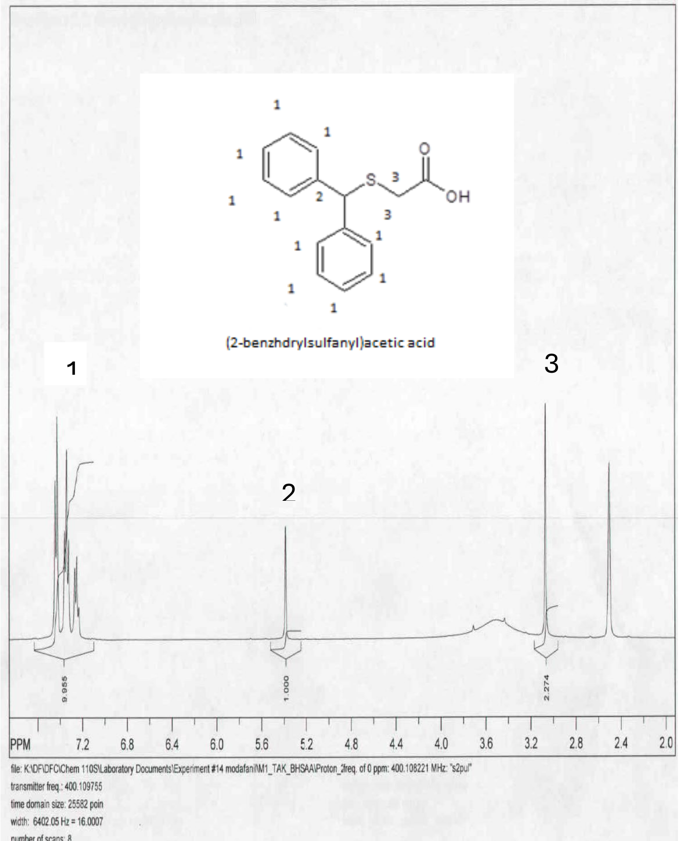

Figure 3. H NMR of (2-benzhydrylsulfanyl)acetic acid

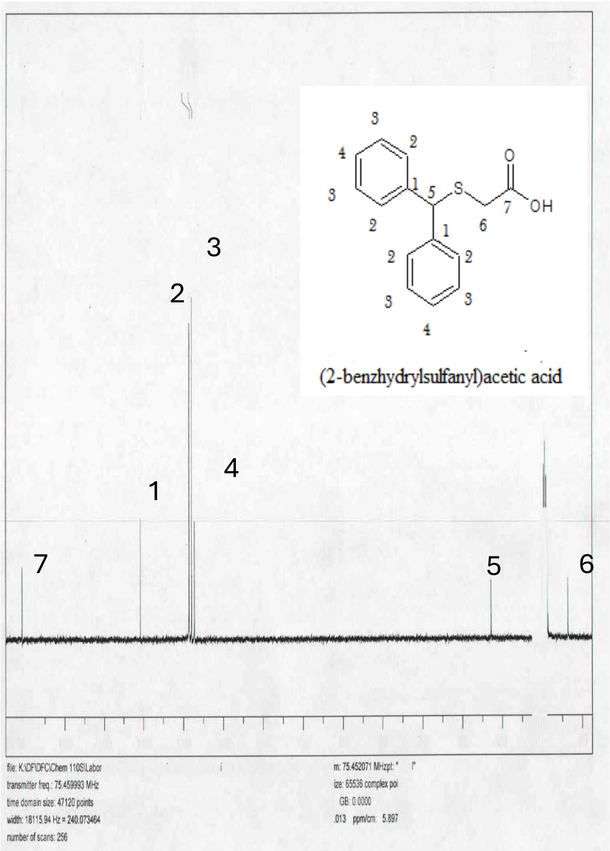

Figure 4. C NMR of (2-benzhydrylsulfanyl)acetic

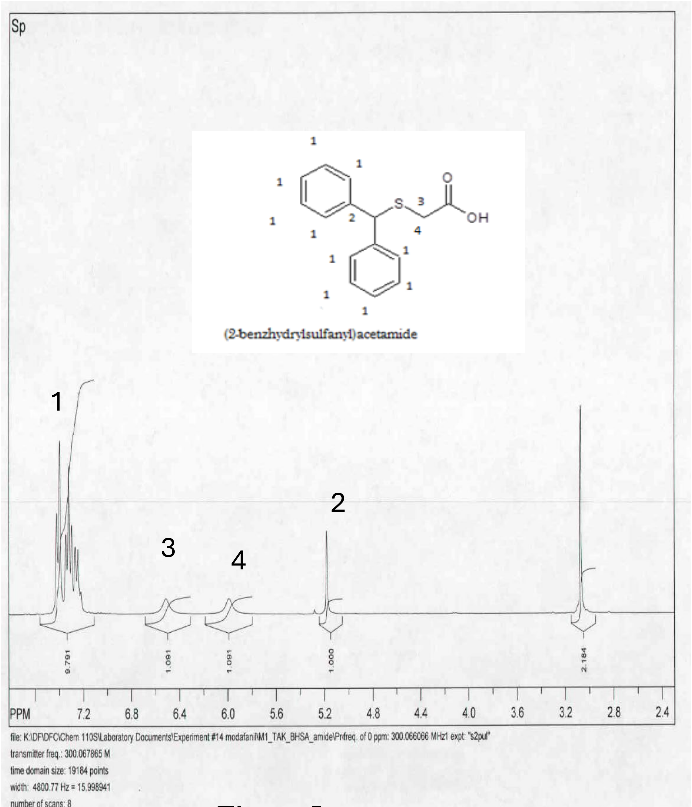

Figure 5. H NMR of (2-benzhydrylsulfanyl)acetimide

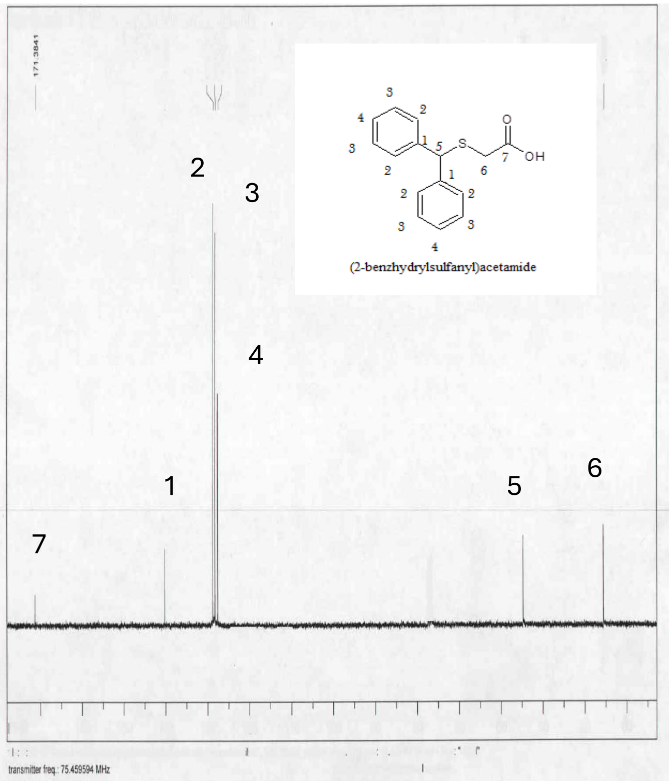

Figure 6. C NMR of 2-benzhydrylsulfanyl)acetimide

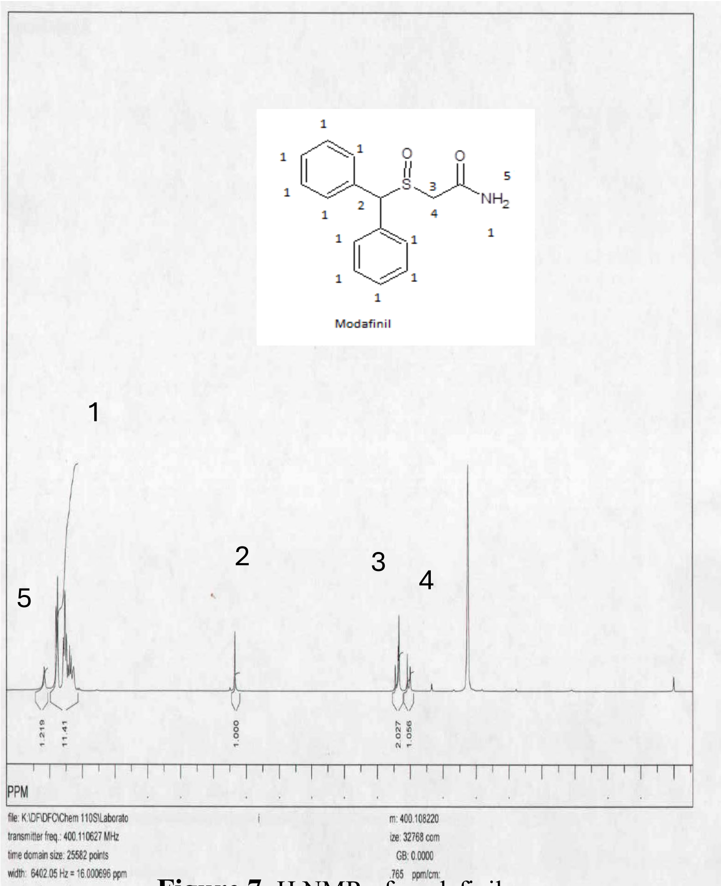

Figure 7. H NMR of modafinil

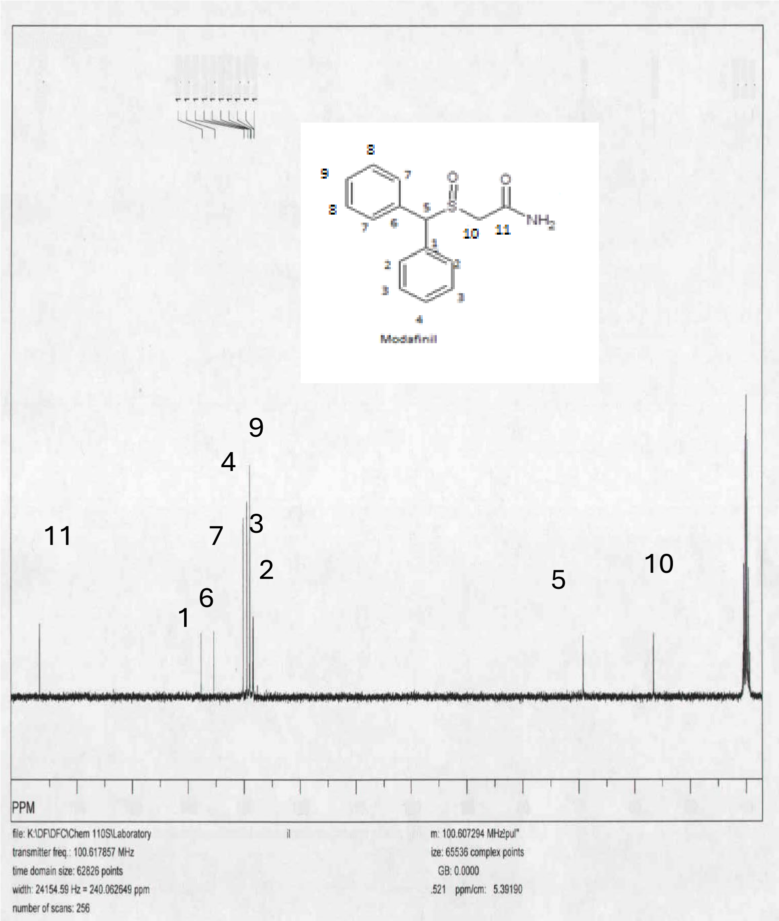

Figure 8. C NMR of modafinil

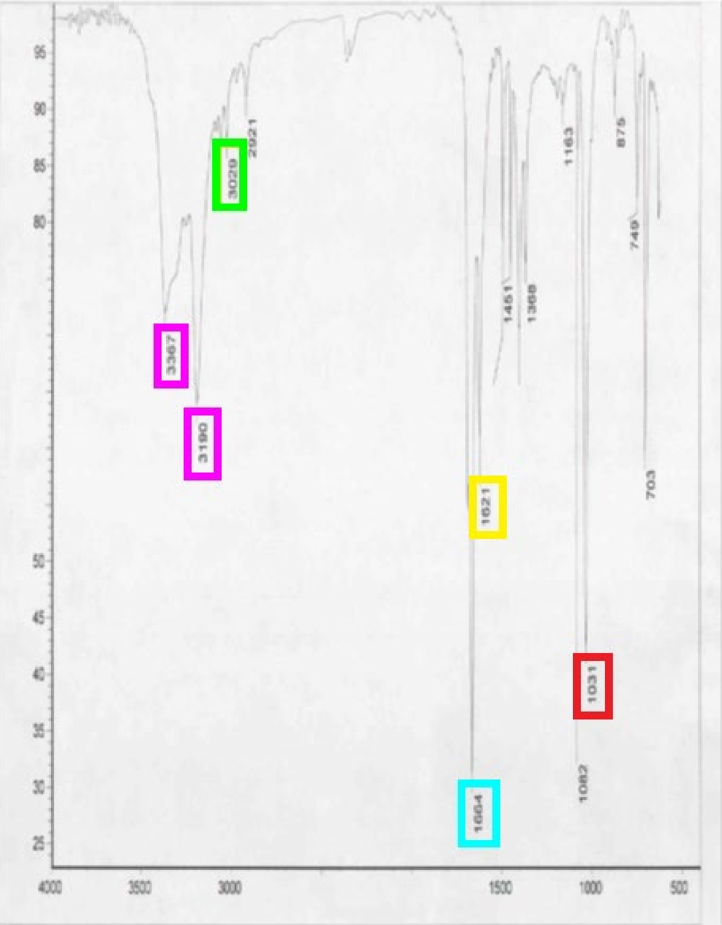

Figure 9. IR spectrum for modafinil

Table 1. Bonds present in modafinil and their expected location in an IR spectrum

	IR number	Intensity
Sulfoxide	1030 - 1060	Strong
C – H in Arene Benzene ring	3030	Variable
Carbonyl amide	1630 – 1695	Strong
NH2 amine	3300 – 3400	Weak
C = C in Arene Benzene ring	1600 & 1500	Medium/Weak

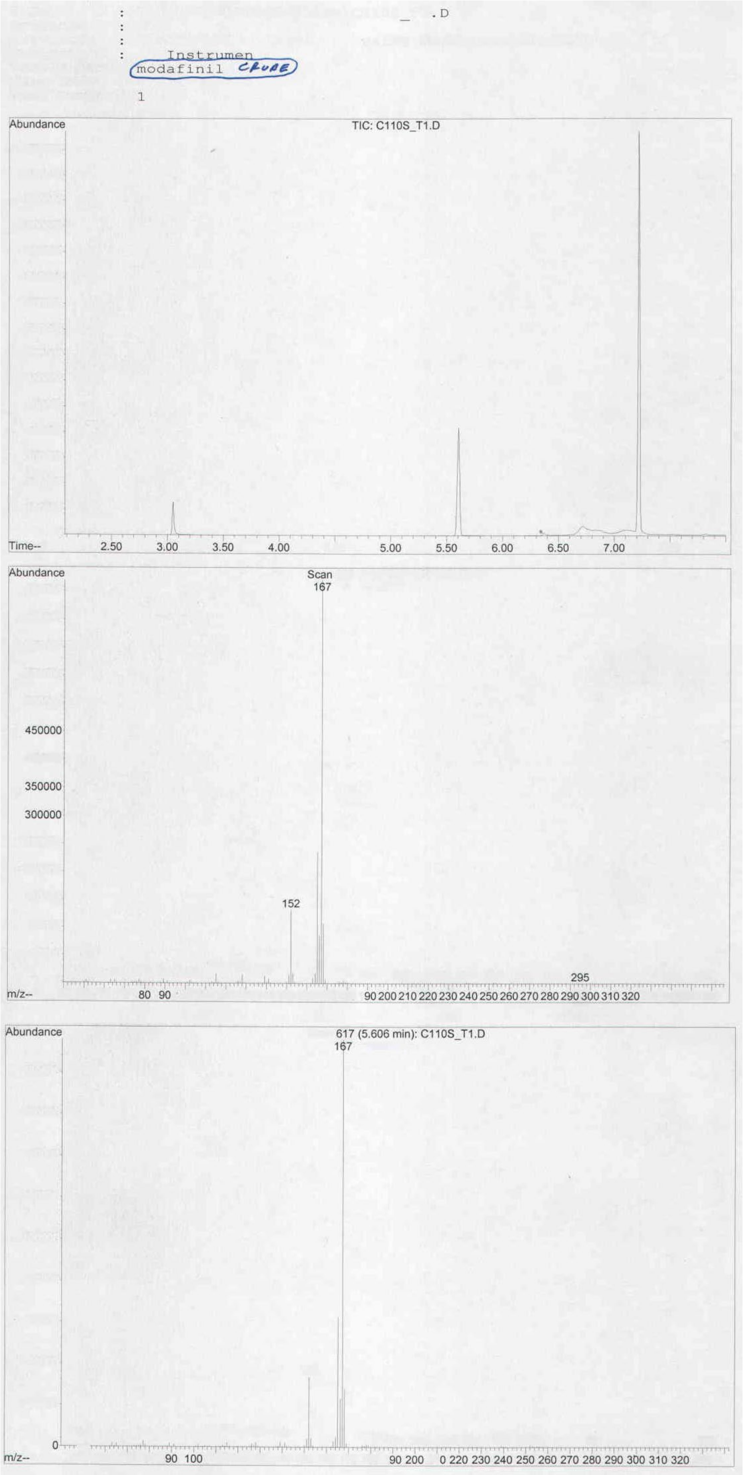

Figure 9. GCMS for crude modafinil

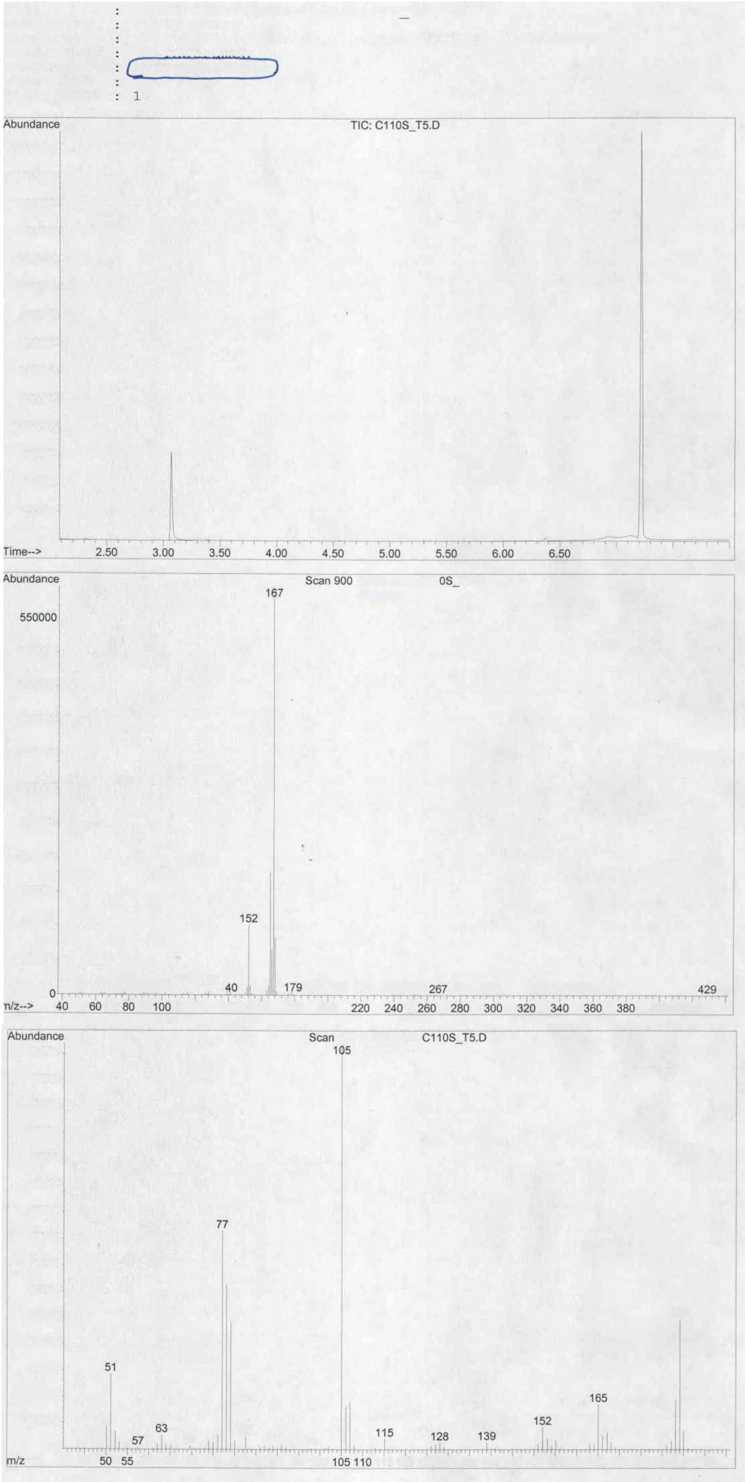

Figure 10. GCMS for recrystallized modafinil

Element %

Trial		N	C	S	H	O
	1	4.985	65.53	11.66	3.088	14.737
	2	4.845	63.91	11.38	3.3	16.565
	3	4.917	64.58	10.91	3.897	15.696
	4	4.917	66.2	11.09	4.56	13.233
	5	5.038	65.25	11.12	4.383	14.09
	6	4.703	65.34	10.65	4.808	14.499
	7	4.543	63.75	10.68	4.798	16.229
	8	4.683	65.18	10.13	4.966	15.041
	9	4.647	65.54	10.25	5.049	14.514
	10	4.913	65.75	10.49	4.248	14.599
	11	4.7	65.54	9.487	4.879	15.394
	12	4.606	64.98	10.92	4.61	14.884
	AVG	4.78925	65.12917	10.73058	4.382167	14.95675
	STDV	.161369	.726404	.590804	.644522	.91748
	Moles	.3419	5.422	.3346	4.3478	.9348
	Atoms in Molecule	1	16	1	13	3

Results and Discussion

Racemic modafinil was synthesized from benzhydrol and analyzed using C and H NMR, IR spectroscopy, GCMS, and elemental analysis

Figure 3 and **Figure 4** depict the H NMR and C NMR, respectively, of (2-benzhydrylsulfanyl)acetic acid, the first intermediate in the procedure. The carbons and hydrogens are assigned to a number matching the peak they correlate to within the spectrum. Figure 5 and Figure 6 depict the H NMR and C NMR, respectively, of the second intermediate,

(2-benzhydrylsulfanyl)acetimide. Again the carbons and hydrogens have number assignments matching the peak which represents them within the spectrum. Now for these two intermediates, the carbons within the arene rings were paired up on the spectra because they had symmetry. **In Figure 7**, the C NMR of modafinil shows that in the final step in forming modafinil the arene rings cannot possibly be symmetrical as there are far too many peaks appearing on the spectrum. Therefore, the oxidation of sulfur must have a major effect upon the structure of the atom, changing the rings just enough to be asymmetrical. The number assignments approximate where the carbons in the rings would likely appear within the spectrum. The alterations in H NMR and C NMR for each intermediate and the final product verifies that changes occurred which match the synthesis of modafinil.

The IR spectrum is depicted in **Figure 9** and the corresponding bonds are described in **Table 1**. **Table 1** lists the types of bonds found within the standard modafinil molecule and assigns a color to each of the different bonds for comparison to the color-coded peaks in **Figure 9**. **Figure 9** accounts for all bonds present in the modafinil molecule, which evidences that the product synthesized in experiment is likely modafinil.

The GCMS results for the final product before and after crystallization can be found in **Figure 10** and **Figure 11**, respectively. The peak occurring after 7 minutes into the analysis is of particular importance. The mass of this compound is 167 g. This compound is important because modafinil contains a weak bond between one of its carbon atoms and a sulfur atom attached to the two benzene rings found in modafinil. This compound breaks off from the main modafinil molecule and causes this 167 g peak after seven minutes into the GCMS analysis. The remaining part of the structure does not appear on the spectrum because the remaining pieces of the original molecule form free radicals which are not registered by the GCMS. GCMS shows all the indications that modafinil was synthesized in this experiment.

Table 2 shows the results of elemental analysis of the samples obtained from 12 separate trials of the experiment by percent of each element. Using the averages of the masses and the atomic masses for each respective element, the ratios of each element in the final product were determined to find the empirical formula for the for the compounds synthesized in the experiment. This empirical formula was found to be $C_{15}H_{13}NO_3S$. However, a number of problems arise when using this particular method to confirm that modafinil was produced in experiment. This method does not take into account the possibility of unwanted water or hydrogen peroxide contaminating the sample. Additionally, the standard deviations for the percentages of some of the elements (particularly oxygen and hydrogen) with respect to the average percentage fell outside of the 5% flexibility range accepted within an accurate study. Therefore, the elemental analysis cannot be used to confirm the production of modafinil because of incorrect percentage distribution due to possible contamination within the samples.

Finally, the mass of the final product was taken and compared to the theoretical yield. The percent yield generated was approximately 50% while the percent yield found in the followed study**(1)** was approximately 67%. While this study followed a similar procedure, it was less effective, potentially due to solvent remaining in solution during different steps in the procedure (such as the precipitation of modafinil out of methanol with water).

Conclusion

Utilizing the analysis methods described above, one can conclude that modafinil was in fact synthesized from benzhydrol using the prescribed synthesis.

Further Study

An interesting further study related to this one would be to separate the enantiomers of modafinil and test their effectiveness against racemic modafinil. Perhaps an even better medication (ie. Fewer side effects) would be produced using only one of the enantiomers.

References

(1) Prisinzano, T.; Podobinski, J.; Tidgewell, K.; Luo, M.; Swenson, D. Science Direct. 2004. 1053 – 1057.
(2) Narcolepsy. Wikipedia. December 2010. < http://en.wikipedia.org/wiki/Modafinil>
(3) Narcolepsy. MayoClinic. 2010 < http://www.mayoclinic.com/health/narcolepsy/DS00345>
(4) Reusch Infrared Spectroscopy. *Typical Infrared Absorption Frequencies.* < http://www2.chemistry.msu.edu/faculty/reusch/VirtTxtJml/Spectrpy/InfraRed/infrared.htm>
(5) Aktoudianakis, E.; Lin, R. J.; Dicks, A. P. Journal of Chemical Education. 2006. Vol. 83 No. 12. 1832-1834.

Transport of Alkali Halides through a Liquid Organic Membrane Containing a Ditopic Salt-Binding Receptor

Joseph M. Mahoney,† Gayathri U. Nawaratna,‡ Alicia M. Beatty,† Peter J. Duggan,*,‡ and Bradley D. Smith*,†

Department of Chemistry and Biochemistry, University of Notre Dame, Notre Dame, Indiana 46556, and School of Chemistry, Monash University, Clayton, Victoria 3800, Australia

Received April 21, 2004

A ditopic receptor is shown to have an impressive ability to recognize and extract the ion pairs of various alkali halides into organic solution. X-ray diffraction analysis indicates that the salts are bound in the solid state as contact ion pairs. Transport experiments, using a supported liquid membrane and high salt concentration in the source phase, show that the ditopic receptor can transport alkali halide salts up to 10-fold faster than a monotopic cation or anion receptor and 2-fold faster than a binary mixture of cation and anion receptors. All transport systems exhibit the same qualitative order of ion selectivity; that is, for a constant anion, the cation selectivity order is $K^+ > Na^+ > Li^+$, and for a constant cation, the anion transport selectivity order is $I^- > Br^- > Cl^-$. The data suggest that with a ditopic receptor, the polarity of the receptor–salt complex can be lowered if the salt is bound as an associated ion pair, which leads to a faster diffusion through the membrane and a higher maximal flux.

Introduction

Liquid extraction and liquid membrane transport are two closely related purification processes that can be employed on an industrial scale.[1] The principle of using phase-transfer agents to selectively complex a target ion has been pursued for some time.[2] Indeed, organic soluble salts with lipophilic cations or anions (such as tetraalkylammonium or tetra-arylborate salts) are often used as phase-transfer agents that operate by ion-exchange processes.[3] In the case of uncharged phase-transfer agents, such as crown ethers, the process is formally a salt transfer since electrical neutrality must be maintained. Typically in this situation, the cation selectivity is determined by the structure of the crown ether; however, the efficiency of the process is strongly dependent on the structure of the counteranion.[4] Currently, there is an active effort to develop molecular complexation systems that simultaneously bind both the cation and the anion. Two strategies can be envisioned: a binary mixture of cation receptor and anion receptor (dual receptor strategy)[5] or a single ditopic receptor with defined cation and anion binding sites (ditopic receptor strategy).[6] Although there have been a large number of recent reports of ditopic salt-binding systems, very few have attempted to evaluate the effectiveness of the design in extraction and membrane transport.[6a,m–o,7] The most detailed transport study was reported in 1999 by Reinhoudt and co-workers.[7a] They compared the membrane transport properties of ditopic calixarene receptors with different mixtures of cation and anion receptors. In the case of KCl transport, they observed that when the source phase's salt concentration was high enough to saturate the membrane-bound receptor, transport with the dual receptor mixture was

* Authors to whom correspondence should be addressed. Phone: (574) 631-8632. Fax: (574) 631-6652. E-mail: smith.115@nd.edu.
† University of Notre Dame.
‡ Monash University.

(1) *Principles and Practices of Solvent Extraction*; Rydberg, J., Nusikas, C., Choppin, G. R., Eds.; Marcel Dekker: New York, 1992. (b) *Fundamentals and Applications of Anion Separations*; Moyer, B. A., Singh, R. P., Eds.; Kluwer: Dordrecht, The Netherlands, 2004.
(2) Gloe, K.; Stephan, H.; Grotjahn, M. *Chem. Eng. Technol.* **2003**, *26*, 1107–1117.
(3) *Phase Transfer Catalysis, Fundamentals, Applications, and Industrial Perspectives*; Starks, C. M., Liotta, C. L., Halpern, M., Eds.; Chapman and Hall: New York, 1994.
(4) Lamb, J. D.; Chrisstensen, J. L.; Izatt, S. R.; Bedke, K.; Astin, M.; Izatt, R. M. *J. Am. Chem. Soc.* **1980**, *102*, 3399–3407.
(5) Recent examples of dual receptor systems that simultaneously complex an anion and a cation: (a) Byriel, K. A.; Gasperov, V.; Gloe, K.; Kennard, C. H. L.; Leong, A. J.; Lindoy, L. F.; Mahinay, M. S.; Pham, H. T.; Tasker, P. A.; Thorp, D.; Turner, P. *J. Chem. Soc., Dalton Trans.* **2003**, 3034–3040. (b) Cafeo, G.; Gargiulli, C.; Gattuso, G.; Kohnke, F. H.; Notti, A.; Occhipinti, S.; Pappalardo, S.; Parisi, M. F. *Tetrahedron Lett.* **2002**, *43*, 8103–8106. (c) Cafeo, G.; Gattuso, G.; Kohnke, F. H.; Notti, A.; Occhipinti, S.; Pappalardo, S.; Parisi, M. F. *Angew. Chem., Int. Ed.* **2002**, *41*, 2122–2126. (d) Qian, Q.; Wilson, G. S.; Bowman-James, K.; Girault, H. H. *Anal. Chem.* **2001**, *73*, 497–503. (e) Kavallieratos, K.; Moyer, B. A. *Chem. Commun.* **2001**, 1620–1621. (f) Kavallieratos, K.; Sachleben, R. A.; Van Berkel, G. J.; Moyer, B. A. *Chem. Commun.* **2000**, 187–188.

10.1021/ic0494859 CCC: $27.50
© 2004 American Chemical Society
Published on Web 08/19/2004

Smoke and liquid smoke. Study of an aqueous smoke flavouring from the aromatic plant *Thymus vulgaris* L

María D Guillén* and María J Manzanos
Tecnología de los Alimentos, Facultad de Farmacia, Universidad del País Vasco, Paseo de la Universidad 7, E-01006 Vitoria, Spain

Abstract: An aqueous smoke flavouring from *Thymus vulgaris* L was obtained. The qualitative and quantitative composition of its dichloromethane extract was studied by gas chromatography and gas chromatography/mass spectrometry. In addition to the common smoke components such as aldehydes, ketones, diketones, esters, acids, furan and pyran derivatives, alkyl aryl ethers, phenol, guaiacol and syringol derivatives, some terpenic compounds were detected. Differences between this aqueous liquid smoke and others from various kinds of wood are due not only to the absence or presence of some compounds but also to the proportions of the different groups of components present in the mixture. Some aldehydes, esters, guaiacol and its derivatives and terpenic compounds contribute to the overall odour of this liquid smoke more significantly than the ketones, furan and pyran derivatives, acids, phenol and its derivatives and syringol. The yield in smoke components obtained from the pyrolysis of *Thymus vulgaris* L at 488 °C is of a similar order to that obtained from other woods.
© 1999 Society of Chemical Industry

Keywords: smoke; *Thymus vulgaris* L; yield; composition; gas chromatography; gas chromatography/mass spectrometry

INTRODUCTION

The smoking of foods is one of the most ancient food-preserving processes and, in some communities, one of the most important. The fuel used to smoke foods is different in each region and, because of availability, cost, abundance and numerous other factors, many types of fuels have been used, namely coconut husks, maize cobs, grass, cow dung, peat, dried sheep manure, fresh red mangrove wood, oak wood, beech wood, hickory wood, mesquite wood, etc.[1–5]

It is well accepted that the type of fuel used to produce smoke significantly influences the organoleptic properties of the resulting smoke. The organoleptic smoke properties depend on the smoke composition, which in turn depends, among other factors, on the fuel composition. The most common fuel used is wood, which consists of approximately 50% cellulose, 25% hemicellulose and 25% lignin; the lignin structure of softwood is different from that of hardwood. In addition to the main components mentioned, woods differ in minor components such as terpenes and related compounds, fatty acids, other carbohydrates, polyhydric alcohols, nitrogen and phenol compounds as well as inorganic constituents.[6] There is no agreement between authors about which kind of wood or mixture of woods supplies the best organoleptic properties to smoked food,[2,6] owing to customs and culinary habits.

Some authors have pointed out that the smokes obtained from aromatic plants have interesting organoleptic properties for food smoking.[7,8] However, to the best of our knowledge, the yield and qualitative and quantitative composition of these kinds of smokes are not known; only the study of the antimicrobial activity of spice smoke condensates from onion, oregano, peppercorns, chilli pepper and sausage seasoning, together with the phenols and heterocyclics identified in each smoke condensate, have been reported.[9] In this paper the production of liquid smoke from *Thymus vulgaris* L, together with the composition of its extract in dichloromethane, studied by gas chromatography and gas chromatography/mass spectrometry, is reported.

Thyme is a popular aromatic plant widely used as a spice in food processing, perfumes and popular medicine. The genus *Thymus* belongs to the family

* Correspondence to: María D Guillén, Tecnología de los Alimentos, Facultad de Farmacia, Universidad del País Vasco, Paseo de la Universidad no 7, E-01006 Vitoria, Spain
Contract/grant sponsor: Comisión Interministerial de Ciencia y Tecnología; contract/grant number: ALI97-1095
Contract/grant sponsor: Ministerio de Educación y Ciencia
(*Received 14 April 1998; revised version received 18 January 1999; accepted 11 March 1999*)

JOURNAL OF AGRICULTURAL AND FOOD CHEMISTRY

Supercritical CO_2 Extract of *Cinnamomum zeylanicum*: Chemical Characterization and Antityrosinase Activity

BRUNO MARONGIU,† ALESSANDRA PIRAS,† SILVIA PORCEDDA,† ENRICA TUVERI,† ENRICO SANJUST,§ MASSIMO MELI,§ FRANCESCA SOLLAI,§ PAOLO ZUCCA,§ AND ANTONIO RESCIGNO*,§

Dipartimento di Scienze Chimiche and Dipartimento di Scienze e Tecnologie Biomediche, Università di Cagliari Cittadella Universitaria, I-09042 Monserrato (CA), Italy

The volatile oil of the bark of *Cinnamomum zeylanicum* was extracted by means of supercritical CO_2 fluid extraction in different conditions of pressure and temperature. Its chemical composition was characterized by GC-MS analysis. Nineteen compounds, which in the supercritical extract represented >95% of the oil, were identified. (*E*)-Cinnamaldehyde (77.1%), (*E*)-β-caryophyllene (6.0%), α-terpineol (4.4%), and eugenol (3.0%) were found to be the major constituents. The SFE oil of cinnamon was screened for its biological activity about the formation of melanin in vitro. The extract showed antityrosinase activity and was able to reduce the formation of insoluble flakes of melanin from tyrosine. The oil also delayed the browning effect in apple homogenate. (*E*)-Cinnamaldehyde and eugenol were found to be mainly responsible of this inhibition effect.

KEYWORDS: Supercritical CO_2 extraction; volatile oil; *Cinnamomum zeylanicum*; tyrosinase; melanin; inhibition

INTRODUCTION

Cinnamon (*Cinnamomum zeylanicum* Blume syn. *C. verum* J.S. Presl, Lauraceae) is a medium-sized tree native to Sri Lanka and tropical Asia. It is also known as Ceylon cinnamon, true cinnamon, Ceylon-Zimtbaum, and cannelle de Ceylan (*1*). The term cinnamon is also used to indicate other species: *Cinnamomum loureirii* Nees syn. *C. obtusifolium* Nees var. *loureirii* Perr et Eb. and *C. burmanii* (Nees & T. Nees) Blume syn. *C. pedunculata* J. S. Presl.

Trees of *C. zeylanicum* are cultivated in Sri Lanka and India to obtain leaves, bark, and roots. The essential oils, obtained by steam distillation from different parts of this species, are dissimilar, and the oil obtained from the bark, called oil of cinnamon Ceylon, is considered to be superior for its aroma. Essential oils obtained from twigs, pedicels, buds, flowers, and fruits are characterized by a very low content (<4.0%) of (*E*)-cinnamaldehyde and larger and variable amounts of (*E*)-cinnamyl acetate, (*E*)-β-caryophyllene, and linalool (*2*). Cinnamon leaf oil has been reported to show a notable antifungal activity against *Botrytis cinerea* (*3*). The chemical composition of the volatile oil has been also determined in fruits of *C. zeylanicum* (*4*). Simic et al. reported that a commercial oil, chiefly constituted by (*E*)-cinnamaldehyde (62.79%), limonene (8.31%), and linalool (7.09%), showed a very strong antifungal activity against 17 different micromycetes (*5*). Pawar and Thaker, who tested 75 essential oils against the fungus *Aspergillus niger*, found that the highest inhibitory effect was exhibited by the oil obtained from the bark of cinnamon (*6*). The antioxidant activity of this oil is also known (*7, 8*).

Hydrodistillation or steam distillation, even when it does not induce extensive phenomena of hydrolysis and thermal degradation, gives in any case a product with a characteristic off-odor (*9*). Solvent extraction can give oil, but due to a high content of waxes and/or other high molecular mass compounds, often gives rise to a concrete with a scent very similar to the material from which it was derived. However, small amounts of organic solvents can pollute the extraction product.

Supercritical fluid extraction (SFE) is a good technique for the production of flavors and fragrances from natural materials and can constitute a valid alternative to both of the above-mentioned processes. In fact, compressed carbon dioxide, CO_2, is able to solubilize hydrocarbons and oxygenated mono- and sesquiterpenes (*10*), the main essential oil constituents. The separation of the extractant is easy, hydrolysis and thermal degradation are practically absent, and the extract retains the organoleptic features of the starting material. Possible residues do not cause a risk for human health. Indeed, CO_2, besides being

* Address correspondence to this author at the Department of Biomedical Sciences and Technologies, University of Cagliari, Cittadella Universitaria, I-09042 Monserrato (CA), Italy (e-mail rescigno@unica.it; fax +39 070 6754527; telephone +39 070 6754516).
† Dipartimento di Scienze Chimiche.
§ Dipartimento di Scienze e Tecnologie Biomediche.

10.1021/jf071938f CCC: $37.00 © 2007 American Chemical Society
Published on Web 10/30/2007

Stereoselective Potencies and Relative Toxicities of Coniine Enantiomers

Stephen T. Lee,* Benedict T. Green, Kevin D. Welch, James A. Pfister, and Kip E. Panter

Poisonous Plant Research Laboratory, Agricultural Research Service, United States Department of Agriculture, 1150 E. 1400 N., Logan, Utah 84341

Received June 24, 2008

Coniine, one of the major toxic alkaloids present in poison hemlock (*Conium maculatum*), occurs in two optically active forms. A comparison of the relative potencies of (+)- and (−)-coniine enantiomers has not been previously reported. In this study, we separated the enantiomers of coniine and determined the biological activity of each enantiomer *in vitro* and *in vivo*. The relative potencies of these enantiomers on TE-671 cells expressing human fetal nicotinic neuromuscular receptors had the rank order of (−)-coniine > (±)-coniine > (+)-coniine. A mouse bioassay was used to determine the relative lethalities of (−)-, (±)-, and (+)-coniine *in vivo*. The LD_{50} values of the coniine enantiomers were 7.0, 7.7, and 12.1 mg/kg for the (−)-, (±)-, and (+)- forms of coniine, respectively. The results from this study demonstrate that there is a stereoselective difference in the *in vitro* potencies of the enantiomers of coniine that directly correlates with the relative toxicities of the enantiomers *in vivo*.

Introduction

Conium maculatum L., commonly referred to as poison hemlock or spotted hemlock, is a member of the Umbelliferae family and is known worldwide for its acute toxicity to animals. The affected species include cattle, sheep, goats, horses, elk, pigs, poultry, range turkeys, quail, and humans (*1*). The principle toxins in *C. maculatum* have been identified as piperidine alkaloids of which coniine (**1**) and γ-coniceine are the most prevalent and account for most of the toxicity (*1, 2*).

Coniine (**1**) is a nicotinic acetylcholine receptor (nAChR) agonist. Whole cell current recordings of *Xenopus* oocytes expressing fetal rat muscle type nAChR exhibited a dose-dependent response to coniine (*3*). The IC_{50} values of coniine (**1**) for the displacement of [^{125}I]-α-bungarotoxin or [^{3}H]-cytisine from chick embryonic muscle and brain preparations have been shown to be in the micromolar range (*4*). *In vivo*, it is documented that coniine (**1**) causes a biphasic response of first stimulation followed by blockade of nicotinic receptors in the central nervous system and periphery (*1, 5*). Clinical signs of poisoning include protrusion of the nictitating membrane, excessive salivation, and frequent urination and defecation, loss of coordination, muscle weakness, and tremors followed by collapse and death due to respiratory failure (*5, 6*).

Coniine (**1**), as found in *C. maculatum*, is a mixture of (+)- and (−)-enantiomeric forms, with the (+)-form as the predominant enantiomer (*2*). Stereochemical integrity is a significant factor in determining the specificity of biological effects of chiral compounds, both in natural products and synthetic compounds (*7*). For example, the (+)-form of ketamine is four times more potent in humans than the (−)-form, and in human neonates, the (−)-enantiomer of ibuprofen is more effective than the (+)-enantiomer (*8*). No toxicity studies have been reported with both of the coniine (**1**) enantiomers measured separately. Single oral doses of enantiomeric mixtures of coniine (**1**) have been shown to be toxic to ewes, mares, cows, quails, chicks, and turkey chicks (*1*). The oral LD_{50} of an enantiomeric mixture of coniine in mice is reported to be 100 mg/kg (*1*). Little is known about the specific toxicities of the individual coniine enantiomers *in vivo* and *in vitro*.

Previously, we reported the separation, isolation, and toxicity of anabasine (**2**) enantiomers from *Nicotiana glauca* and ammodendrine (**3**) enantiomers from *Lupinus formosus* plants (*9, 10*). In both cases, we observed differential toxicities between the individual ammodendrine enantiomers and individual anabasine enantiomers. These results led us to investigate the toxicity of the individual coniine (**1**) enantiomers. To separate coniine (**1**), we used preferential crystallization with (+)- and (−)-mandelic acid. Enantiomers of compounds often have very different biological activities; thus, it was important to measure the relative potencies of the (+)- and (−)-coniines (**1**) individually. In our study, the neuromuscular nicotinic receptor (nAChR) agonist potencies of coniine (**1**) enantiomers were assessed using a human tumor cell line (TE-671) expressing fetal muscle type nAChRs, and the toxicities of the enantiomers were measured in a mouse lethality bioassay.

Materials and Methods

Materials. Ammonium hydroxide, *N,N*-dimethyl formamide (DMF), and sulfuric acid were obtained from Fisher Scientific (Pittsburgh, PA). (±)-Epibatidine was obtained from Sigma-Aldrich (St Louis, MO) and ammonium acetate from VWR (Bristol, CT). Fetal bovine serum and penicillin/streptomycin were from Media Tech, Inc. (Herndon, VA), Dulbecco's modified Eagle's medium was from the American type Culture Collection (Manassas, VA), and the fluorescence dye kits were purchased from Molecular Devices (Sunnyvale, CA).

Isolation of (+)- and (−)-Coniine. Coniine (**1**) was isolated using previously described methods (*11*). (±)-Coniine (1.0102 g, 7.95 mmol) was added to a 50 mL screw top glass test tube. (±)-Coniine-HCl was prepared by bubbling HCl gas through the 40 mL diethyl ether solution resulting in a fine white precipitate. The diethyl ether was filtered from the crystals, the crystals collected, and dried *in vacuo*. The (±)-conine-HCl was

* Corresponding author. Tel: (435) 752-2941. Fax: (435) 753-5681. E-mail: stephen.lee@ars.usda.gov.

10.1021/tx800229w CCC: $40.75 This article not subject to U.S. Copyright. Published 2008 by American Chemical Society.
Published on Web 09/03/2008

"As Simple as Possible, but Not Simpler"—The Case of Dehydroascorbic Acid

Robert C. Kerber
Department of Chemistry, Stony Brook University, Long Island, NY 11794; *rkerber@notes.cc.sunysb.edu*

The injunction to "make everything as simple as possible, but not simpler", ascribed to Albert Einstein, characterizes a continuing dilemma of the teacher–scholar and of the textbook author. In presenting scientific material, the question becomes which simplifications are justified for the sake of clarity and which represent unacceptable distortions of reality? A related question is how quickly new knowledge should be incorporated into the canon. Is it more urgent or less urgent if it complicates a previously simple picture?

Certainly in an age where new editions of textbooks regularly appear every three years or so, it is hard to justify delays of decades in incorporating new knowledge or understanding. But the case of dehydroascorbic acid (the oxidized form of ascorbic acid, vitamin C), invariably described in textbooks as a tricarbonyl compound, **1** (Figure 1), raises questions about our effectiveness in correcting old oversimplifications and incorporating new knowledge. Doubts were expressed about the credibility of structure **1** as long ago as the 1940s, and definitive results disproving that structure were published in the 1970s (vide infra), but still the structure persists in our textbooks.

In the 19th century, chemistry "textbooks" were scholarly summaries of the state of contemporaneous knowledge, written by the foremost practitioners. As the body of knowledge grew, these evolved on the one hand into encyclopedias, compendia, and databases for experts and on the other hand, into schoolbooks for students. The latter were necessarily smaller (still not small enough, in many cases; ref *1*) and less up-to-date, and they have become increasingly similar to one another as market forces have favored conformity in presentation and coverage. But this author finds it difficult to understand why that conformity should embrace misleading terminology *(2)* and erroneous structures. The textbook structure of oxidized ascorbic acid provides a case in point.

Dehydroascorbic Acid

Ascorbic acid owes its biochemical and commercial importance to its effectiveness as a reducing agent. So the determination of the structure of ascorbic acid in the 1930s soon led to discussions of the structures of the one- and two-electron oxidized forms *(3)*. The latter came to be referred to as "dehydroascorbic acid (DHA)" *(4)*, a rather unsatisfactory name given its lack of acidity. Definitive structure determination was hindered by its facile hydrolysis and failure to form crystals, but the earliest structural suggestion *(3)* was for a dihydrate, **2**. The absence of color in DHA was understood to be inconsistent with the diketolactone structure **1** by early workers *(5)*, but nevertheless representations as **1** came to be common.

In 1970, a preliminary communication described the crystal structure of DHA crystals grown from 95% acetic acid containing 0.01 M hydrochloric acid *(6)*. The full paper was published in 1972 *(7)*. The structure revealed was that of a symmetrical dimer, $C_{12}H_{12}O_{12}$, **3**, having a twist-boat dioxane ring assembled from the O2, C2 (a hemiacetal), and C3 (an acetal incorporating the primary alcohol group of the side chain) atoms of each dehydroascorbate unit. An analogous dimeric crystal structure was subsequently obtained for the tetraacetate derivative of **3** *(8)*. Commercial DHA is composed of this dimeric solid, but the dimer is clearly the result of several chemical steps following the initial oxidation of ascorbic acid; a key step is the formation of the second five-membered ring, analogous to formation of furanose rings by carbohydrates.

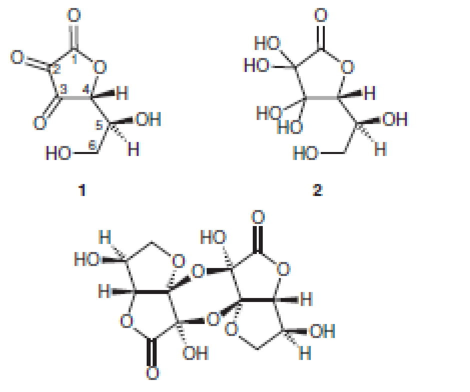

Figure 1. Structures associated with dehydroascorbic acid (DHA): textbook DHA, **1**; dihydrate, **2**; dimer, **3**.

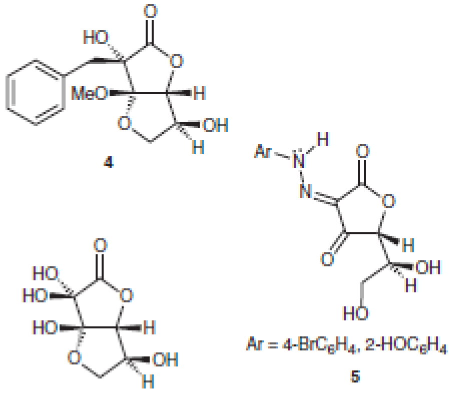

Figure 2. Structures associated with DHA: C2-benzylated, **4**; hydrazones, **5**; bicyclic monohydrate, **6**.

JOURNAL OF
AGRICULTURAL AND FOOD CHEMISTRY

J. Agric. Food Chem. 2008, 56, 12037–12045 12037

Corn Starch Gel for Yeast Cell Entrapment. A View for Catalysis of Wine Fermentation

Panagiotis Kandylis, Amalia Goula, and Athanasios A. Koutinas*

Food Biotechnology Group, Department of Chemistry, University of Patras, GR-26500 Patras, Greece

A new biocatalyst was prepared by immobilization of *Saccharomyces cerevisiae* AXAZ-1 yeast cells in the matrix of corn starch gel. This biocatalyst was used for repeated batch fermentations of glucose and grape must at various sugar concentrations (110–280 g/L) and low-temperature winemaking (5 °C). The biocatalyst retained its operational stability for a long period, and it was proved to be capable of producing dry and semisweet wines. The produced wines were analyzed for volatile byproducts by GC and GC-MS, and the results showed an increase in the number and amount of esters by immobilized cells. In addition, an increase in the percentages of esters and a decrease in those of alcohols with the drop of fermentation temperature were reported. The activation energy (E_a) was lower (~36%) and the reaction rate constant (k) was higher (~78% at 30 °C and ~265% at 15 °C) in the case of immobilized cells compared to free cells, especially at low temperatures. These results show that corn starch gel may act as a promoter for the enzymes that are involved in the process or as a catalyst of the alcoholic fermentation and can explain the capability of immobilized cells for extremely low-temperature winemaking. Therefore, these results open a new way for research to find new catalysts in biotechnological processes.

KEYWORDS: Immobilization; wine; volatiles; GC-MS; corn starch gel; activation energy

INTRODUCTION

In recent years several immobilized cell systems have been proposed for use in bioconversions such as alcoholic fermentation. This can be attributed to their numerous advantages, such as improved productivity, compared to free cell systems. Among the techniques that have been proposed for cell immobilization, the most important are mechanical containment behind a barrier, entrapment within a porous matrix, cell flocculation (aggregation), and immobilization on solid carrier surfaces (*1*).

Various materials (synthetic, natural, organic, and inorganic) have been used as supports of cell immobilization (*2–7*). In recent years several supports of food grade purity, such as delignified cellulosic materials (*8*), gluten pellets (*9*), brewer's spent grains (*10*), dried figs (*11*), dried raisin berries (*12*), fruit pieces (*13*), and potatoes (*14*), have been proposed as ideal for yeast immobilization for winemaking and brewing. The immobilization of alcohol resistant and cryotolerant yeasts on these supports led to low-temperature winemaking and brewing, resulting in wines and beers with improved taste and aroma. Although several immobilization supports have been proposed for alcoholic fermentation, only a few find application at the industrial level, and therefore the search for new material is of great interest.

In previous studies (*8*, *15*) the use of delignified cellulosic material as support for yeast immobilization proved to be very effective for low-temperature winemaking and brewing. These studies, based on a theoretical approach of the Arrhenius equation, showed that the low-temperature fermentations obtained by the immobilized cells are responsible for the reduction of the activation energy, E_a, by the presence of this support. In a recent study, using yeast cells immobilized on potatoes (*14*), the reduced activation energy and the higher reaction rate constant in the case of immobilized cells led to the conclusion that potatoes may behave as a catalyst or a promoter of the enzymes involved in the process.

The aim of the present study was to evaluate the use of corn starch gel as a support for yeast immobilization. The new biocatalyst was used for dry and semisweet winemaking at 27 °C and also for low-temperature wine making (5 °C). The effect of initial sugar concentration and that of temperature on volatile formation and fermentation kinetics during winemaking were also examined. Finally, the possible catalytic effects of the immobilization support to the enzymes that are involved in the alcoholic fermentation were studied and the reaction rate constants and activation energies of free and immobilized systems calculated.

MATERIALS AND METHODS

Yeast Strains and Media. The alcohol resistant and cryotolerant *Saccharomyces cerevisiae* AXAZ-1, isolated from a Greek vineyard plantation (*16*), was used in the present study. It was grown on culture medium consisting of 4 g of yeast extract/L, 1 g of $(NH_4)_2SO_4$/L, 1 g of KH_2PO_4/L, 5 g of $MgSO_4 \cdot 7H_2O$/L, and 40 g of glucose monohydrate/L at 30 °C and harvested at 4000 rpm for 10 min. All media

* Author to whom correspondence should be addressed (telephone +30 2610 997104; fax +30 2610 997105; e-mail A.A.Koutinas@upatras.gr).

10.1021/jf802476w CCC: $40.75 © 2008 American Chemical Society
Published on Web 11/26/2008

Energy & Fuels **2008**, *22*, 1358–1364

"Designer" Biodiesel: Optimizing Fatty Ester Composition to Improve Fuel Properties†

Gerhard Knothe*

National Center for Agricultural Utilization Research, Agricultural Research Service, United States Department of Agriculture, Peoria, Illinois 61604

Received October 29, 2007. Revised Manuscript Received January 3, 2008

Biodiesel is a domestic and renewable alternative with the potential to replace some of the petrodiesel market. It is obtained from vegetable oils, animal fats, or other sources with a significant content of triacylglycerols by means of a transesterification reaction. The fatty acid profile of biodiesel thus corresponds to that of the parent oil or fat and is a major factor influencing fuel properties. Besides being renewable and of domestic origin, advantages of biodiesel compared to petrodiesel include biodegradability, higher flash point, reduction of most regulated exhaust emissions, miscibility in all ratios with petrodiesel, compatibility with the existing fuel distribution infrastructure, and inherent lubricity. Technical problems with biodiesel include oxidative stability, cold flow, and increased NO_x exhaust emissions. Solutions to one of these problems often entail increasing the problematic behavior of another property and have included the use of additives or modifying the fatty acid composition, either through physical processes, such as winterization, or through genetic modification. Methyl oleate has been proposed as a suitable major component of biodiesel in this connection. In this work, the properties of various potential major components of biodiesel are examined and compared. For example, while methyl oleate has been suggested as such a major component, methyl palmitoleate has advantages compared to methyl oleate, especially with regards to low-temperature properties. Other materials that are examined in this connection are short-chain (C_8–C_{10}) saturated esters, with only C_{10} esters appearing suitable. It is also suggested that to obtain biodiesel fuel with favorable properties, it is advantageous for the fuel to consist of only one major component in as high a concentration as possible; however, mixtures of components with advantageous properties as described here may also be acceptable.

Introduction

The replacement of fossil fuels to secure future energy supplies continues to be a major concern. In this connection, biodiesel[1,2] is an alternative to petroleum-based diesel fuel (petrodiesel). A reaction commonly referred to as transesterification yields biodiesel from vegetable oils or other materials largely comprised of triacylglycerols, such as animal fats or used frying oils, and monohydric alcohols. The resulting monoalkyl esters possess fuel and physical properties that are competitive with petrodiesel. The fatty acid profile of biodiesel is identical to that of the parent oil or fat. Advantages of biodiesel include domestic origin, renewability, biodegradability, higher flash point, inherent lubricity, reduction of most regulated exhaust emissions, as well as miscibility with petrodiesel. An essential feature of biodiesel is that its fatty acid composition corresponds to that of its parent oil or fat. Thus, biodiesel fuels derived from different sources can have significantly varying fatty acid profiles and properties.

Various specifications that a biodiesel fuel must meet are contained in biodiesel standards, such as American Society for Testing and Materials (ASTM) D6751 and EN 14214 in Europe. While many of these specifications are related to fuel quality issues, such as completeness of the transesterification reaction or storage conditions, several parameters directly depend upon the fatty acid composition of the biodiesel fuel. Among these specifications are cetane number, kinematic viscosity, oxidative stability, and cold-flow properties in form of the cloud point or cold-filter plugging point. Other important issues to consider that are influenced by fatty ester composition but are not contained in biodiesel standards are exhaust emissions, lubricity, and heat of combustion.

The most common fatty esters contained in biodiesel are those of palmitic (hexadecanoic) acid, stearic (octadecanoic) acid, oleic (9(*Z*)-octadecenoic) acid, linoleic (9(*Z*),12(*Z*)-octadecadienoic) acid, and linolenic (9(*Z*),12(*Z*),15(*Z*)-octadecatrienoic) acid. This holds for biodiesel feedstocks, such as soybean, sunflower, rapeseed (as canola), palm, and peanut oils. Some tropical oils, such as coconut oil, contain significant amounts of shorter chain acids, such as lauric (dodecanoic) acid. A variety of other fatty acids are found in minor amounts in virtually all oils and fats used as biodiesel feedstocks.

Technical problems with biodiesel that have persisted to the present are oxidative stability, poor low-temperature properties, and a slight increase in NO_x exhaust emissions, although the latter problem may fade over time with the advent of new exhaust emission control technologies. Solving these problems simultaneously has proven difficult because the solution to one of the problems often aggravates another problem. This behavior can be largely traced to the dependence of fuel properties on

† Disclaimer: Product names are necessary to report factually on available data; however, the USDA neither guarantees nor warrants the standard of the product, and the use of the name by USDA implies no approval of the product to the exclusion of others that may also be suitable.

* To whom correspondence should be addressed: USDA/ARS/NCAUR, 1815 N. University St., Peoria, IL 61604. Telephone: (309) 681-6112. Fax: (309) 681-6340. E-mail: gerhard.knothe@ars.usda.gov.

(1) *The Biodiesel Handbook*; Knothe, G., Krahl, J., Van Gerpen, J., Eds.; AOCS Press: Champaign, IL, 2005.
(2) Mittelbach, M.; Remschmidt, C. *Biodiesel—The Comprehensive Handbook*; M. Mittelbach: Graz, Austria, 2004.

10.1021/ef700639e This article not subject to U.S. Copyright. Published 2008 by the American Chemical Society
Published on Web 02/19/2008

Discovery of a Novel Series of Peroxisome Proliferator-Activated Receptor α/γ Dual Agonists for the Treatment of Type 2 Diabetes and Dyslipidemia

Kun Liu,* Libo Xu, Joel P. Berger, Karen L. MacNaul, Gauchao Zhou, Thomas W. Doebber, Michael J. Forrest, David E. Moller, and A. Brian Jones

Merck Research Laboratories, P.O. Box 2000, Rahway, New Jersey 07065

Received December 10, 2004

Abstract: A series of 2-aryloxy-2-methyl-propionic acid compounds and related analogues were designed, synthesized, and evaluated for their PPAR agonist activities. 2-[(5,7-Dipropyl-3-trifluoromethyl)-benzisoxazol-6-yloxy]-2-methylpropionic acid (**4**) was identified as a PPARα/γ dual agonist with relative PPARα selectivity and demonstrated potent efficacy in lowering both glucose and lipids in animal models without causing body weight gain. The PPARα activity of **4** appeared to have played a significant role in lowering glucose levels in db/db mice.

Type 2 diabetes is a multifactorial disease characterized by insulin resistance and/or abnormal insulin secretion. This metabolic disorder, which accounts for more than 90% of all diabetes, afflicts an estimated 6% of the adult US population. Its worldwide frequency is expected to grow by 6% annually, reaching a potential total of 200–300 million cases in 2010.[1] Type 2 diabetes and insulin resistance are frequently associated with dyslipidemia and a markedly increased incidence of atherosclerotic cardiovascular disease.[2] Glycemic control is traditionally considered the first priority in the treatment of diabetic patients. However, results from the UK Prospective Diabetes Study have shown that intensive control of hyperglycemia can attenuate microvascular complications, but may not reduce macrovascular disease, the main cause of morbidity and mortality in type 2 diabetes.[3] Accordingly, more aggressive therapeutic approaches that not only lower glucose but also simultaneously reduce cardiovascular risk factors associated with type 2 diabetes are clearly in high demand.

The peroxisome proliferator-activated receptors (PPARs) are ligand-activated transcription factors belonging to the nuclear receptor superfamily.[4] There are three PPAR subtypes encoded by distinct genes: PPARα (NR1C1), PPARδ (NR1C2), and PPARγ (NR1C3).[5] These receptors are important regulators in multiple physiological pathways, such as glucose homeostasis, fatty acid metabolism, inflammation, and cellular differentiation.[6] Glitazones and fibrates are two classes of PPAR drugs currently being marketed for the treatments of insulin resistance and dyslipidemia, respectively. Glitazones (pioglitazone and rosiglitazone (**1**)) are insulin sensitizers functioning through PPARγ activation.[7] Although a direct link between PPARγ and insulin sensitivity has not been fully established, one theory suggests that the hypoglycemic effects of glitazones are, at least in part, derived from their lipid-modulating effects.[8] Activation of PPARγ increases free fatty acid (FFA) uptake in adipose tissue, thus reducing the FFA inhibition of glucose disposal in skeletal muscle. While glitazones lower glucose and improve insulin sensitivity, they suffer from several adverse effects, including weight gain and edema.On the other hand, PPARα-activating fibrates have enjoyed a good safety profile in humans over the past two decades.[9] These hypolipidemics are effective in lowering triglycerides and raising HDL levels and have been demonstrated to significantly reduce the coronary events in type 2 diabetes patients.[10] The lipid modulating effect of fibrates is principally mediated by PPARα-regulated expressions of genes involved in lipid and lipoprotein metabolism in liver, leading to enhanced hepatic fatty acid oxidation, which may also be the basis of the observed independent hypoglycemic effect of fibrates.[11] In addition, fibrates have been shown to reduce body weight in rodents, in sharp contrast to PPARγ activators, which promote weight gain.[11a,12] Current fibrate drugs are low affinity PPARα ligands with only clinically marginal hypoglycemic effects. Further studies will be needed to establish whether more potent PPARα agonists may treat type 2 diabetes without PPARγ-related liabilities.

Chart 1. PPAR Agonists

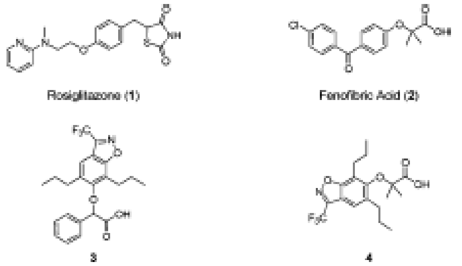

We herein report the structural design, synthesis, and preclinical evaluation of a novel series of PPARα/γ dual agonists. Compound **4** demonstrated potent PPARα agonist activity in a cell-based reporter gene expression assay, while only weakly activating PPARγ. It lowered both plasma glucose and lipid in animal models without causing body weight increase, an adverse effect frequently associated with PPARγ agonists.

We recently reported the discovery of a new class of *O*-arylmandelic acid PPAR agonists.[13] Some of them, exemplified by **3**, showed PPARα/γ dual activity in vitro and good in vivo efficacy in lowering both glucose and lipids in rodents. On the other hand, fibrates, such as fenofibrate, are weak PPARα agonists and have enjoyed an excellent tolerability profile. Fenofibrate is the prodrug of the active parent compound, fenofibric acid (**2**). For the structural design of novel PPAR compounds, we combined the putative pharmacophores of these two classes of compounds and generated several hybrid

* To whom correspondence should be addressed. Phone: 732-594-7445. Fax: 732-594-9556. E-mail: kun_liu@merck.com.

10.1021/jm048993p CCC: $30.25 © 2005 American Chemical Society
Published on Web 03/10/2005

JOURNAL OF
AGRICULTURAL AND
FOOD CHEMISTRY

Comparison of Antioxidant Potency of Commonly Consumed Polyphenol-Rich Beverages in the United States

NAVINDRA P. SEERAM,† MICHAEL AVIRAM,§ YANJUN ZHANG,† SUSANNE M. HENNING,† LYDIA FENG,† MARK DREHER,# AND DAVID HEBER*,†

Center for Human Nutrition, David Geffen School of Medicine, University of California, Los Angeles, California 90095; Lipid Research Laboratory, Technion Faculty of Medicine, Rambam Medical Center, Haifa, Israel; and POM Wonderful, LLC, Los Angeles, California 90064

A number of different beverage products claim to have antioxidant potency due to their perceived high content of polyphenols. Basic and applied research indicates that pomegranate juice (PJ), produced from the Wonderful variety of *Punica granatum* fruits, has strong antioxidant activity and related health benefits. Although consumers are familiar with the concept of free radicals and antioxidants, they are often misled by claims of superior antioxidant activity of different beverages, which are usually based only on testing of a limited spectrum of antioxidant activities. There is no available direct comparison of PJ's antioxidant activity to those of other widely available polyphenol-rich beverage products using a comprehensive variety of antioxidant tests. The present study applied (1) four tests of antioxidant potency [Trolox equivalent antioxidant capacity (TEAC), total oxygen radical absorbance capacity (ORAC), free radical scavenging capacity by 2,2-diphenyl-1-picrylhydrazyl (DPPH), and ferric reducing antioxidant power (FRAP)]; (2) a test of antioxidant functionality, that is, inhibition of low-density lipoprotein (LDL) oxidation by peroxides and malondialdehyde methods; and (3) evaluation of the total polyphenol content [by gallic acid equivalents (GAEs)] of polyphenol-rich beverages in the marketplace. The beverages included several different brands as follows: apple juice (3), açaí juice (3), black cherry juice (3), blueberry juice (3), cranberry juice (3), Concord grape juice (3), orange juice (3), red wines (3), iced tea beverages (10) [black tea (3), green tea (4), white tea (3)], and a major PJ available in the U.S. market. An overall antioxidant potency composite index was calculated by assigning each test equal weight. PJ had the greatest antioxidant potency composite index among the beverages tested and was at least 20% greater than any of the other beverages tested. Antioxidant potency, ability to inhibit LDL oxidation, and total polyphenol content were consistent in classifying the antioxidant capacity of the polyphenol-rich beverages in the following order: PJ > red wine > Concord grape juice > blueberry juice > black cherry juice, açaí juice, cranberry juice > orange juice, iced tea beverages, apple juice. Although in vitro antioxidant potency does not prove in vivo biological activity, there is also consistent clinical evidence of antioxidant potency for the most potent beverages including both PJ and red wine.

INTRODUCTION

Pomegranate (*Punica granatum* L.) fruits are popularly consumed in beverage forms such as pomegranate juice (PJ). Several studies have been conducted on a well-characterized PJ made from the Wonderful variety of *P. granatum* fruits (*1–6*). Basic and applied research in animals and humans indicates that this PJ has potent antioxidant activity, which has been linked to a diverse group of polyphenols including ellagitannins, gallotannins, ellagic acid, and flavonoids, such as anthocyanins (*1*). Whereas there are numerous phytochemicals consumed in our diet, polyphenols constitute the largest group and have attracted much attention due to their antioxidant properties (*7*). In fact, the potential health benefits of plant foods are commonly linked to their polyphenol content.

Currently, there are a number of commercial ready-to-drink (RTD) polyphenol-rich beverages, which base their marketing strategies on antioxidant potency. Apart from PJ, other popularly consumed RTD polyphenol-rich beverages that claim high antioxidant potency include red wine, berry fruit juices (e.g., blueberry, black cherry, Concord grape, cranberry, etc.), apple juice, bottled tea beverages, and, recently, the Amazonian palm

* Author to whom correspondence should be addressed [telephone (310) 206 1987; fax (310) 206-5264; e-mail dheber@mednet.ucla.edu].
† University of California.
§ Rambam Medical Center.
# POM Wonderful, LLC.

10.1021/jf073035s CCC: $40.75 © 2008 American Chemical Society
Published on Web 01/26/2008

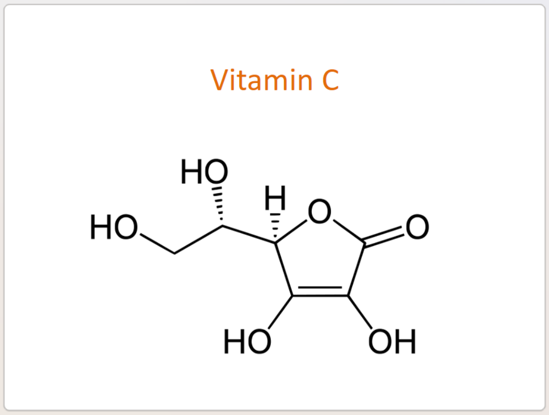
Vitamin C
HO
HO
H
O
O
HO
OH

1

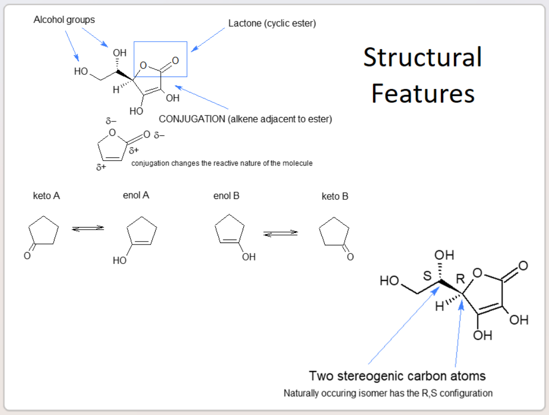
Structural Features
Alcohol groups
Lactone (cyclic ester)
CONJUGATION (alkene adjacent to ester)
conjugation changes the reactive nature of the molecule
keto A
enol A
enol B
keto B
Two stereogenic carbon atoms
Naturally occuring isomer has the R,S configuration

2

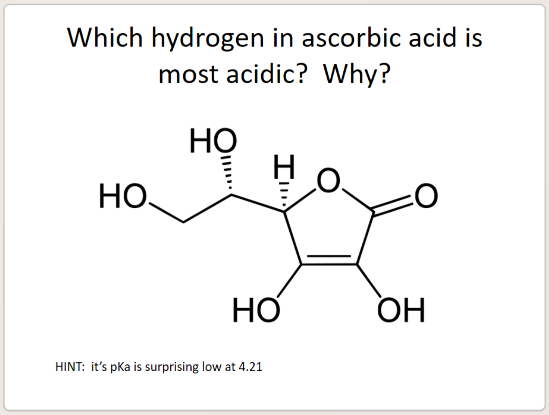
Which hydrogen in ascorbic acid is most acidic? Why?
HO
HO
H
O
O
HO
OH
HINT: it's pKa is surprising low at 4.21

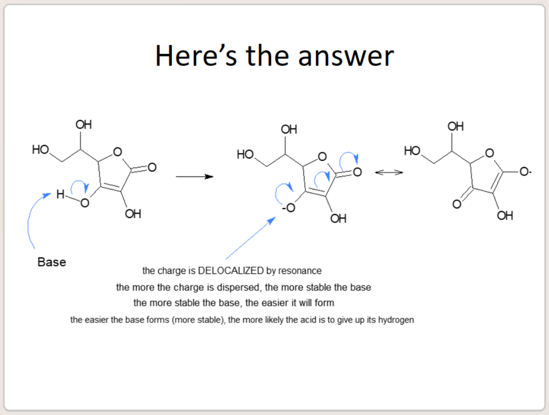
Here's the answer
Base
the charge is DELOCALIZED by resonance
the more the charge is dispersed, the more stable the base
the more stable the base, the easier it will form
the easier the base forms (more stable), the more likely the acid is to give up its hydrogen

Reactions – redox!

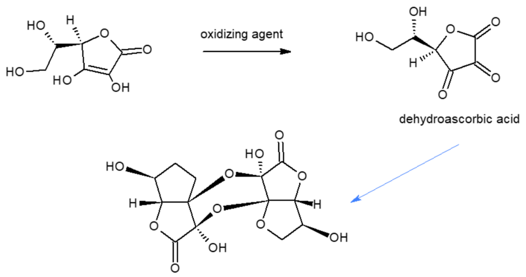

The dimer is the "actual" structure according to the J. Chem. Ed. article that we read

5

Kinetics – A Case Study: Degradation of ascorbic acid as a function of temperature

- The first question: What is the kinetic order for the degradation of ascorbic acid?
- An easy way to determine is to measure the concentration of ascorbic acid over time – can be easily determined by a spectroscopic technique (like the spectrophotometry that we did in lab) or by integration of the ascorbic acid peak on a chromatogram – higher concentrations give greater areas beneath the curve.
- So, if we know the initial concentration of ascorbic acid and then can measure the amount remaining over time, we can generate a series of graphs (see the next slide).

6

Zero, First, and Second Order Graphs

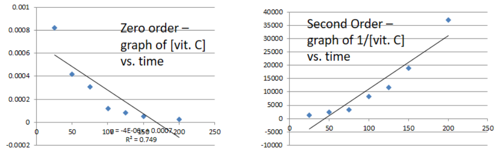

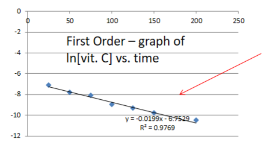

Notice how this graph is the only one of the three that is LINEAR – so for this reaction, the process of FIRST ORDER

Let's take a look at some experimental data

- This is for the degradation of ascorbic acid over time at three different temperatures
- Notice how the concentration of ascorbic acid is DECREASING (makes sense because it's degrading into something else).
- Also, notice how the RATE of degradation is changing at the different temperatures – higher temperatures lead to faster degradation – so, the rate constants at the different temperatures must also be different. We can extract the rate constants from the slopes of the individual linear graphs.
- These are graphs of ln[vit. C] vs. time (in minutes)

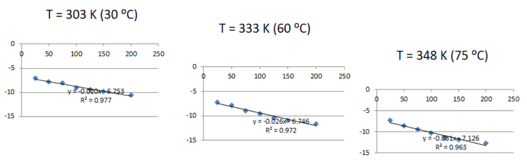

Finding Ea (Activation Energy)

See the following web site for a basic intro to the Arrhenius equation:
http://en.wikipedia.org/wiki/Arrhenius_equation

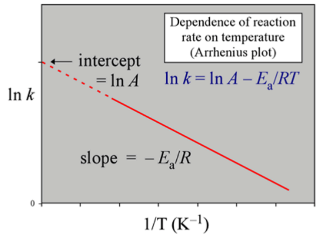

Using the natural log version of the Arrhenius equation shown in blue and by graphing ln(k) vs. 1/T In Kelvin), the activation energy can be found by dividing the slope by R (8.314 J/mol K).

9

So, here's the graph for our three rate constants at the three experimental temperatures:

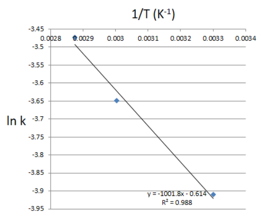

Slope = -Ea/R

$-1001\ K^{-1} = -Ea/8.314$ J/mol K

Ea = 8.322 KJ/mol

Activation Energy!

10

Summary for *J. Agric. Food Chem.*, **1999**, *29*, 1267-1274.

Name: ___________________________ **Primary Literature Summary**

1. **Skimming.** Identify a couple of key words that you think would best help you search the databases to find this paper.

1. ___________________________ 2. ___________________________

2. **Vocabulary.** List and define 3 words that you didn't understand when you first read the paper.

1. ___________________________

2. ___________________________

3. ___________________________

3. **Comprehension, section by section.**

Introduction: What is the "big idea", or overall purpose of the research?

Methods: What was measured and were the measurements appropriate for the questions the researcher was approaching?

Results: Rough sketch the most important figure in the paper and explain its importance in one sentence using plain English words.

Discussion:
What are the primary discoveries/findings in this paper?

What conclusion has the author drawn from the data? Briefly explain why you do or do not agree with the author's conclusion.

4. **Reflection and criticism.**
Identify the greatest strength in the way the paper is presented.

Identify one thing you would change/improve to increase the effectiveness of the paper.

5. **Chemistry content.**
(a) How does a GCMS (gas chromatography-mass spectrometry) instrument work?

(b) How was the acidity of liquid smoke determined? Why would the scientists want to know the acidity of the liquid smoke?

(c) Explain the strategy of liquid-liquid extraction of the liquid smoke using dichloromethane.

(d) Draw the structures for 2-methylbutanal and 2-pentanone. What do these compounds have in common?

(e) Draw the structures for any phenol and any ester from the paper.

Summary for *J. Agric. Food Chem.*, **2008**, *56*, 1415-1422.

Name: ____________________ **Primary Literature Summary**

1. **Vocabulary.** Define the keywords listed below. Structural drawings can also be used.

1. antioxidant

2. polyphenolic compound

3. flavonoid

4. TEAC assay

5. FRAP assay

6. DPPH assay

7. ORAC assay

2. **Comprehension, section by section.**

Introduction: (a) What is the "French paradox" in reference to compounds like resveratrol?

(b) What is the primary research **gap** for this study? Be careful – many of you are missing this point and need to identify/find the gap (that which is the problem or some aspect of the science that remains unresolved, ill-defined, or missing).

(c) What do the authors propose as their method to "**fill the gap**?"

Methods. (a) How is the total polyphenolic content determined? Be specific and be sure to describe its measurement in terms of GAEs (define this term).

(b) Describe the experimental strategy for performing the TEAC assay in your own words.

Results and Discussion: (a) According to **Table 1**, which is the juice of choice for potential antioxidant capacity? Are these results in agreement with **Table 4**?

(b) What do the results in **Figure 1** suggest about the relationship between polyphenolic content and antioxidant capacity?

(c) What is the relevance of the inhibition of LDL oxidation presented in **Table 2**?

(d) How do the authors corroborate their findings that PJ has the highest antioxidant capacity *in vitro*?

3. **Reflection and criticism.**
Do you think that the findings of this report might alter your choice of beverage in the future? Why or why not?

4. **Chemistry content.**
(a) Draw the structure and provide the name for the following compounds:

Resveratrol

Trolox

Gallic acid

Quercetin

BHT

(b) Describe the process by which these types of compounds as reported in this study work. Drawing structures inclusive of electrons is HIGHLY encouraged.

(c) What are the primary chemicals found in the Folin-Ciocalteau reagent? What is their chemical function?
